# Divergent SAGA complexes shape the *Toxoplasma* transcriptome for lytic cycle progression and host interaction

**DOI:** 10.1038/s41467-026-77153-4

**Published:** 2026-08-25

**Authors:** Dominique Cannella, Belen Pachano, Martina Shahinas, Lucid Belmudes, Jon deVries, Charlotte Corrao, Léa Pounot, Anne-Marie Hesse, Yohann Couté, Alexandre Bougdour, Christopher Swale, Mohamed-Ali Hakimi

**Affiliations:** 1 Institute for Advanced Biosciences (IAB), Team Host-pathogen interactions and immunity to infection, INSERM U1209, CNRS UMR5309, University Grenoble Alpes, Grenoble, France; 2 INSERM, UA13 BGE, CNRS, CEA, University Grenoble Alpes, Grenoble, France

**Keywords:** Parasite genomics, Epigenomics, Gene regulation

## Abstract

Histone acetylation governs *Toxoplasma gondii* gene expression, developmental plasticity and virulence, yet the organization and deployment of its acetyltransferase machinery remain poorly understood. Here, we show that *T. gondii* rewired this process using a plant-like system built around the acetyltransferase *Tg*GCN5b, which differs from the typical SAGA complex found in other eukaryotes. Interactome and structural analyses reveal a modular assembly that integrates multiple acetyltransferase (GNAT) enzymes and chromatin-reader proteins carrying PHD and PZP domains and Apetala-related transcriptional regulators, an organization unique to apicomplexan parasites. *Tg*GCN5b catalyzes a selective tri-site acetylation pattern on histone H3 at lysines 9, 14, and 18 that maintains open chromatin and sustains transcription of core metabolic, invasion, and virulence genes. Its conditional depletion disrupts these post-translational modifications, silencing key promoters and decoupling transcription from histone methylation. Genome-wide analyses further show that *Tg*GCN5b functions independently of the MORC/HDAC3 repressive pathway, defining a distinct regulatory circuit that connects chromatin acetylation with gene expression and developmental transitions. These findings reveal that the SAGA complex has been evolutionarily reconfigured in *T. gondii* from a plant origin, yet featuring divergent and apicomplexan-specific feature, positioning *Tg*GCN5b as a central regulator that links epigenetic control to parasite growth, adaptation, and virulence.

## Abstract (web summary)

Researchers show that the zoonotic parasite Toxoplasma gondii built a unique chromatin modifying complex to activate the genes that drive infection and virulence, revealing a potential weak spot for new treatments.

## Introduction

GCN5 (General Control Non-repressed protein 5) is a highly conserved histone acetyltransferase (HAT) that plays a pivotal role in transcriptional regulation across eukaryotes. As the catalytic core of the SAGA complex (Spt/Ada/Gcn5 acetyltransferase), GCN5 acetylates specific lysine residues on histone H3, promoting chromatin relaxation and enhancing promoter accessibility^1^. The SAGA complex is a multifunctional coactivator composed of distinct modules that coordinate histone acetylation with other chromatin-modifying activities, including histone deubiquitination, transcription factor recruitment, and interactions with the basal transcription machinery^2,3^. By integrating these functions, SAGA serves as a central hub linking epigenetic modifications to dynamic transcriptional responses^4,5^. The presence of GCN5 in all eukaryotic lineages underscores its essential and evolutionarily conserved role in cell biology^6–8^.

In *Toxoplasma gondii*, an unusual gene duplication has produced two GCN5 paralogs with divergent roles. *Tg*GCN5a mediates transcriptional adaptation to alkaline stress yet remains dispensable for tachyzoite growth under standard conditions^9^. Notably, the enzyme localizes exclusively to telomeres, and its loss provokes telomere dysfunction, slowing replication and predisposing parasites to latency^10^. In sharp contrast, *Tg*GCN5b is indispensable for parasite viability, with genetic disruption attempts proving lethal^11^. It is thought to sustain a broad, constitutive program that promotes growth and developmental transitions^11,12^. Insights into *Tg*GCN5b have largely come from early studies using either an inducible dominant-negative mutant (E703G) that abolishes enzymatic activity^11^ or ectopic expression of a second, tagged copy to map the *T. gondii* SAGA complex^11,13^. These strategies revealed distinctive molecular features and interaction partners, but were performed in parasites still expressing the native enzyme. As a result, residual activity may have masked critical phenotypes, and the tagged-bait strategy could skew the interactome by diluting scarce partners between endogenous and ectopic *Tg*GCN5b. By generating a conditional knockdown and endogenous knock-in of *Tg*GCN5b, we now provide a more accurate functional framework that both corroborates and revises earlier models.

More importantly, we discovered that *Toxoplasma* is reprogramming histone acetylation through a plant-like *Tg*GCN5b-centered system, where multi- Gcn5-related N-acetyltransferase (GNAT) catalysis and reader-rich scaffolds cooperate to establish a distinctive H3K9–K14–K18 acetylation signature. *Tg*GCN5b acts as the pivotal integrator of chromatin accessibility and gene regulation, coordinating essential programs of metabolism, virulence, and developmental competence that ensure parasite survival across its life cycle.

## Results

### Divergent and promiscuous architectures of SAGA in *T. gondii*

**Fig. 1:**
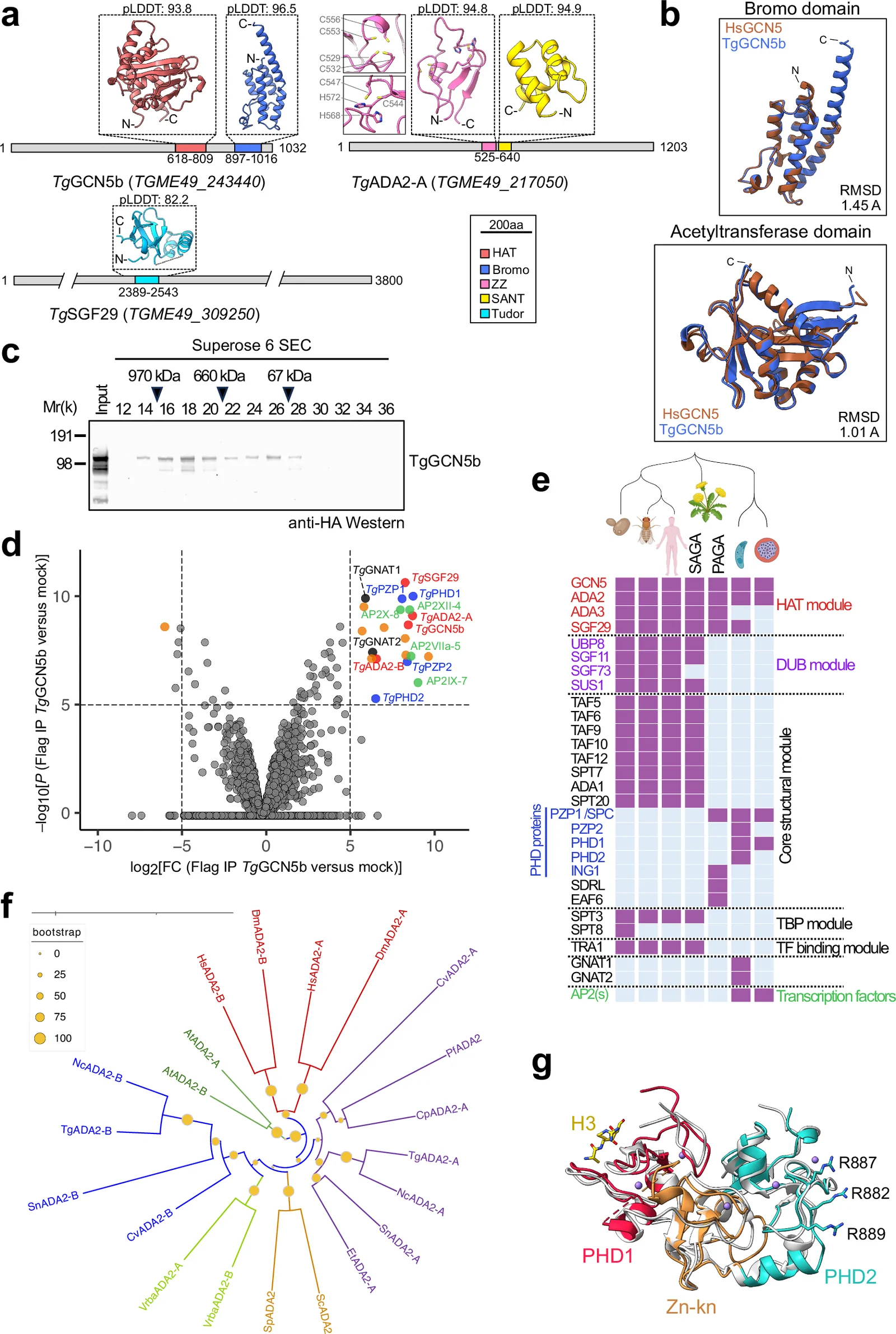
Composition, size, and structural features of *Tg*GCN5b-containing complexes. **a** AlphaFold2 models of *Tg*ADA2-A, *Tg*GCN5b, and *Tg*SGF29 with domains annotated according to predicted structures and known functional motifs. Corresponding pLDDT scores are shown. **b** Structural alignment of human *Hs*GCN5 and *Tg*GCN5b reveals conservation of the HAT (RMSD = 1.01 Å) and bromodomain (RMSD = 1.45 Å) regions. **c** Size-exclusion chromatography (SEC) of Flag-purified *Tg*GCN5b complexes shows a prominent anti-HA signal in fractions 16–20 (~800 kDa), supporting incorporation of *Tg*GCN5b-HAFlag into a large multiprotein assembly. **d** Volcano plot showing differential protein abundance in *Tg*GCN5b-HAFlag versus mock co-immunoprecipitation eluates analyzed by MS-based quantitative proteomics. The plot displays log2(fold changes) and −log10 unadjusted *p* values obtained from two-sided moderated *t*-tests implemented in limma. Dashed lines indicate enrichment and significance thresholds; labeled proteins on the right are the most strongly enriched *Tg*GCN5b-complex components. **e** Comparison of the modular organization of yeast, Drosophila, and human SAGA complexes, plant SAGA/PAGA complexes, and *Tg*GCN5b-associated complexes from *T. gondii* and *P. falciparum*. Proteins are arranged by conserved SAGA-like modules, highlighting conserved and parasite-specific components. Pink and blue squares indicate presence and absence, respectively. Created in BioRender. Hakimi, M. (2026) https://BioRender.com/u9itcxe. **f** Maximum-likelihood tree generated from full-length ADA2 sequences across representative eukaryotes. TgADA2-A clusters within Apicomplexa on a well-supported branch distinct from fungal and metazoan orthologues. Circle sizes indicate bootstrap support. *Cryptosporidium parvum* Cp, *Toxoplasma gondii* Tg, *Plasmodium falciparum* Pf, *Neospora caninum* Nc, *Eimeria tenella* Et, *Arabidopsis thaliana* At, *Drosophila melanogaster* Dm, *Homo sapiens* Hs, *Chromera velia* Cv, *Sarcocystis neurona* Sn, *Vitrella brassicaformis* Vbra. **g** AlphaFold-predicted structure of *Tg*PZP1 (TGME49_234900 residues 701-946) with PHD1 in pink, Zn-Kn in a pale shade, and PHD2 in aquamarine. Putative DNA-binding positively charged PHD2 residues are shown as labeled sticks. The model was superposed in ChimeraX onto human BRPF1 PZP bound to the histone H3 tail (PDB ID: 6U04). The H3 tail is shown in yellow, and the zinc ions are violet spheres. Source data are provided as a Source Data file.

**Fig. 2:**
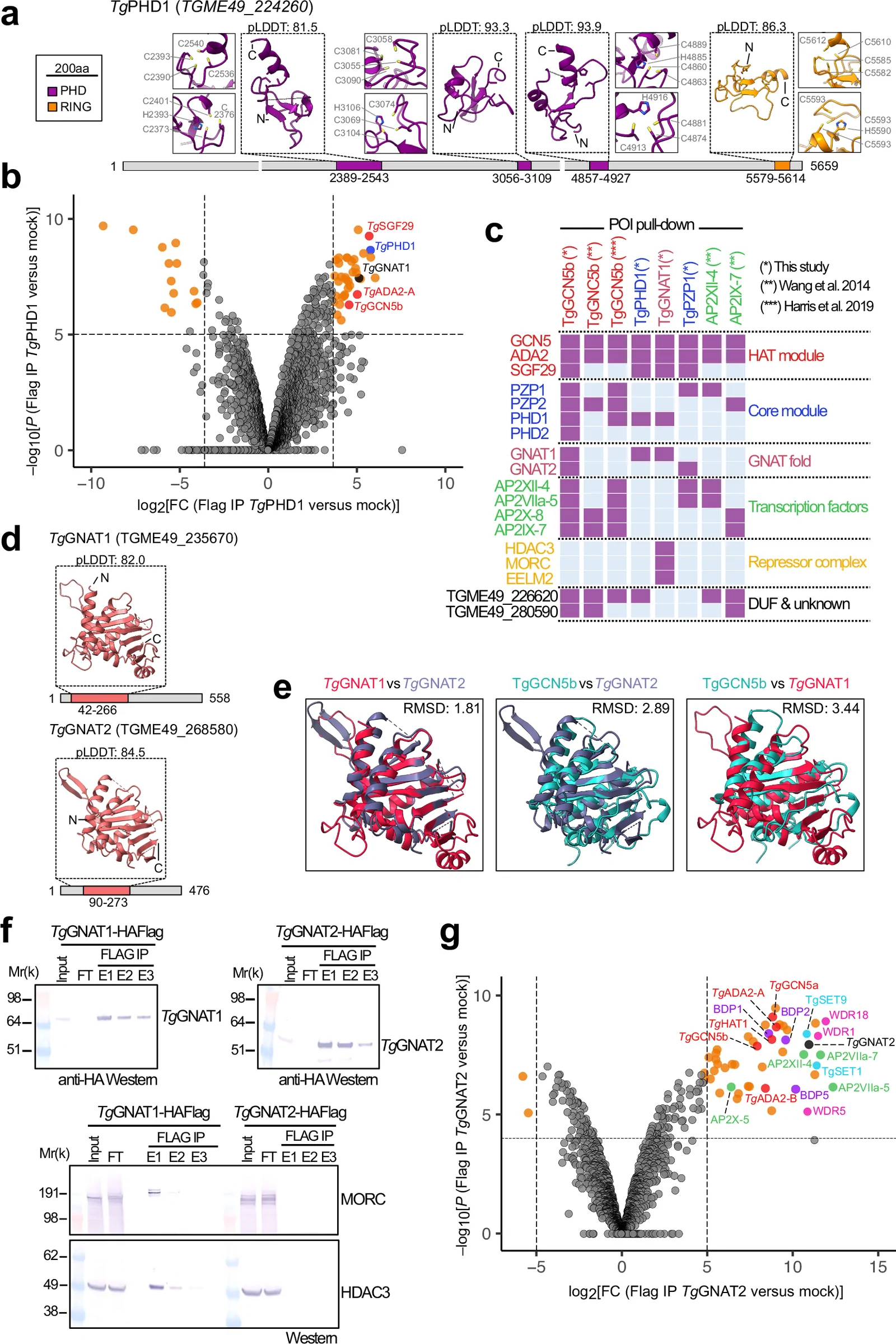
Conservation and divergence within the *Tg*GCN5b interactome. **a** AlphaFold-predicted domain organization of *Tg*PHD1. Per-residue confidence scores (pLDDT) support the structural integrity of the PHD and RING domains. Conserved cysteine- and histidine-rich motifs characteristic of chromatin-binding modules are highlighted. **b** Volcano plot showing differential protein abundance in *Tg*PHD1–HA–Flag versus mock co-immunoprecipitation eluates analyzed by MS-based quantitative proteomics. The plot displays log2(fold changes) and −log10 unadjusted *p* values obtained from two-sided moderated *t*-tests implemented in limma. Dashed lines indicate enrichment and significance thresholds; labeled proteins on the right are the most strongly enriched *Tg*PHD1/*Tg*GCN5b-complex components. **c** Schematic representation of the interaction networks generated using *Tg*GCN5b partners as bait in a reverse pull-down/MS analysis, compared with previously reported *Tg*GCN5b interactomes^11,13^. Proteins detected in each dataset are organized according to conserved apicomplexan SAGA-like modules, highlighting the versatility and dynamic nature of the *Tg*GCN5b regulatory assemblies, which form multiple discrete complexes rather than a single stable entity. Pink and blue squares indicate presence and absence, respectively. **d** AlphaFold-predicted domain organizations of *Tg*GNAT1 and *Tg*GNAT2. pLDDT scores support the structural integrity of their GNAT catalytic domains. **e** Comparison of *Tg*GNAT1, *Tg*GNAT2, and *Tg*GCN5b acetyltransferase catalytic domains. RMSD values (3.44 Å for *Tg*GCN5b vs. *Tg*GNAT1, 2.89 Å for *Tg*GCN5b vs. *Tg*GNAT2, and 1.81 Å for *Tg*GNAT1 vs. *Tg*GNAT2) highlight conserved GNAT folds while revealing divergence from the canonical *Tg*GCN5b HAT module. **f** *Tg*GNAT1- and *Tg*GNAT2-associated proteins were purified by Flag chromatography from extracts of HFFs infected with RH *Tg*GNAT1-HA-Flag or RH *Tg*GNAT2-HA-Flag. Fractions were immunoblotted with anti-HA antibodies to detect the tagged baits and with anti-HDAC3 or anti-MORC antibodies to detect associated proteins. Input (In), flow-through (FT), Flag-IP, and the indicated eluates are shown. **g** Volcano plot of quantitative proteomic analysis comparing *Tg*GNAT2-HAFlag and mock co-immunoprecipitation eluates. The plot displays log2(fold changes) and −log10 unadjusted *p* values obtained from two-sided moderated *t*-tests implemented in limma. Dashed lines indicate enrichment and significance thresholds. Highlighted interactors include TgGCN5b-complex components (red), AP2 transcription factors (green), a bromodomain protein (BDP; pink), and a SET-domain histone lysine methyltransferase (blue).

GCN5-type histone acetyltransferases, conserved across eukaryotes, act as the catalytic core of SAGA through a central HAT domain and adjacent bromodomain that couple enzymatic activity with substrate recognition (Fig. 1a). AlphaFold prediction^14^ for *Tg*GCN5b reveals these domains in configurations nearly identical to human orthologues (r.m.s.d. <1.5 Å; Fig. 1b). Unlike most species, *T. gondii* GCN5s carry long, poorly conserved N-terminal extensions distinct from each other and from *Plasmodium* GCN5, suggesting divergent SAGA architecture. To overcome limitations of earlier ectopic mapping^11,13^, we generated an endogenous knock-in, capturing *Tg*GCN5b in its native context to refine the definition of SAGA composition (Supplementary Data 1). Flag-based affinity purification followed by size-exclusion chromatography (SEC) resolved two peaks: one at the predicted size of *Tg*GCN5b alone (around fraction 26), and a broader peak ranging from 1 MDa to 660 kDa (fractions 14 to 20), indicative of stable *Tg*GCN5b-associated assemblies that resist stringent washing (0.5 M NaCl, 0.1% NP-40; Fig. 1c). Mass spectrometry (MS)-based proteomic analysis of the *Tg*GCN5b immunoprecipitated FLAG eluate, compared with a mock control, revealed a high-confidence enrichment of several proteins (Figs. 1d, e and 2), most experimentally localized by HyperLOPIT to the nucleus and predominantly associated with chromatin (Supplementary data 2)^15^. Comparison with ectopic datasets^11,13^ revealed a shared core of 10 proteins (Fig. 2c). Several were hypothetical proteins, previously observed in pull-downs but whose function could not be predicted using only sequence homology. We also identified seven interactors unique to native conditions (Supplementary data 2), highlighting the importance of interrogating SAGA in its physiological context.

At first glance, *Tg*GCN5b-associated assemblies share little resemblance to conserved SAGA or to other GCN5 complexes such as SLIK/SALSA or ATAC^16,17^. *T. gondii* encodes two ADA2 paralogues, *Tg*ADA2-A and *Tg*ADA2-B, indicative of a divergent evolutionary path within apicomplexan parasites. Phylogeny resolves a clear duplication, with apicomplexan sequences split between two clades, while fungi collapse to a single ADA2 lineage, indicating secondary loss. *Plasmodium* retains only one ADA2, nested within the ADA2-A clade, consistent with lineage-specific loss of ADA2-B (Fig. 1f). These relationships point to an early apicomplexan expansion of ADA2 followed by species-specific pruning. In tachyzoites, native purifications consistently recovered *Tg*ADA2-A as the principal partner of *Tg*GCN5b, whereas *Tg*ADA2-B was detected only at lower abundance near the limit of statistical confidence, suggesting paralogue specialization (Fig. 1d and Supplementary Fig. 1a). Strikingly, the HAT module assembles without an ADA3 homolog but incorporates a divergent *Tg*SGF29 subunit, purified here exclusively under native conditions (Fig. 1d and Supplementary data 2). The identification of a divergent TUDOR-like domain was not achieved by BLAST but instead relied on a structural homology search using AlphaFold/Foldseek (Fig. 1a). In other eukaryotes, similar folds bind H3K4me2/3 to recruit SAGA to active chromatin^18^ and in the case of *T. gondii*, this protein likely acts within an ADA3-independent, H3K4-directed HAT module. This substitution reveals an adaptive remodeling of the HAT module that preserves recognition of active chromatin while bypassing classical ADA scaffolds. By contrast, *Plasmodium Pf*GCN5 associates with *Pf*ADA2, but to date, no SGF29 orthologue has been identified^19^ (Fig. 1e).

Beyond the HAT module, opisthokont SAGA harbors a multi-subunit Deubiquitinase (DUB) centered on Ubp8/USP22, which catalyzes deubiquitylation of mono-ubiquitylated histone H2B, coupling transcription to chromatin remodeling^20^. In contrast, our proteomic analyses failed to identify orthologues of the canonical DUB module components SGF11, SUS1 and SGF73. Although a Ubp8-like protein is encoded in the *T. gondii* genome (*TGME49_226940*), it was not specifically enriched in any *Tg*GCN5b-associated complexes and therefore cannot currently be assigned to SAGA. No subunits of the canonical SPT module co-purified with *Tg*GCN5b, and *Tg*Tra1 (TGME49_268370) was not recovered with sufficient confidence to support its association with SAGA. *Tg*Tra1 is markedly larger (~850 kDa) than its yeast and metazoan counterparts, yet AlphaFold predicts that it preserves the canonical HEAT, FAT, and kinase domains, underscoring deep structural conservation. In other eukaryotes, Tra1 serves as the principal activator-binding platform, engaging factors such as Gal4 in yeast^21^ and p53 in mammals^22^. Whether *Tg*Tra1 fulfills a similar function in *T. gondii* and whether it associates with SAGA under specific conditions remain important questions for future studies.

A striking innovation of *T. gondii* SAGA is the emergence of four subunits bearing plant homeodomain (PHD, Fig. 2a and Supplementary Fig. 1b) or composite PZP domains, features absent from opisthokonts but conserved in *Plasmodium* and in plant PAGA complex^23^ (Fig. 1g and Supplementary Fig. 1c). *Tg*PHD1 and *Tg*PZP1 correspond to PfPHD1 and PfPHD2 in *P. falciparum*^19^, respectively, whereas *Tg*PHD2 and *Tg*PZP2 are unique to *T. gondii*. The expansion of PHD and PZP modules suggests an evolutionary strategy to enhance chromatin-reader diversity, enabling the apicomplexan SAGA to interpret a broader range of histone states. In mammals, the PZP domain of BRPF1 exemplifies this architecture: two PHD fingers bridged by a zinc knuckle that engage both histone H3 tails and DNA^24^, thereby conferring dual chromatin-anchoring capacity (Fig. 1g). By analogy, apicomplexan PZPs are likely to act as chromatin-tethering platforms. Notably, *Tg*PZP1 and *Tg*PZP2 selectively co-purified with distinct AP2 heterodimers: AP2XII-4/AP2VIIa-5 and AP2IX-7/AP2X-8, respectively (Fig. 2c and Supplementary Fig. 1d–f). Reciprocal IPs with AP2XII-4 and AP2IX-7 as baits confirmed these associations^13^ (Fig. 2c). These findings reveal an unanticipated architectural principle in which PZP-containing proteins provide dedicated docking platforms that couple *Tg*GCN5b to lineage-specific ApiAP2 transcription factors, potentially compensating for the absence of the canonical SPT module found in opisthokont SAGA complexes. Remarkably, TgPZP1 shares homology with SPC, a scaffold subunit of the plant PAGA complex^23^ (Supplementary Fig. 1e), raising the possibility that apicomplexan SAGA and plant PAGA share a common evolutionary origin.

Finally, two previously uncharacterized proteins, TGME49_235670 and TGME49_268580, were recovered exclusively under native conditions (annotated *Tg*GNAT1 and *Tg*GNAT2, respectively, in Fig. 1d, e and Supplementary data 2). Although BLAST searches failed to identify any domains, structure-based homology analysis using AlphaFold-predicted models coupled with Foldseek revealed that both possess Gcn5-related N-acetyltransferase (GNAT) folds (Fig. 2d). Structural comparisons further support their distinctiveness: *Tg*GNAT1 (TGME49_235670) and *Tg*GNAT2 (TGME49_268580) align closely with each other (r.m.s.d. = 1.81 Å), yet diverge more substantially from *Tg*GCN5b (r.m.s.d. = 2.89 Å for *Tg*GNAT2 vs *Tg*GCN5b; 3.44 Å for *Tg*GNAT1 vs *Tg*GCN5b) (Fig. 2e). These metrics indicate a distinct GNAT subclass that likely evolved in apicomplexans to fulfill roles not covered by canonical GCN5 paralogues.

The coexistence of multiple GNATs within a single SAGA-like complex is, to our knowledge, unprecedented: whereas opisthokont SAGA relies on a single GCN5, apicomplexans appear to have evolved a multi-GNAT architecture. The abundant incorporation of *Tg*GNAT1 alongside GCN5b suggests an expanded catalytic repertoire, implying that *T. gondii* SAGA exploits specialized GNAT paralogues to diversify substrate specificity and chromatin engagement, an innovation without parallel in other eukaryotes. Endogenous epitope-tagging revealed that *Tg*GNAT1 co-purifies with the canonical HAT module (*Tg*GCN5b, *Tg*ADA2-A, *Tg*SGF29) and *Tg*PHD1. Unexpectedly, it also associates with a minimal repressive MORC complex containing an EELM2 protein and the histone deacetylase HDAC3 (Fig. 2f and Supplementary data 2). Consistently, reverse immunoprecipitation of *Tg*PHD1 recovered the intact HAT module together with *Tg*GNAT1, further consolidating their assembly into a native complex (Fig. 2b, c and Supplementary data 2). Such dual interactions place *Tg*GNAT1 at the crossroads of chromatin activation and repression, raising the provocative possibility of protein moonlighting: a single enzyme dynamically partitioning between complexes of opposing function. This architectural innovation suggests that *T. gondii* SAGA has evolved not merely to acetylate chromatin, but to flexibly balance activating and repressive cues through a GNAT paralogue with bifunctional potential.

The role of *Tg*GNAT2 appears fundamentally different from that of the core SAGA components. Whereas *Tg*GNAT2 was recovered in *Tg*GCN5b pull-downs, reciprocal purification of *Tg*GNAT2 revealed a much broader interaction landscape, including three lysine acetyltransferases (*Tg*GCN5b, *Tg*GCN5a, and *Tg*HAT1), two lysine methyltransferases (*Tg*SET1 and *Tg*SET9, Supplementary Fig. 1g), multiple bromodomain-containing proteins (*Tg*BDP1, *Tg*BDP2, and *Tg*BDP5)^25^, and a diverse set of ApiAP2 transcription factors (Fig. 2g and Supplementary data 2). This interactome extends well beyond the boundaries of the *Tg*GCN5b complex and suggests that *Tg*GNAT2 functions as a chromatin-associated hub integrating distinct writer, reader, and transcription factor networks. Rather than acting as a dedicated SAGA subunit, *Tg*GNAT2 may facilitate communication between multiple epigenetic machineries and transcriptional regulators. One intriguing possibility is that *Tg*GNAT2 participates in the dynamic assembly of chromatin regulatory complexes, serving as a reservoir or exchange platform from which catalytic activities can be recruited according to the developmental or transcriptional needs of the parasite.

Altogether, our data define a streamlined apicomplexan SAGA complex centered on a stable stoichiometric core comprising the HAT module (*Tg*GCN5b, *Tg*ADA2-A, and *Tg*SGF29), the chromatin readers *Tg*PHD1 and *Tg*PZP1, and a transcription factor module dominated by the AP2XII-4/AP2VIIa-5 heterodimer. All ranked among the most enriched proteins after tandem FLAG–HA purification, supporting their incorporation into a common assembly (Supplementary Fig. 1a). In contrast, *Tg*GNAT1, *Tg*GNAT2, and other interactors were depleted after the second purification, suggesting transient, substoichiometric, or accessory roles. This architecture distinguishes apicomplexan SAGA from opisthokont complexes while revealing striking parallels with plant PAGA.

### Loss of *Tg*GCN5b disrupts endodyogeny, parasite architecture, and lytic growth

**Fig. 3:**
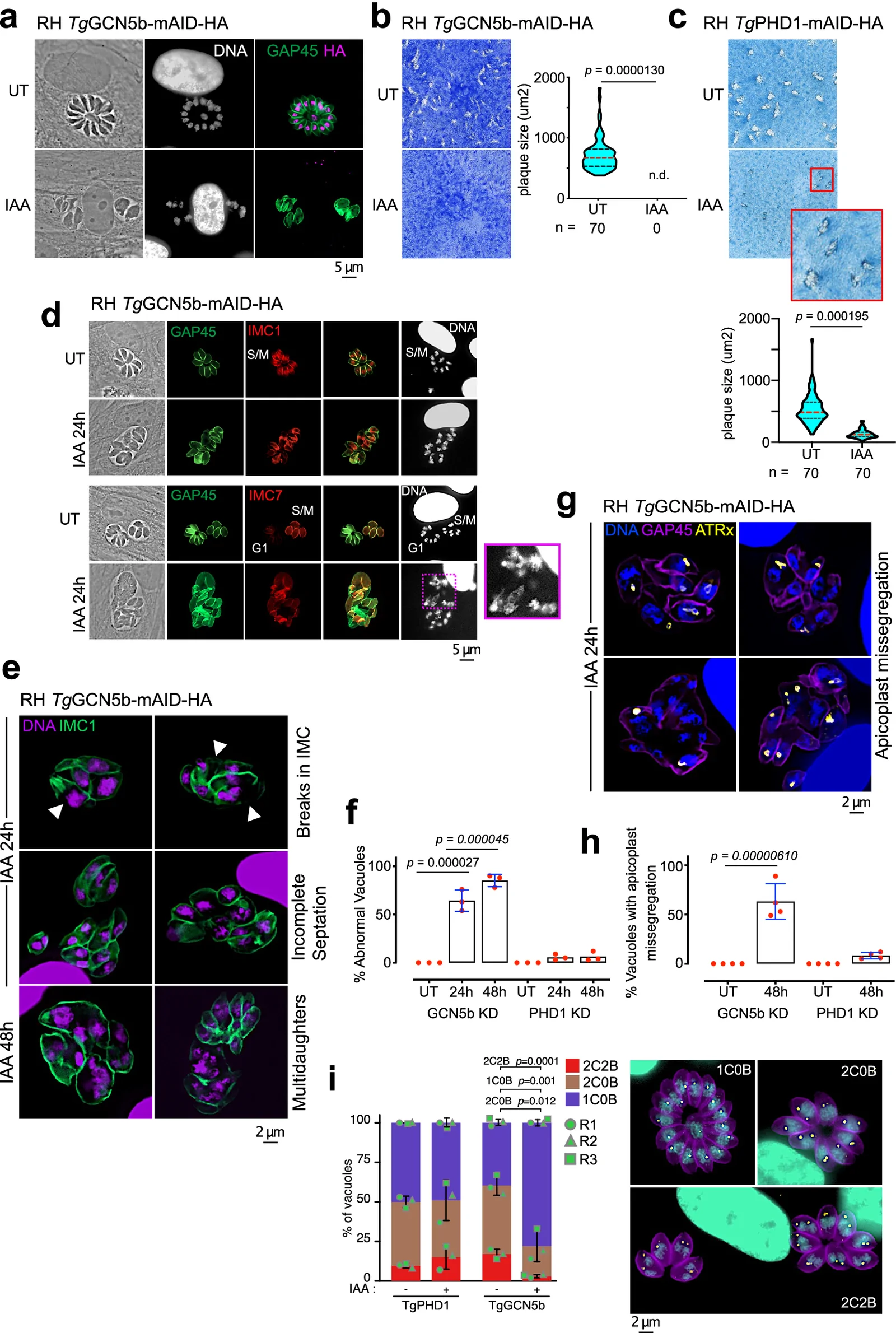
*Tg*GCN5b depletion disrupts parasite replication, daughter-cell budding, and apicoplast segregation. **a** Immunofluorescence of untreated (UT) or auxin-treated (IAA) *Tg*GCN5b–mAID–HA stained for HA (pink), GAP45 (green) and DNA (DAPI). GAP45 delineates the mother-cell pellicle and daughter buds. Scale, 5 μm. Plaque assays of *Tg*GCN5b–mAID–HA (**b**) and *Tg*PHD1–mAID–HA (**c**) parasites cultured with or without IAA. After inoculation of 500 parasites and growth for 7 days, representative plaques and plaque-area quantifications are shown. *Tg*GCN5b depletion abolished plaque formation, whereas *Tg*PHD1 depletion reduced plaque size. Areas are shown as violin plots (*n* = 70 plaques for UT and *Tg*PHD1 IAA conditions; no plaques were detected for *Tg*GCN5b IAA). Statistical significance was assessed using an unpaired two-sided Student’s *t*-test. n.d., not detected. **d** Immunofluorescence of untreated or 24-h IAA-treated *Tg*GCN5b–mAID–HA parasites stained for GAP45 (green), IMC1 or IMC7 (red), and DNA (DAPI), showing abnormal budding and cell-cycle progression after depletion. Scale bars, 5 μm. **e** Representative examples of replication defects observed following depletion, including breaks in the IMC (arrowheads), incomplete septation, and formation of multiple daughters after 24–48 h of IAA treatment. Parasites were stained with anti-IMC1 (in green) and DAPI (pink). Scale, 2 μm. **f** Quantification of abnormal vacuoles after *Tg*GCN5b or *Tg*PHD1 depletion. **g** *Tg*GCN5b-depleted parasites stained for ATRX (apicoplast; yellow), GAP45 (pink) and DNA (DAPI; blue), showing frequent apicoplast missegregation. Scale, 2 μm. **h** Quantification of apicoplast missegregation defects after *Tg*GCN5b or *Tg*PHD1 depletion. *Tg*GCN5b knockdown significantly increases apicoplast segregation defects compared with control parasites. For **f**, **h**, data are presented as the mean ± s.d. of 3 independent biological replicates, with 30 vacuoles analyzed per replicate. Statistical significance was assessed using an unpaired two-tailed Student’s *t*-test. **i** Centrosome/budding stages were classified as 1C0B (G1), 2C0B (S phase) or 2C2B (M/C phase) using anti-Centrin staining. Data are mean ± s.d. from *n* = 3 biological replicates (50 vacuoles each). The genotype × IAA interaction was assessed by two-way repeated-measures ANOVA, followed by two-sided paired t-tests with Holm correction across four within-category comparisons. Source data are provided as a Source Data file.

To assess the role of *Tg*GCN5b in parasite fitness, we performed plaque assays using an inducible TgGCN5b knockdown (KD) line (Supplementary data 1). Addition of indole-3-acetic acid (IAA) led to rapid and specific depletion of the mAID-HA-tagged protein, as demonstrated by immunofluorescence analysis (Fig. 3a). *Tg*GCN5b depletion completely prevented plaque formation, revealing an essential function for this enzyme during lytic cycle progression (Fig. 3b). By comparison, *Tg*PHD1 depletion reduced plaque size but did not eliminate plaque formation, indicating a less stringent requirement for parasite growth (Fig. 3c). Given the severity of this phenotype, we next focused on *Tg*GCN5b and examined intracellular parasites by co-staining GAP45 with the inner membrane complex markers IMC1 and IMC7 (Fig. 3d). GAP45 labels nascent daughter buds during early-to-intermediate stages of endodyogeny, IMC1 marks the developing daughter cytoskeletal scaffold and parasite morphology, whereas IMC7 distinguishes mature mother parasites from newly formed daughters, collectively enabling assessment of endodyogeny and cell cycle progression^26^. Following IAA treatment, *Tg*GCN5b-depleted parasites exhibited profound defects in intracellular replication. Vacuoles frequently contained asynchronously dividing parasites, abnormal daughter cell organization, and severe perturbations of IMC biogenesis (Fig. 3d, e). These abnormalities included breaks in the IMC network, incomplete septation between daughter parasites, and the formation of multiple daughter buds within a single mother cell (Fig. 3e). Quantification showed that approximately 60% of vacuoles displayed at least one of these defects after 24 h of depletion, increasing to nearly 80% after 48 h (Fig. 3f). In contrast, only a small fraction of *Tg*PHD1-depleted vacuoles exhibited comparable abnormalities (Fig. 3f). DAPI staining further revealed defects in nuclear organization following *Tg*GCN5b depletion. Whereas untreated parasites displayed the characteristic compact nuclei of replicating tachyzoites, *Tg*GCN5b-depleted parasites exhibited dispersed and irregularly distributed DNA associated with aberrant daughter structures, consistent with defective nuclear segregation during endodyogeny (Fig. 3d, magnified inset). Because faithful organelle inheritance is tightly coordinated with daughter cell formation, we next examined apicoplast segregation using ATRx as a marker (Fig. 3g). *Tg*GCN5b depletion resulted in frequent apicoplast missegregation events, affecting approximately 60% of vacuoles after 48 h of treatment, whereas this phenotype remained rare in *Tg*PHD1-depleted parasites (Fig. 3g, h). Together, these results demonstrate that *Tg*GCN5b is essential for coordinated daughter cell formation, nuclear segregation, cytokinesis, and organelle inheritance. The combined disruption of these processes likely underlies the complete inability of *Tg*GCN5b-depleted parasites to complete the lytic cycle (Fig. 3b). To determine whether these structural and replication defects reflected a broader imbalance in cell-cycle progression, we next quantified centrosome duplication and daughter budding using Centrin staining. Parasites were classified as 1C0B, corresponding to one centrosome and no daughter bud, G1; 2C0B, two centrosomes and no bud, S phase; or 2C2B, two centrosomes and two buds, M/C phase. *Tg*GCN5b depletion significantly altered the distribution of these cell-cycle categories, with an accumulation of 1C0B/G1 parasites and a concomitant reduction in parasites progressing toward budding stages, whereas *Tg*PHD1 depletion had only a limited effect (Fig. 3i). Thus, *Tg*GCN5b loss not only disrupts daughter-cell architecture and organelle inheritance but also impairs progression through the early cell-cycle program.

### *Tg*GCN5b-containing SAGA imprints a unique H3K9–K14–K18 acetylation signature

**Fig. 4:**
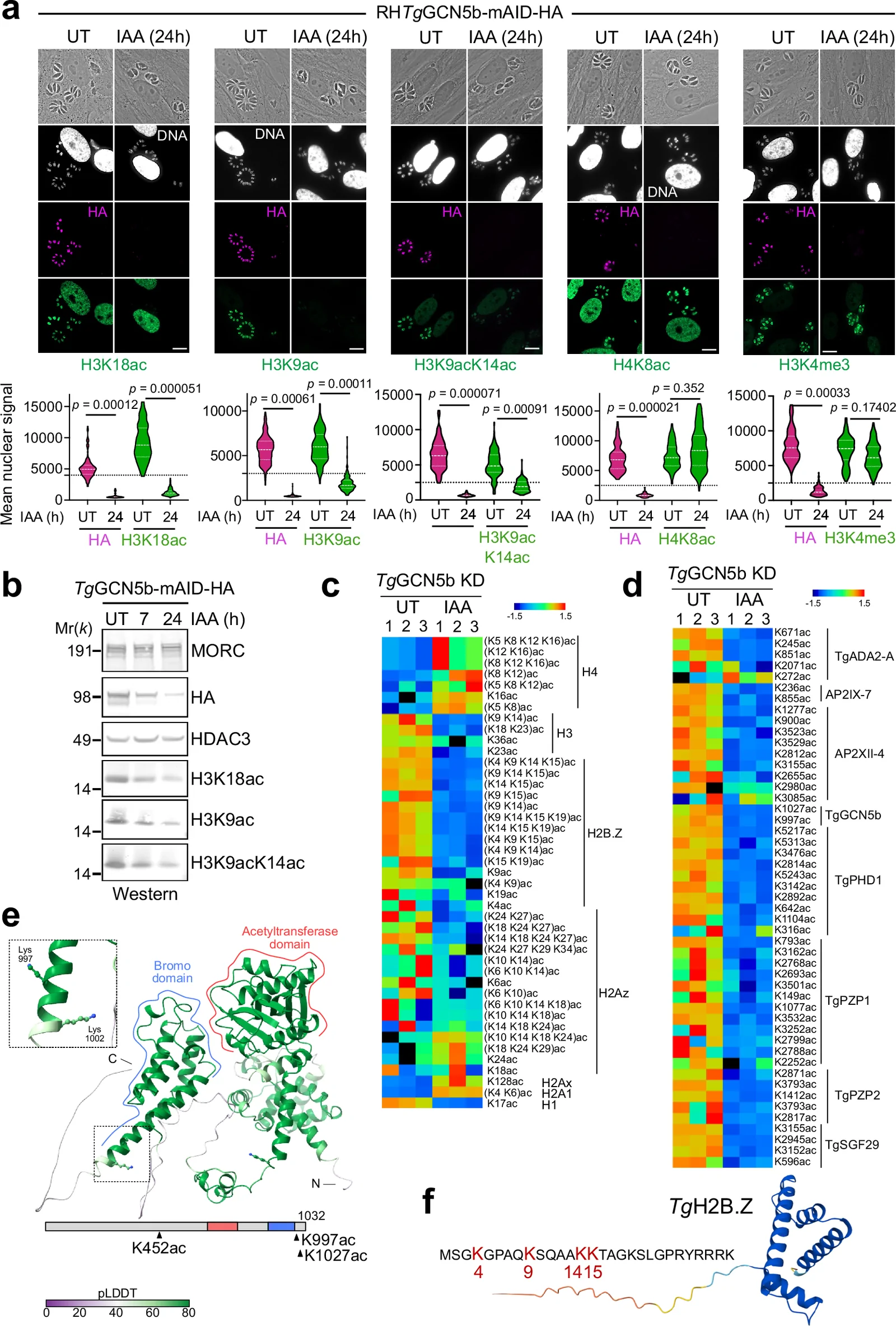
*Tg*GCN5b imprints a unique H3K9–K14–K18 acetylation signature and acetylates histone and non-histone chromatin-associated proteins. **a** Immunofluorescence of *Tg*GCN5b-mAID-HA grown in the absence (UT) or presence of auxin (IAA) for 24 h. Parasites were stained with anti-HA and antibodies recognizing H3K18ac, H3K9ac, H3K9acK14ac, H4K8ac, or H3K4me3. Nuclear fluorescence intensities were quantified and are shown below each panel. *Tg*GCN5b depletion results in a marked reduction of H3K18ac, H3K9ac, and H3K9acK14ac levels, whereas H4K8ac and H3K4me3 remain largely unaffected. Data are presented as violin plots showing individual nuclei. Statistical significance was determined using an unpaired two-tailed Student’s t-test; n.s., not significant. Scale bars, 10 μm. **b** Western blot analysis of *Tg*GCN5b-mAID-HA parasites treated with IAA for the indicated times. Blots were probed with antibodies against HA, H3K18ac, H3K9ac, and H3K9acK14ac, with HDAC3 and MORC serving as loading controls. Acute depletion of *Tg*GCN5b leads to a progressive reduction in histone H3 acetylation. Molecular weights (Mr) are indicated on the left. **c** Heatmap showing relative abundance of acetylated histone peptides quantified by mass spectrometry in untreated (UT) and *Tg*GCN5b-depleted (IAA, 24 h) parasites. Acetylation sites on H3, H4, H2B.Z, H2Az, H2A1, and H1 are displayed. Values represent normalized abundance changes across three biological replicates. **d** Heatmap of acetylated lysine residues identified on components of the *Tg*GCN5b complex following *Tg*GCN5b depletion. Acetylation levels were determined by quantitative mass spectrometry in untreated and IAA-treated parasites (Supplementary data 2). Several acetylation sites on *Tg*GCN5b and associated proteins exhibit decreased abundance upon *Tg*GCN5b depletion, suggesting direct or indirect dependence on *Tg*GCN5b acetyltransferase activity. **e** AlphaFold structural model of *Tg*GCN5b highlights the bromodomain and acetyltransferase domain. Acetylated lysine residues identified by mass spectrometry are mapped onto the protein, including K452, K997, and K1027. Insets show the location of acetylated lysines within the predicted structure. The confidence of the structural prediction is indicated by the pLDDT score. **f** Schematic representation of the N-terminal tail of histone variant *Tg*H2B.Z showing acetylated lysine residues identified by mass spectrometry. Conserved lysines K4, K9, K14, and K15 are indicated in red. Source data are provided as a Source Data file.

The marked divergence of the *T. gondii* SAGA complex prompted us to investigate whether *Tg*GCN5b has also undergone functional specialization, resulting in a distinct histone acetylation signature. To identify *Tg*GCN5b substrates, we first assessed its contribution to the acetylation of evolutionarily conserved lysine residues using immunofluorescence assays and western blot analyses. Acute loss of the enzyme leads to a marked reduction of H3K9ac and H3K14ac within parasite nuclei, consistent with previous findings^11,19^, and most notably reduces H3K18ac to undetectable levels, while leaving H4K8ac and H3K4me3 unaffected (Fig. 4a, b). The selective collapse of H3K18ac, while other HAT-sensitive marks remain stable, underscores a direct catalytic dependence rather than a secondary chromatin effect or global transcriptional shutdown. The disappearance of H3K18ac contradicts conclusions drawn from the dominant-negative E703G mutant^11^ and instead defines a *Tg*GCN5b-dependent H3K9–K14–K18 acetylation signature. These findings further suggest that the *P. falciparum* ortholog *Pf*GCN5, previously linked only to H3K9ac/H3K14ac^19^, may also target H3K18. Of the two GCN5 paralogues in *T. gondii*, *Tg*GCN5b alone is necessary and sufficient to catalyze this selective tri-site acetylation in tachyzoites, whereas *Tg*GCN5a, contrary to earlier suggestions, neither compensates for nor contributes to H3K18 acetylation^11^. This division of labor between paralogues supports a model of functional specialization in which *Tg*GCN5b evolved as the dominant euchromatic HAT. Its ability to acetylate H3K18 resembles the broader substrate specificity reported for GCN5-containing complexes in metazoans and plants rather than the more restricted H3K9/H3K14 activity characteristic of fungal GCN5.

Consistent with this expanded catalytic potential, and given that lysine acetyltransferases typically act on a wide range of substrates beyond histone tails, we used an unbiased proteome-wide acetylome analysis to define the broader *Tg*GCN5b-dependent acetylation landscape. Depletion of *Tg*GCN5b caused a widespread reduction in acetyl-lysine (AcK) site occupancy across the parasite proteome (Supplementary data 2). Using significance thresholds of log2(fold change ≥ 1) and *p* < 0.01 leading to a Benjamini–Hochberg estimated false discovery rate <1%, we identified 149 acetylation sites enriched in untreated parasites relative to *Tg*GCN5b-depleted parasites (Supplementary Fig. 2a and Supplementary data 2). Comparison with the corresponding total proteome measurements indicated that most of these changes could not be explained by altered protein abundance, yielding 135 candidate *Tg*GCN5b-dependent acetylation sites across 99 proteins. Although these proteins belonged to diverse functional classes, Gene Ontology (GO) analysis revealed a strong enrichment for nuclear and chromatin-related functions, including DNA binding, histone acetylation, transcription initiation, and transcription factor activity (Supplementary Fig. 2b). Collectively, these data define a predominantly nuclear, chromatin- and transcription-associated *Tg*GCN5b acetylome.

Notably, *Tg*GCN5b regulates the acetylation levels of a far broader range of substrates than previously appreciated. In addition to its established activity on H3K9–K14–K18, *Tg*GCN5b emerged as a major regulator of the histone variant H2B.Z. Acetylation of H2B.Z at K4, K9, K14, and K15, either individually or in multiple combinatorial states, was strongly dependent on *Tg*GCN5b, suggesting H2B.Z as a major substrate of this acetyltransferase (Fig. 4c, f). Beyond histones, *Tg*GCN5b-dependent acetylation was detected on numerous proteins involved in transcriptional regulation and chromatin organization (Supplementary data 2). These included several components of the *Tg*GCN5b regulatory network, notably the SAGA-associated factors (Fig. 4d). The abundances of *Tg*ADA2-A and *Tg*PHD1 proteins were reduced following *Tg*GCN5b depletion, indicating that the loss of acetylation observed on these proteins likely reflects, at least in part, reduced protein abundance (Supplementary Fig. 2c). In contrast, several other SAGA subunits displayed decreased acetylation at *Tg*GCN5b-dependent sites without detectable changes in protein levels (Fig. 4d and Supplementary Fig. 2c). Given their intimate association with *Tg*GCN5b within the SAGA complex, these modifications may represent proximity-driven acetylation events arising from local exposure to *Tg*GCN5b catalytic activity rather than dedicated regulatory modifications. Such acetylation may therefore be a consequence of SAGA architecture itself. Several acetylated lysines were also identified on *Tg*GCN5b (Fig. 4d, e). Whether these modifications result from autoacetylation^27^ or from the activity of another lysine acetyltransferase remains unresolved. Collectively, these findings position *Tg*GCN5b as a central regulator of the nuclear acetylome, controlling not only canonical histone marks but also histone variants, transcription factors, and chromatin-associated regulatory proteins.

### *Tg*GCN5b KD disrupts the expression of both host-responsive and housekeeping genes

To determine the contribution of *Tg*GCN5b to transcriptional regulation, we performed RNA-seq analysis following its inducible depletion. To place these effects in the context of the SAGA complex, we analyzed in parallel the consequences of depleting *Tg*PHD1, a core SAGA-associated PHD-domain protein (Supplementary Fig. 1a). Although *Tg*PHD1 is an integral component of the complex, its depletion produced a substantially milder phenotype in cell culture, reducing plaque size without preventing parasite propagation, whereas *Tg*GCN5b depletion completely abolished plaque formation (Fig. 3b, c). This contrast provided an opportunity to distinguish the specific transcriptional consequences associated with loss of the catalytic acetyltransferase from those resulting from disruption of a non-catalytic core component of the SAGA complex.

**Fig. 5:**
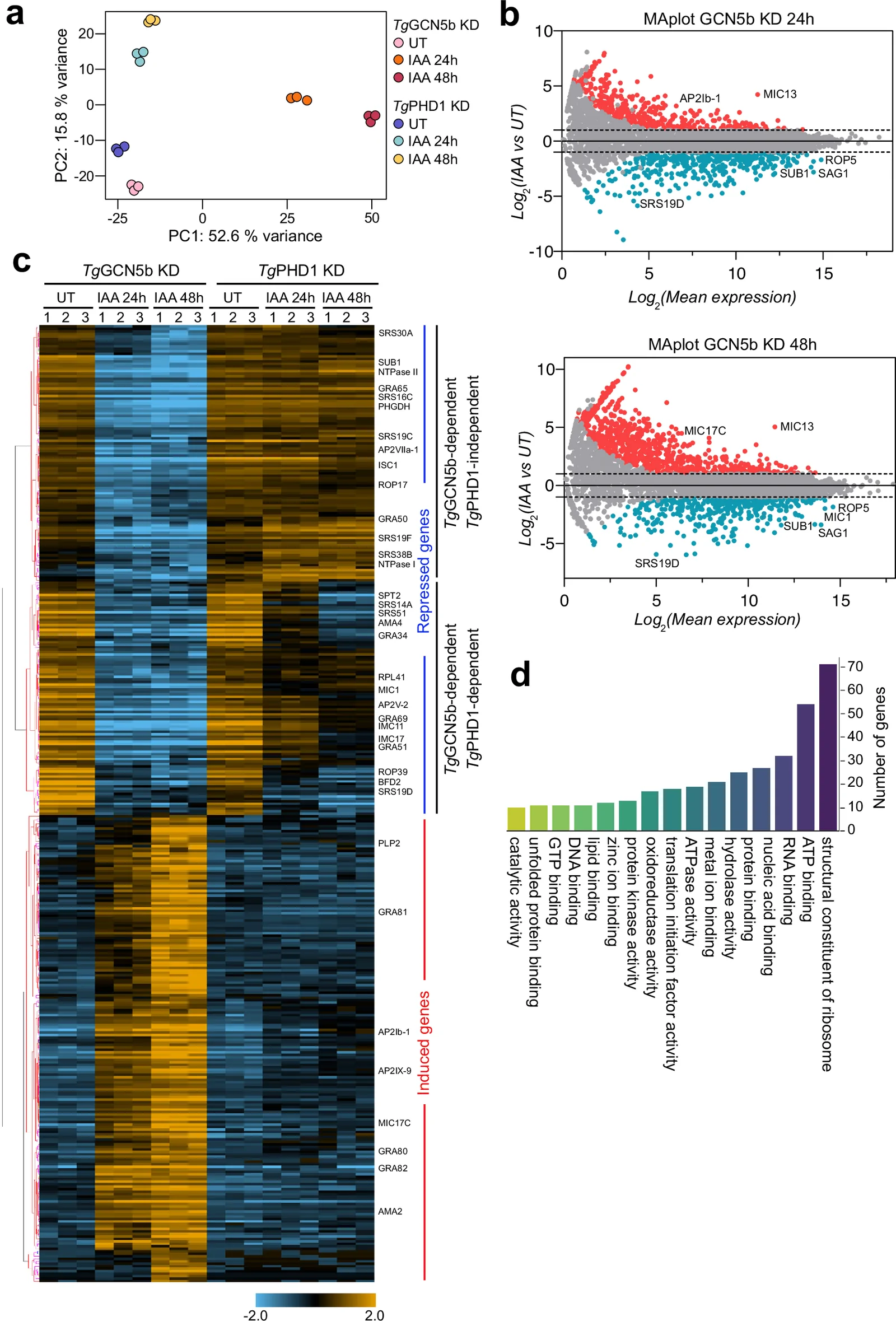
*Tg*GCN5b drives major transcriptomic rewiring, whereas *Tg*PHD1 induces only modest changes. **a** Principal component analysis (PCA) of RNA-seq data from biological triplicates of *Tg*GCN5b-mAID-HA and *Tg*PHD1-mAID-HA parasites, either untreated (UT) or treated with IAA for 24 or 48 h. PCA performed with DESeq2 shows that *Tg*GCN5b depletion strongly separates samples along PC1, whereas *Tg*PHD1 depletion contributes modestly to variance along PC2. **b** MA plots illustrate transcriptome-wide log₂ fold changes relative to log₂ mean expression following *Tg*GCN5b knockdown at 24 h (upper panel) and 48 h (lower panel). Differential expression was assessed using two-sided Wald tests implemented in DESeq2, with *p* values adjusted for multiple comparisons using the Benjamini–Hochberg procedure. Genes significantly upregulated (log₂FC > 1, *P* < 0.05) are highlighted in red, and significantly downregulated genes (log₂FC < −0.58, *P* < 0.05) in blue. Key virulence factors, effectors, and AP2 transcription factors are labeled. **c** Heatmap generated with iDEP.96 using mean-**c**entered, log₂-transformed TPM values and k-means clustering (Pearson correlation). The expression profiles reveal coordinated transcriptional responses in *Tg*GCN5b KD and *Tg*PHD1 KD parasites, with log₂ fold changes indicating upregulated (orange) and downregulated (blue) clusters. **d** Gene ontology (GO) enrichment analysis of *Tg*GCN5b- and *Tg*PHD1-regulated genes. Enriched categories are indicated. Bars represent the number of genes associated with each significantly enriched GO category.

To define the transcriptional consequences of each depletion, we performed Illumina RNA sequencing on bulk RNA isolated from untreated parasites and from parasites subjected to auxin-induced KD (Supplementary data 3). Principal component analysis showed a markedly greater displacement for *Tg*GCN5b KD samples from untreated controls than observed for *Tg*PHD1 KD, with *Tg*PHD1-depleted parasites clustering closer to wild-type samples (Fig. 5a). These results indicate that *Tg*GCN5b plays a dominant role in maintaining the global transcriptional landscape, whereas *Tg*PHD1 contributes to a more restricted subset of transcriptional programs. MA-plot analysis uncovered a marked asymmetry in the transcriptional response, characterized by extensive repression of highly expressed genes (blue) and comparatively limited induction of low-amplitude transcripts (red) (Fig. 5b). This global shift toward transcriptional repression intensified over time and was considerably more pronounced following *Tg*GCN5b depletion than *Tg*PHD1 depletion, highlighting the dominant contribution of *Tg*GCN5b catalytic activity to gene expression maintenance (Supplementary data 3).

DESeq2 analysis of RNA-seq data identified 593 transcripts differentially expressed following *Tg*GCN5b depletion (FC ≥ 2, *p* < 0.05; Supplementary data 3). Among these, 443 genes were downregulated, predominantly highly expressed tachyzoite transcripts, while 151 merozoite-restricted genes displayed partial induction. The observed off-context upregulation of merozoite genes parallels the transcriptional signature described following AP2XII-1/AP2XI-2 co-depletion^28^, though its magnitude is considerably lower and it occurs without the associated protein expression characteristic of stage conversion (data not shown). In contrast to *Tg*GCN5b depletion, *Tg*PHD1 knockdown altered only 48 transcripts, causing modest repression of tachyzoite housekeeping genes and no detectable off-context induction, underscoring its limited contribution to global transcriptional control (Fig. 5a, c). Hierarchical clustering analysis revealed that *Tg*GCN5b depletion preferentially repressed highly expressed tachyzoite genes (Fig. 5c), including housekeeping genes involved in translation and RNA processing. Consistent with this, Gene Ontology analysis highlighted an enrichment of ribosome- and translation-related terms among downregulated genes, whereas broader nucleic acid- and protein-binding categories characterized effector and regulatory modules (Fig. 5d). *Tg*GCN5b-depleted zoites also displayed marked downregulation of secretory organelle–associated proteins (MICs, ROPs, GRAs, SRSs) critical for invasion, parasitophorous vacuole maintenance, and host–parasite interactions, as well as components of the Inner Membrane Complex (IMC). Loss of these effectors provides a mechanistic explanation for the early lytic-cycle arrest observed upon TgGCN5b depletion. A smaller subset of genes was co-repressed in a time-dependent manner by *Tg*GCN5b and *Tg*PHD1 knockdowns (Fig. 5c). Together, these results identify *Tg*GCN5b as a central transcriptional driver that sustains core housekeeping expression while fine-tuning effector gene programs essential for the parasitic lifestyle of *T. gondii*, whereas its partner *Tg*PHD1 functions mainly as a chromatin-tethering scaffold that likely modulates, but does not reprogram, transcriptional output.

### *Tg*GCN5b, not *Tg*PHD1, drives chromatin engagement of the HAT module

**Fig. 6:**
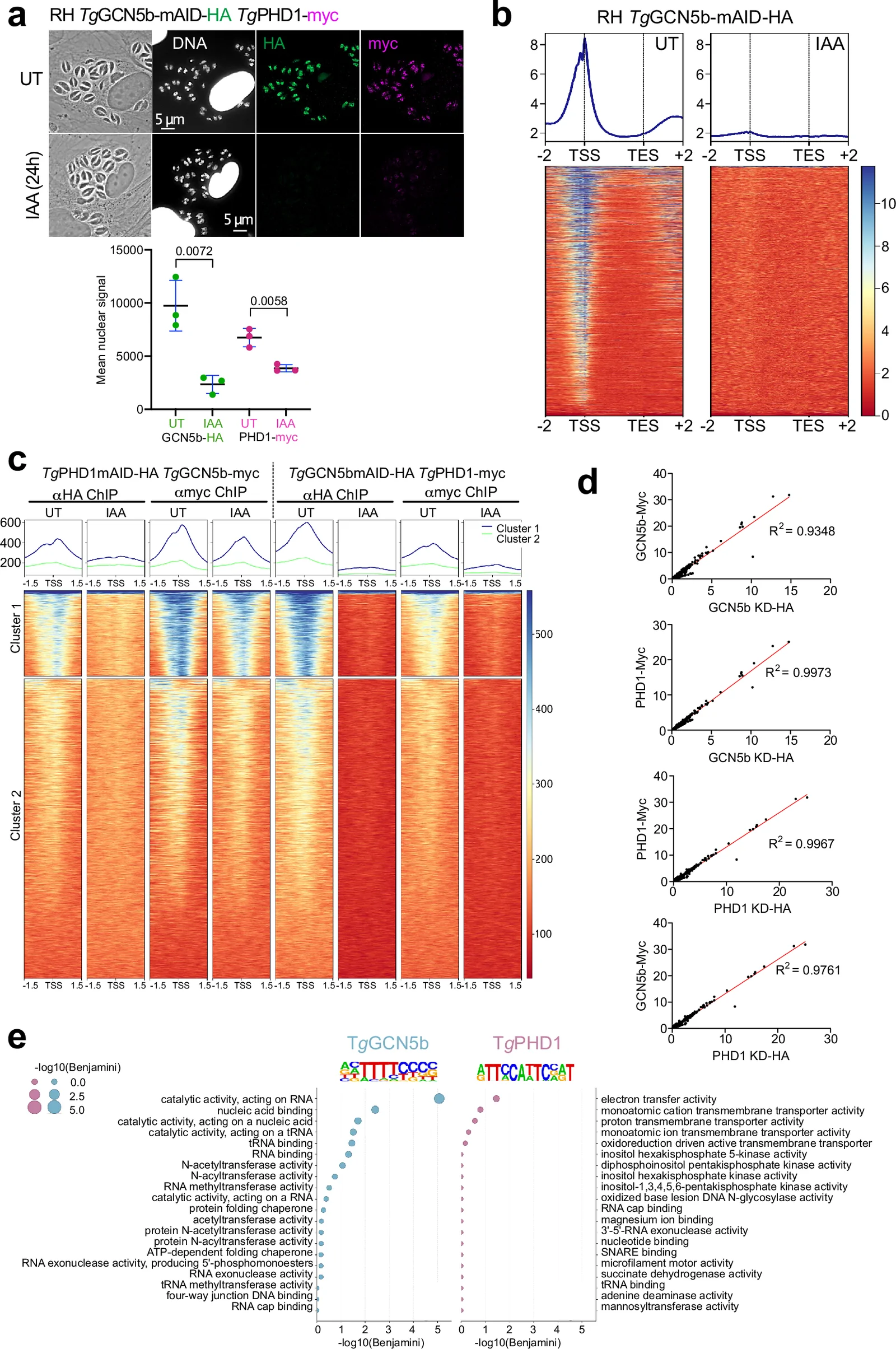
*Tg*GCN5b and *Tg*PHD1 display highly correlated chromatin occupancy with distinct motifs and functional enrichments. **a** Immunofluorescence analysis of *Tg*GCN5b-mAID-HA parasites expressing *Tg*PHD1-Myc in the absence (UT) or presence of IAA for 24 h. Parasites were stained with antibodies against HA and Myc to detect *Tg*GCN5b and *Tg*PHD1, respectively. Nuclear HA and Myc fluorescence signals were quantified from three independent experiments (*n* = 100 nuclei per condition per experiment). Data are presented as mean ± s.d. Statistical significance was determined by one-way ANOVA with Tukey’s multiple-comparison test. Scale bars, 5 μm. **b**, Genome-wide distribution of *Tg*GCN5b occupancy determined by ChIP–seq in untreated parasites and following TgGCN5b depletion. Average normalized ChIP–seq signal is shown across gene bodies and ±2 kb surrounding transcription start sites (TSS) and transcription end sites (TES) (top). Corresponding heatmaps of individual loci are shown below. *Tg*GCN5b is predominantly enriched at promoter regions centered on TSSs, with lower occupancy across gene bodies and a secondary enrichment downstream of TESs. *Tg*GCN5b occupancy is strongly reduced following IAA treatment. **c** Heatmaps and corresponding average profiles display ChIP-seq peak intensities for *Tg*GCN5b and *Tg*PHD1 in untreated parasites and following reciprocal depletion (*Tg*PHD1 KD or *Tg*GCN5b KD). K-means clustering performed on untreated samples identifies two major gene clusters, with Cluster 1 showing pronounced enrichment of both factors at transcription start sites (TSS), indicating promoter-centered binding. **d** Scatterplots comparing ChIP–seq signal intensities of *Tg*GCN5b and *Tg*PHD1 in reciprocal tagged strains. Pearson correlation coefficients (*R*²) are indicated. **e** De novo motif discovery and Gene Ontology (GO) molecular function enrichment analyses of TgGCN5b- and TgPHD1-bound loci. Sequence logos show the top-ranked motifs identified within TgGCN5b (SGGGAAAANT; 57.7% of peaks) and TgPHD1 (ATTCCATTCVAT; 1.45% of peaks) ChIP–seq peaks. GO enrichment analysis was performed on genes associated with motif-containing peaks using one-sided hypergeometric tests. Dot size and color represent enrichment significance, expressed as −log10(Benjamini–Hochberg-adjusted *P* value). Source data are provided as a Source Data file.

To test whether *Tg*PHD1 contributes to *Tg*GCN5b chromatin binding, we generated reciprocal parasite strains in which one factor was endogenously tagged while the other was conditionally depleted, and used ChIP-seq to map genome-wide occupancy. Auxin-induced depletion of *Tg*GCN5b caused a marked loss of the *Tg*GCN5b-HA nuclear signal and a concomitant reduction in TgPHD1-myc abundance by IFA after 24 h (Fig. 6a). In contrast, *Tg*PHD1 depletion did not detectably alter *Tg*GCN5b levels. Quantitative proteomics further supported this dependency, confirming reduced *Tg*PHD1 abundance and revealing a parallel decrease in *Tg*ADA2-A upon *Tg*GCN5b depletion (Supplementary Fig. 2c). Thus, *Tg*GCN5b is required not only for HAT catalytic activity but also for the stability of multiple HAT module components. ChIP-seq showed that, in untreated parasites, *Tg*GCN5b and *Tg*PHD1 occupied highly overlapping genomic regions, with enrichment concentrated around transcription start sites across tagging and knockdown backgrounds (Fig. 6b–d and Supplementary Fig. 2d). The strong concordance between *Tg*GCN5b and *Tg*PHD1 profiles, together with their TSS-centered heatmap enrichment, is consistent with a shared role in promoter-associated histone acetylation. Upon *Tg*GCN5b depletion, *Tg*PHD1 chromatin occupancy was abolished, mirroring its reduced cellular abundance (Fig. 6c). Conversely, *Tg*PHD1 knockdown eliminated *Tg*PHD1 binding but left TgGCN5b occupancy largely unchanged (Fig. 6c). These data reveal a unidirectional dependency in which *Tg*PHD1 requires *Tg*GCN5b for both stability and chromatin association, whereas *Tg*GCN5b can remain chromatin-bound independently of the PHD scaffold.

To examine whether these factors are associated with distinct sequence features, we performed de novo motif analysis on summit-centered *Tg*GCN5b and *Tg*PHD1 ChIP-seq peaks using HOMER. *Tg*GCN5b peaks were enriched for the motif ANTTTTCCCCS, detected in 57.7% of target regions compared with 41.5% of background sequences (*p* = 1e-144). *Tg*PHD1 peaks contained a distinct top-ranked motif, ATTCCATTCVAT, present in 1.45% of target regions and absent from background sequences (*p* = 1e-116). Molecular-function GO analysis of genes associated with these motif-enriched regions linked *Tg*PHD1-associated sites to electron transfer, ion/proton transmembrane transport, oxidoreduction-driven transporter activity, and inositol phosphate kinase functions, whereas *Tg*GCN5b-associated sites were enriched for RNA and nucleic-acid binding, RNA-processing activities, N-acetyltransferase/acyltransferase functions, and protein-folding chaperone terms (Fig. 6e). Thus, while *Tg*GCN5b and *Tg*PHD1 largely co-occupy promoter-proximal chromatin, their associated sequence and functional enrichments suggest that each factor may contribute distinct specificity within the shared HAT module landscape.

### *Tg*GCN5b imprints distinct chromatin states on genes key to parasite life

**Fig. 7:**
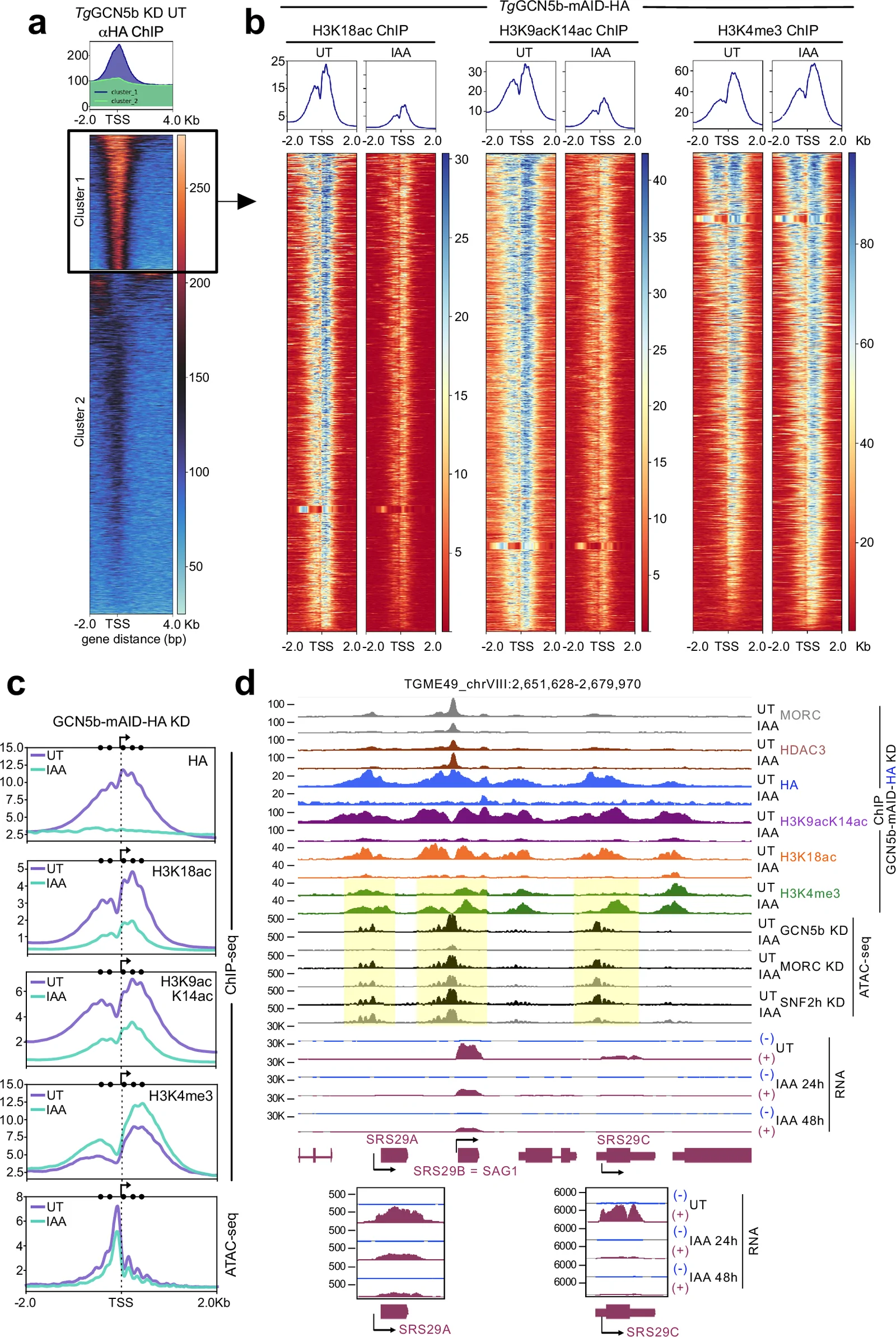
Chromatin accessibility and histone modification changes induced by *Tg*GCN5b depletion. **a** Average ChIP-seq profiles for *Tg*GCN5b-mAID-HA plotted across TSS ± 4 kb in the untreated (UT) condition. *K*-means clustering segregates genes into two major groups. Cluster 1 displays a pronounced promoter-proximal peak, indicating strong *Tg*GCN5b occupancy near the TSS in the UT condition. **b** Heatmaps and averaged profiles of histone mark ChIP-seq signals (H3K9acK14ac, H3K18ac, and H3K4me3) in UT versus *Tg*GCN5b-depleted parasites (IAA). Signals are displayed over TSS ± 2 kb for genes belonging to Cluster 1 (as defined in (**a**)). **c** Average signal profiles for HA, H3K18ac, H3K9acK14ac, H3K4me3 ChIP-seq, and ATAC-seq in *Tg*GCN5b-mAID-HA parasites under untreated (UT) and IAA conditions, plotted across TSS ± 2 kb. Each track displays the normalized enrichment patterns for the indicated mark or assay, with UT and IAA conditions overlaid for comparison. Black dots indicate representative nucleosome positions inferred from ATAC-seq valleys between adjacent accessibility peaks in the untreated condition. **d** Genome browser view of the SRS29B/SAG1 locus displaying ATAC-seq, ChIP-seq, and RNA-seq tracks in untreated (UT) and IAA-treated *Tg*GCN5b-mAID-HA parasites. Tracks include HA, H3K9acK14ac, H3K18ac, H3K4me3, MORC, and HDAC3 ChIP-seq, together with ATAC-seq and RNA-seq coverage at 24 and 48 h. Additional ATAC-seq profiles from MORC KD and *Tg*SNF2h KD are shown for comparison. The yellow box highlights a highly open chromatin site whose accessibility is sensitive to *Tg*GCN5b loss. At the bottom, strand-specific RNA-seq tracks for SRS29A, SRS29B/SAG1 and SRS29C illustrate coordinated changes in transcript levels across the SRS29/SAG1 gene superfamily over time following *Tg*GCN5b loss. (+), forward strand; (−), reverse strand.

As expected, *Tg*GCN5b binds preferentially to chromatin enriched for its hallmark acetylation marks, H3K18ac and H3K9ac/K14ac. When the enzyme is depleted, these same regions undergo a selective and pronounced loss of acetylation (cluster 1, Fig. 7a, b), confirming that these PTMs depend directly on *Tg*GCN5b’s catalytic activity. This transition from an acetylation-rich to acetylation-poor state establishes a mechanistic link between *Tg*GCN5b activity and local chromatin accessibility. To determine whether *Tg*GCN5b chromatin binding is associated with gene activation, we integrated *Tg*GCN5b ChIP-seq, ATAC-seq, and RNA-seq datasets. This analysis revealed that promoter accessibility positively correlates with transcript abundance (Supplementary Fig. 2e; Spearman’s rho = 0.48, *p* ≈ 0), supporting the view that open *Tg*GCN5b-associated chromatin marks transcriptionally active genes. Importantly, when genes were stratified according to *Tg*GCN5b ChIP-seq signal intensity, increasing *Tg*GCN5b occupancy was accompanied by a progressive increase in RNA expression levels (Supplementary Fig. 2f). Thus, *Tg*GCN5b binding is not merely a passive chromatin feature but is strongly associated with transcriptional output.

**Fig. 8:**
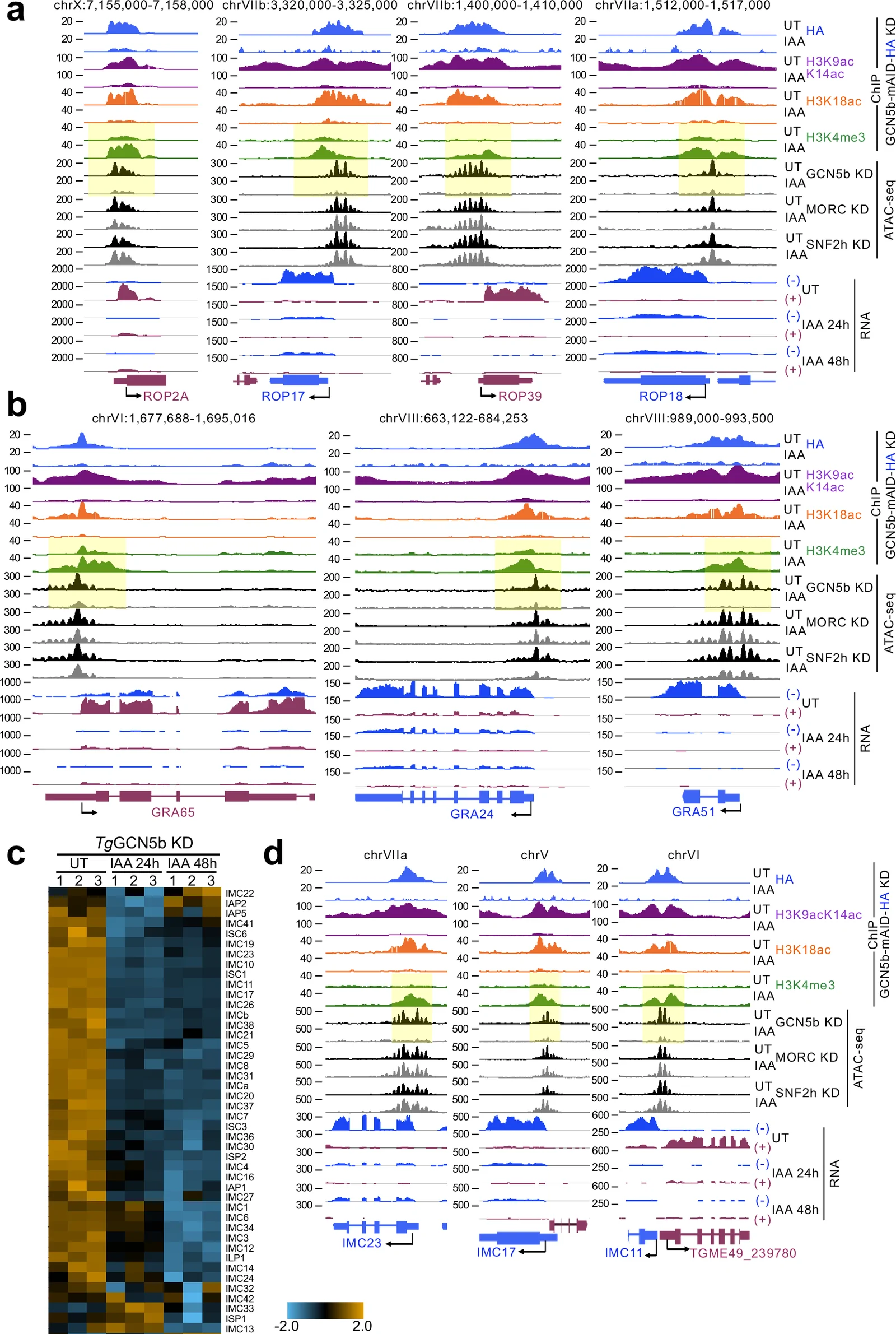
*Tg*GCN5b depletion alters chromatin states and transcription at virulence factor and inner membrane complex genes. Genome browser views of representative virulence-associated loci encoding rhoptry proteins (ROP2A, ROP17, ROP39 and ROP18) (**a**) and dense granule proteins (GRA65, GRA24 and GRA51) (**b**). Tracks show *Tg*GCN5b-HA, H3K9acK14ac, H3K18ac, and H3K4me3 ChIP–seq signals in untreated (UT) and IAA-treated *Tg*GCN5b-mAID-HA parasites, together with matched ATAC-seq and RNA-seq profiles at 24 and 48 h after auxin treatment. ATAC-seq datasets from MORC and *Tg*SNF2h knockdown parasites are shown for comparison. These loci exemplify the chromatin transition highlighted in yellow and observed across Cluster 2 (~300 direct *Tg*GCN5b targets), characterized by loss of histone acetylation and chromatin accessibility together with increased H3K4me3 following *Tg*GCN5b depletion. **c** Heatmap showing expression profiles of inner membrane complex (IMC) genes in *Tg*GCN5b-mAID-HA parasites cultured in the absence or presence of IAA for 24 or 48 h. Values represent log2-transformed, mean-centered RNA-seq data. Genes were grouped by *k*-means clustering using Pearson correlation in iDEP.96. **d** Genome browser views of representative IMC loci (IMC23, IMC17 and IMC11) showing MORC, HDAC3, *Tg*GCN5b-HA, H3K9acK14ac, H3K18ac, and H3K4me3 ChIP–seq profiles together with corresponding ATAC-seq and RNA-seq datasets. ATAC-seq tracks from MORC and *Tg*SNF2h knockdowns are included for comparison. (+), forward strand; (−), reverse strand.

By intersecting *Tg*GCN5b-bound genes (cluster 1) with those downregulated upon depletion (*n* = 443 genes, DESeq2 analysis), we defined a high-confidence set of ~300 direct targets, hereafter referred to as cluster 2 (Supplementary data 3). Across many of these genes, *Tg*GCN5b loss induces a stereotyped chromatin response: histone acetylation declines, *Tn5* chromatin accessibility decreases (lower ATAC-seq signal), and H3K4me3 levels paradoxically increase (Fig. 7b–d and Supplementary Fig. 3a). This uncoupling of acetylation, accessibility and H3K4me3 challenges the classical view of a unified “active chromatin triad” and indicates that H3K4me3 alone is insufficient to sustain productive transcription in the absence of *Tg*GCN5b-dependent acetylation (Fig. 7c, d). These promoters may therefore become trapped in a poised but transcriptionally impaired state, potentially linked to altered RNA polymerase II recruitment or promoter-proximal pausing^29^. This dual-PTM imbalance (yellow boxes in the following figures) is observed across gene families central to *T. gondii* biology, including key virulence factors (ROP5, ROP18, Fig. 8a), host-modulating effectors (GRA65, GRA24, Fig. 8b), surface antigens (SRS29B/SAG1 locus, Fig. 7d), and invasion factors (microneme genes such as SUB1, MIC1, and CLIP; Supplementary Fig. 3a). Together, these data identify *Tg*GCN5b as a major organizer of promoter states that sustain parasite virulence, invasion and host-cell interaction programs. Its depletion drives a coordinated transition from an acetylation-rich, ATAC-open and transcriptionally active state to an H3K4me3-dominant, less accessible and transcriptionally repressed configuration.

The IMC gene program provides a particularly striking example of this regulatory paradigm. *Tg*GCN5b occupies multiple IMC-associated loci, including IMC17, IMC23, and IMC11, where its depletion results in the loss of H3K9ac/K14ac and H3K18ac, reduced chromatin accessibility, and transcriptional repression despite persistent H3K4me3 marking (Fig. 8c, d). Consistent with this molecular collapse, *Tg*GCN5b-deficient parasites exhibit profound defects in IMC biogenesis, including fragmented IMC structures, incomplete septation, and the formation of multiple daughter buds within a single mother parasite, as revealed by IMC1, IMC7, and GAP45 staining (Fig. 3d–f). These observations directly connect *Tg*GCN5b-dependent promoter acetylation to the maintenance of the IMC transcriptional program and provide a mechanistic explanation for how chromatin rewiring culminates in defective daughter-cell assembly, cytokinesis, and organelle inheritance.

**Fig. 9:**
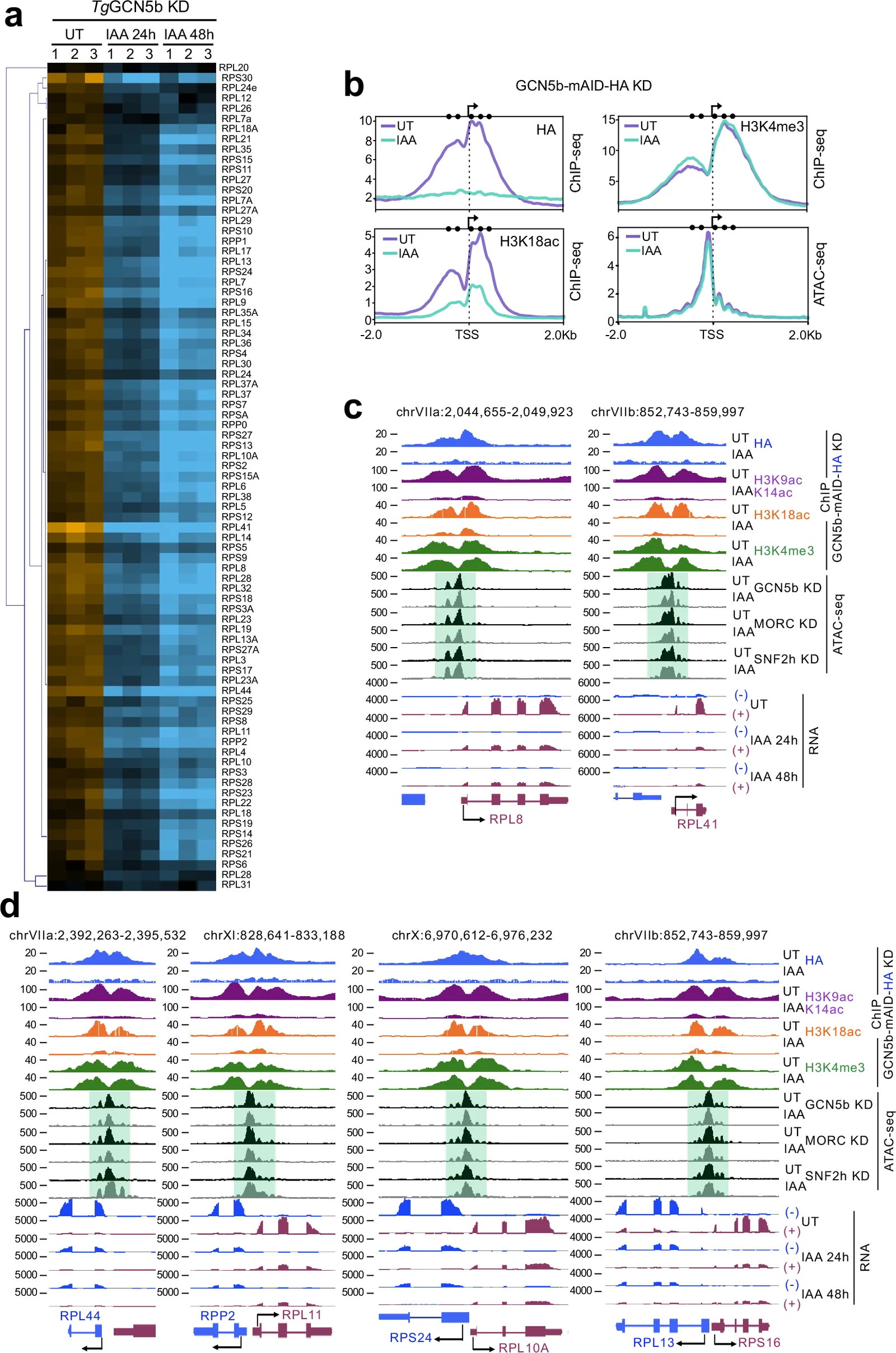
Ribosomal loci collapse transcriptionally despite preserved H3K4me3 and chromatin opening upon *Tg*GCN5b depletion. **a** Heatmap of ribosomal protein genes (RPGs) showing transcript levels in *Tg*GCN5b-mAID-HA parasites left untreated (UT) or treated with IAA for 24 and 48 h. Most RPGs exhibit a progressive loss of expression following *Tg*GCN5b depletion. The heatmap uses mean-centered data and k-means clustering (Pearson correlation) using iDEP.96, and shows log2-transformed data. **b** Average ChIP-seq and ATAC-seq profiles for HA, H3K18ac, H3K4me3 and chromatin accessibility (TSS ± 2 kb) in UT and IAA-treated parasites. *Tg*GCN5b depletion leads to a marked reduction in H3 acetylation, while H3K4me3 peaks remain largely preserved and chromatin is unaffected. Black dots indicate representative nucleosome positions inferred from ATAC-seq valleys between adjacent accessibility peaks in the untreated condition. **c**, **d** Genome browser views of representative *RPG* loci, displaying ChIP-seq profiles for *Tg*GCN5b-HA, H3K9acK14ac, H3K18ac, and H3K4me3 in UT and IAA-treated parasites, together with matched ATAC-seq and RNA-seq coverage at 24 and 48 h. ATAC-seq tracks from MORC and *Tg*SNF2h knockdowns are included for comparison. Green boxes highlight regions where *RPG* promoters show co-enrichment of H3 acetylation and H3K4me3 under UT conditions, indicative of an open, transcriptionally active state. Upon *Tg*GCN5b depletion, acetylation collapses while H3K4me3 remains and transcription drops sharply, revealing a poised but largely inactive chromatin configuration. (+), forward strand; (−), reverse strand.

Yet, not all cluster 2 genes respond identically. Ribosomal protein genes (RPGs), whose expression drops drastically upon *Tg*GCN5b depletion, are particularly informative (Fig. 9a). In untreated parasites, RPG loci co-display high H3 acetylation, H3K4me3, and accessible chromatin, consistent with a highly active transcriptional configuration (green boxes in the figures) (Fig. 9b–d). After *Tg*GCN5b depletion, acetylation collapses while H3K4me3 persists and chromatin remains relatively open; RPG transcription falls to near-background levels, predicting a major translational bottleneck. This uncoupling suggests that, at RPG loci, chromatin accessibility and H3K4me3 can be maintained in a poised-like state but are insufficient to sustain productive transcription without *Tg*GCN5b-dependent acetylation. Thus, *Tg*GCN5b-driven acetylation, rather than H3K4me3 or accessibility alone, emerges as the decisive chromatin feature linked to transcriptional output at high-demand housekeeping genes. In line with this, reports that GNAT2-family acetyltransferases interact with SET1 orthologs raise the possibility that *Tg*GCN5b activity is functionally coupled to *Tg*SET1-mediated deposition of H3K4me3 (Fig. 2g and Supplementary Fig. 1g).

### *Tg*GCN5b sustains open promoter states that locally support MORC/HDAC3 occupancy

*Tg*GCN5b has long been proposed to act in opposition to HDAC3^12^. Given its broad impact on chromatin accessibility and its ability to shift loci between active, repressed, and poised states, we asked whether *Tg*GCN5b functionally intersects with the MORC/HDAC3 repression axis or with its upstream epistatic regulator, the *Tg*SNF2h remodeler^30^. This question is particularly relevant because MORC/HDAC3 occupies silenced, heterochromatin-like domains, whereas *Tg*GCN5b is enriched at euchromatic promoters. Cross-talk between these pathways would therefore be most likely at chromatin boundaries or bivalent loci. In the compact *T. gondii* genome, where stage-specific genes are interspersed with actively transcribed genes and bidirectional gene pairs often display polarized transcription, local differences in acetylation define domains of distinct accessibility. These features raised the possibility that *Tg*GCN5b activity may overlap with, buffer, or counteract HDAC3-associated regulatory zones. To test this, we integrated ATAC-seq datasets generated before and after MORC or *Tg*SNF2h depletion^30^ with MORC and HDAC3 ChIP-seq profiles obtained in the context of *Tg*GCN5b knockdown.

**Fig. 10:**
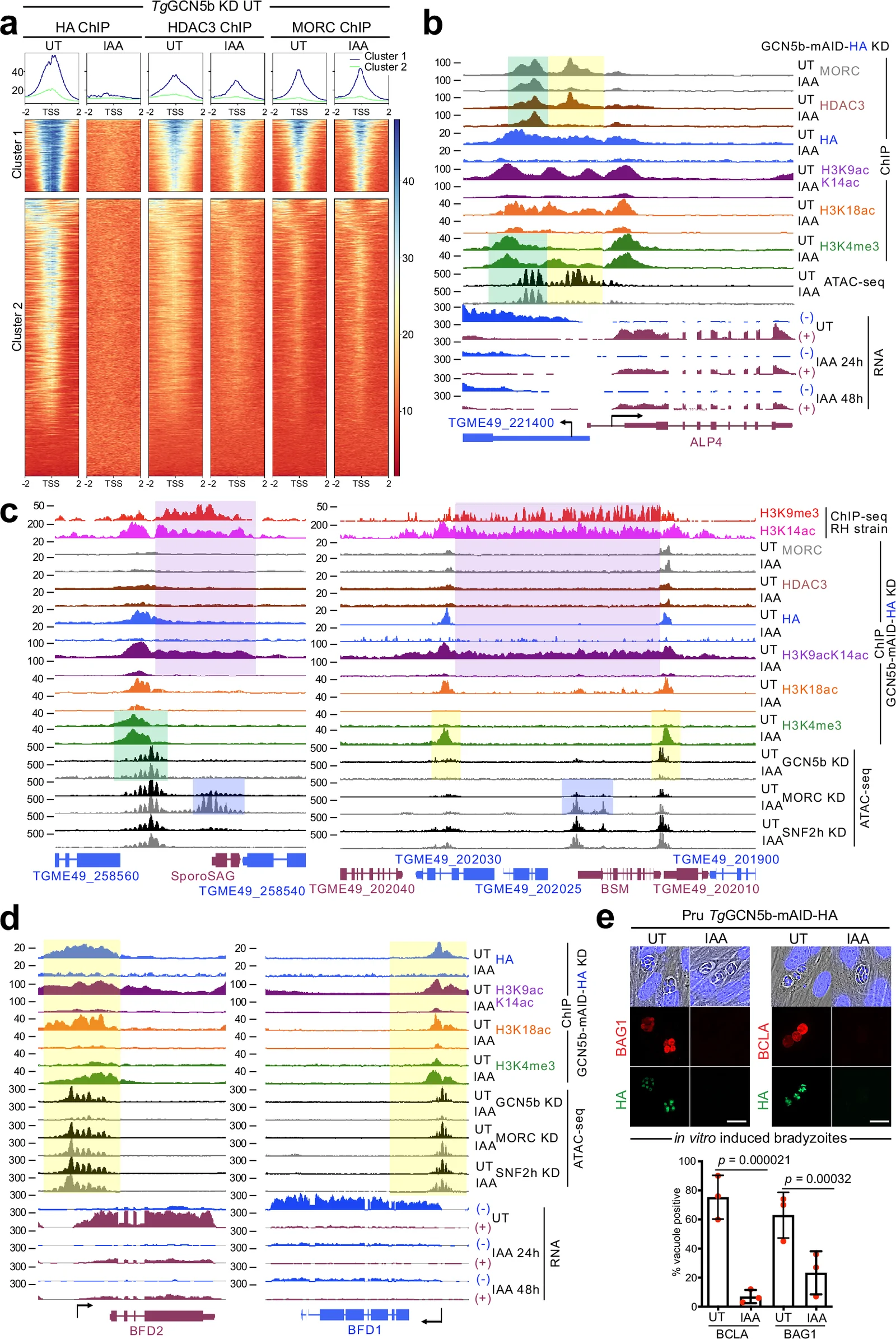
*Tg*GCN5b maintains acetylation-rich chromatin at developmental loci and promotes bradyzoite differentiation. **a** Heatmaps and average profiles showing *Tg*GCN5b (HA), HDAC3, and MORC ChIP–seq signals across transcription start sites (TSS ± 2 kb) in untreated (UT) and IAA-treated *Tg*GCN5b-mAID-HA parasites. *K*-means clustering identified two major groups of loci (Clusters 1 and 2). Corresponding enrichment profiles are shown for each factor before and after *Tg*GCN5b depletion. **b** Genome browser views of representative loci showing ChIP-seq profiles for HA, HDAC3, MORC, H3K9acK14ac, H3K18ac, and H3K4me3 in *Tg*GCN5b-mAID-HA parasites in UT and IAA conditions, along with matched ATAC-seq and RNA-seq coverage at 24 and 48 h. The yellow box highlights a chromatin region with strong accessibility that diminishes upon *Tg*GCN5b depletion, whereas the green box marks a poised chromatin site with more limited sensitivity to *Tg*GCN5b loss. These same-colored regions also illustrate the persistence of MORC and HDAC3 binding, consistent with their preferential association with open or poised chromatin states. **c** Genome browser views of chronic- (*BSM*) and sexual-stage genes (*SporoSAG, PF16*) embedded within bimodal chromatin domains. For each locus, ChIP-seq profiles for repressive (H3K9me3) and activating (H3K14ac, H3K9acK14ac, H3K18ac) histone marks, together with MORC, HDAC3, and *Tg*GCN5b-HA, are shown in untreated (UT) and IAA-treated *Tg*GCN5b-mAID-HA parasites. Corresponding ATAC-seq and RNA-seq coverage at 24 and 48 h, as well as ATAC-seq tracks from MORC and *Tg*SNF2h KDs, are provided for comparison. **d** Genome-browser views of BFD2 and BFD1 showing the datasets described in **c**. Yellow boxes denote acetylation-rich, highly accessible regions sensitive to *Tg*GCN5b loss; green boxes indicate regions retaining H3K4me3 with modest accessibility changes. **e** Immunofluorescence of in vitro-induced bradyzoites derived from Pru *Tg*GCN5b–mAID–HA parasites cultured with or without IAA and stained for HA and the bradyzoite markers BAG1 or BCLA. BAG1- and BCLA-positive vacuoles were quantified. Data are mean ±  s.d. from three independent biological replicates, with 50 vacuoles analyzed per replicate. Statistical significance was assessed using an unpaired two-sided Student’s *t*-test. Scale bars, 5 μm. (+), forward strand; (−), reverse strand. Source data are provided as a Source Data file.

Our data revise a simple opposition model. MORC and HDAC3 are not absent from *Tg*GCN5b-regulated loci; rather, TSS-centered heatmaps and correlation analyses show detectable MORC and HDAC3 occupancy at a subset of *Tg*GCN5b-bound promoters, with partial co-variation between *Tg*GCN5b, MORC, and HDAC3 signals (Fig. 10a and Supplementary Fig. 4a). Thus, these factors can coexist locally on chromatin. At *Tg*GCN5b-sensitive loci, corresponding to the yellow zone, *Tg*GCN5b depletion causes a coordinated loss of H3K9ac/K14ac, H3K18ac, and chromatin accessibility, accompanied by a decrease in MORC and HDAC3 signals (Fig. 10a and Supplementary Fig. 4a). Thus, MORC/HDAC3 occupancy at these loci appears to rely on the open chromatin state maintained by *Tg*GCN5b. This behavior resembles that observed upon *Tg*SNF2h depletion, which similarly reduces MORC association with chromatin^30^. Importantly, because MORC and HDAC3 are reduced rather than increased after *Tg*GCN5b depletion, the resulting transcriptional repression cannot be explained by enhanced MORC/HDAC3 repressive activity. Instead, it reflects the collapse of a *Tg*GCN5b-dependent promoter-activation state. By contrast, in the green zone, MORC and HDAC3 remain associated with chromatin after *Tg*GCN5b depletion (Fig. 10a and Supplementary Fig. 4a). At these loci, it remains difficult to determine whether persistent MORC/HDAC3 contributes directly to transcriptional repression or simply marks a *Tg*GCN5b-insensitive chromatin state.

Collectively, these data overturn a simple activator-versus-repressor model and instead uncover an unexpected dependency of MORC/HDAC3 occupancy on *Tg*GCN5b-maintained chromatin accessibility at a subset of promoters. This finding suggests that *Tg*GCN5b not only promotes transcriptionally competent chromatin states but also contributes to the establishment of local chromatin environments permissive for MORC association. In light of the proposed CTCF-like architectural role of MORC in apicomplexans^31,32^, these observations further raise the possibility that *Tg*GCN5b-dependent promoter accessibility may contribute to higher-order chromatin organization, linking local histone acetylation states to the structural partitioning of the parasite genome.

### A secondary yet functionally relevant contribution of *Tg*GCN5b to stage-specific gene regulation

In a previous study^33^, we observed that several genes specific to the chronic stage (*BSM*) or the sexual cycle (e.g., *SporoSAG and PF16*), normally repressed in tachyzoites, are embedded within a bimodal chromatin domain, a configuration where two opposing epigenetic marks coexist on the same genomic region. In *T. gondii*, this bimodal state is characterized by the simultaneous presence of H3K9me3 (a repressive heterochromatin mark) and H3K14ac (an activating acetylation mark)^33^ (Fig. 10c and Supplementary Fig. 4c). These domains often extend across broad chromosomal regions and are typically insulated by MORC/HDAC3 at their distal boundaries. The presence of H3K14ac within otherwise repressive territories prompted us to examine whether *Tg*GCN5b contributes to the establishment or maintenance of this unusual chromatin signature. Our first observation is that bimodal chromatin is completely devoid of H3K18ac, the hallmark *Tg*GCN5b-dependent acetylation (Fig. 10c and Supplementary Fig. 4c). In contrast, H3K14ac deposition is clearly dependent on *Tg*GCN5b activity. When *Tg*GCN5b is depleted, H3K14ac collapses not only at the nucleation site near the TSS, where *Tg*GCN5b normally binds, but also across the extended heterochromatic regions (Fig. 10c and Supplementary Fig. 4c). Despite this loss of acetylation, these loci do not undergo chromatin opening, unlike what is observed upon MORC knockdown (blue zone, Fig. 10c and Supplementary Fig. 4c). This indicates that although *Tg*GCN5b contributes to the acetylation component of the bimodal state, H3K9me3 remains the dominant determinant of repression within these regions. Taken together, these results support a model in which *Tg*GCN5b participates in shaping the polarity of bimodal chromatin toward a closed configuration near repressed, stage-specific genes, yet its contribution remains secondary to the repressive activity of MORC/HDAC3, which ultimately governs the chromatin state of these loci.

Beyond its local effects on bimodal chromatin states, *Tg*GCN5b also acts upstream of the master regulator of the chronic stage, BFD1, and its co-regulator BFD2 (Fig. 10d). In tachyzoites, BFD2 is basally expressed, while BFD1 is transcribed but remains translationally silent. Upon entry into the bradyzoite program, BFD2 promotes BFD1 translation, and newly synthesized BFD1 feeds back to enhance BFD2 transcription, establishing a self-reinforcing positive-feedback loop^34^. Upon *Tg*GCN5b depletion, both loci lose activating acetylation marks, undergo chromatin compaction, and become transcriptionally repressed (Fig. 10d). This defect has clear developmental consequences: depletion of *Tg*GCN5b in cystogenic type II strain fails to induce the bradyzoite hallmark BAG1 and the cyst-decorating markers BCLA during in vitro differentiation (Fig. 10e). Thus, *Tg*GCN5b acts upstream of the BFD1–BFD2 circuit and is essential for the transcriptional program underlying chronic-stage development.

## Discussion

In most opisthokonts, transcriptional coactivation relies on a single canonical SAGA complex centered on the GCN5–ADA2–ADA3 histone acetyltransferase module. In contrast, plants evolved a dual system: they retain a canonical SAGA, but also assemble a distinct PAGA (plant-ADA2A-GCN5-acetyltransferase) complex^23^ (Fig. 1e). PAGA replaces the SPT–ADA3 structural submodules of SAGA with a multi-PHD scaffold (SPC) that links ADA2A to an H3K4me3 reader such as ING1, generating an ADA3-independent HAT optimized for nucleosomal acetylation^23^. Histone acetylation in *T. gondii* is similarly driven by a *Tg*GCN5b-based mechanism that diverges from opisthokonts and plant canonical SAGA. Proteomics and AlphaFold-based predictions map a *Tg*GCN5b–ADA2A core lacking ADA3, reinforced by a TUDOR-containing SGF29-like H3K4 reader. The complex substitutes the SPT module for PHD/PZP scaffolds. By analogy to plant PAGA, *Tg*GCN5b engages *Tg*PZP1, the closest SPC homolog, that tethers lineage-specific AP2-related transcription factors. These features point to an ADA2A-centered, PAGA-like scaffolded HAT in apicomplexans (Fig. 1e).

Comparison with the recently described *P. falciparum Pf*GCN5 complex supports the view that apicomplexans remodeled ancestral SAGA/PAGA-like machineries in lineage-specific ways. *Pf*GCN5 associates with *Pf*ADA2, *Pf*PHD1, *Pf*PHD2 and the ApiAP2 factor *Pf*AP2-LT, but lacks a recognizable SGF29/Tudor subunit^19^. In this context, *Pf*PHD1 may substitute for SGF29 by reading H3K4me3/2 and anchoring the complex to active chromatin. By contrast, the *T. gondii Tg*GCN5b complex retains a divergent *Tg*SGF29/Tudor-like protein and has expanded its reader repertoire through PHD/PZP factors, including *Tg*PHD1/TgPZP1 and the *Toxoplasma*-specific *Tg*PHD2/TgPZP2. These architectural differences parallel functional divergence. In *P. falciparum*, *Pf*GCN5 or *Pf*PHD1 domain deletions similarly impair growth, invasion, transcription, H3K9ac, and H3K4me3, although *Pf*PHD1 deletion more strongly disrupts complex integrity. In *T. gondii*, *Tg*GCN5b depletion reduces H3K9ac, H3K14ac, and H3K18ac, while H3K4me3 persists or increases. Thus, both parasites use AP2-linked GCN5 complexes, but differ in how H3 acetylation, H3K4me3 recognition, and complex stability are coupled. These differences likely reflect lineage-specific losses and expansions after the divergence of haemosporidian and coccidian apicomplexans, as well as adaptation to distinct life-cycle programs and chromatin regulatory demands.

Notably, the *Tg*GCN5b-associated network includes two GNAT paralogues, an organization unprecedented in opisthokonts or plants. Reverse immunoprecipitation of *Tg*GNAT1 reveals this HAT-containing enzyme as a molecular bridge linking the *Tg*GCN5b–ADA2A–SGF29 core to a minimal HDAC3–MORC module (Fig. 2c), indicative of coordinated acetylation–deacetylation control. Strikingly, and for the first time, none of these core enzymes co-purifies with AP2-related transcription factors, despite their usual promiscuity toward AP2s^13,31^, underscoring a distinct regulatory configuration. Biochemical fractionation further shows that *Tg*GNAT1 readily dissociates from chromatin under low-salt conditions, unlike AP2-associated complexes, supporting a model in which *Tg*GNAT1 acts as a soluble reservoir, redistributing HAT and HDAC modules to fine-tune global acetylation dynamics. In contrast, reverse immunoprecipitation of *Tg*GNAT2 failed to recover *Tg*GCN5b but instead revealed a distinct, HAT-focused network enriched in bromodomain-containing proteins and *Tg*HAT1, delineating a parallel yet interconnected acetylation pathway.

Functionally, *Tg*GCN5b establishes a characteristic H3K9–K14–K18 acetylation pattern that defines promoter accessibility and transcriptional activity. When *Tg*GCN5b is depleted, affected promoters undergo a stereotyped transition: chromatin accessibility drops drastically and transcription collapses, yet H3K4me3 persists, or even rises, despite the loss of acetylation. Thus, H3K4me3 alone cannot sustain expression and instead marks a poised, closed promoter that remembers prior activation but lacks the acetylation required for productive transcription. At other loci (e.g., RPGs), *Tg*GCN5b also drives tri-site acetylation on H3K4me3-positive promoters under basal conditions. Strikingly, upon *Tg*GCN5b loss, acetylation collapses, but both H3K4me3 and chromatin accessibility persist, defining an “open-but-silent” state in which promoter openness is uncoupled from transcription. This is likely constrained by transcription factor engagement rather than chromatin compaction. These two behaviors imply distinct *Tg*GCN5b subcomplexes with different modes of action. Proteomics identifies a *Tg*GCN5b–ADA2A–SGF29 core that partitions with PZP adapters and specific ApiAP2 pairs: *Tg*PZP1 with AP2XII-4/AP2VIIa-5, and *Tg*PZP2 with AP2X-8/AP2IX-7 (Fig. 2c). The two assemblies are likely to target distinct sets of genes and drive different regulatory outcomes. One complex may primarily drive chromatin opening in an acetylation-dependent manner, while the other may promote transcription from already poised chromatin in an acetylation-dependent way. If this division of labor is experimentally confirmed, it would offer a clear mechanism for *Tg*GCN5b-dependent transcriptional plasticity.

Notably, *Tg*GCN5b appears to regulate *RPGs* directly at their promoters in *T. gondii*. This contrasts with the indirect control of *RPGs* observed in budding yeast, where SAGA/GCN5 modulates *RPG* transcription indirectly through acetylation of the activator Ifh1^35–37^. This distinguishes *T. gondii* from the yeast model, in which RPG promoters are typically TFIID-dominated and exhibit little or no SAGA occupancy. The direct binding of *Tg*GCN5b to RPG chromatin implies that the parasite has evolved a streamlined mechanism to couple histone acetylation with ribosome biogenesis, potentially allowing rapid transcriptional reprogramming in response to lytic-cycle demands or during metabolic rewiring. This direct control of RPG promoters also places *Tg*GCN5b in a cell-cycle framework. A recent single-cell study identified AP2XII-8 as a key driver of the G1 transcriptional burst and of a ribosomal protein regulon, with AP2XII-8 depletion impairing G1 progression^38^. In light of our centrosome/budding analysis showing that *Tg*GCN5b-depleted parasites accumulate in a G1-like state (Fig. 3i), the collapse of RPG transcription upon *Tg*GCN5b loss suggests that *Tg*GCN5b and AP2XII-8 may converge on a shared G1 competence program. In this model, AP2XII-8 would provide sequence-specific regulatory input at RPG promoters, whereas *Tg*GCN5b-dependent acetylation would license productive transcription from these chromatin-accessible loci. Whether AP2XII-8 directly recruits *Tg*GCN5b, cooperates with it, or acts through a parallel pathway remains an important question for future work.

Taking a broader perspective beyond promoter-level chromatin remodeling by *Tg*GCN5b, several additional insights emerge from our study. *Tg*GCN5b predominantly operates within euchromatic regions and remains largely segregated from MORC/HDAC3 territories. Their interplay is confined to boundary zones, where *Tg*GCN5b-dependent chromatin openness facilitates MORC/HDAC3 recruitment. Accordingly, loss of MORC or *Tg*SNF2h is unlikely to reactivate *Tg*GCN5b-dependent promoters, whereas inhibition of HAT activity would be expected to reduce MORC/HDAC3 occupancy at domain boundaries. Within bimodal H3K9me3/H3K14ac chromatin domains, *Tg*GCN5b sustains the acetylation arm, specifically H3K14ac, yet the prevailing H3K9me3 mark preserves a closed chromatin configuration. This explains why depletion collapses acetylation polarity without triggering chromatin relaxation. In contrast, MORC depletion profoundly alters chromatin accessibility, leading to the activation of previously repressed genes embedded within these bimodal domains.

From a developmental perspective, *Tg*GCN5b shapes the polarity of bimodal chromatin and safeguards the proper stage-specific expression program. Acting through a distinct mechanism upstream of the BFD1–BFD2 regulatory circuit, *Tg*GCN5b preserves the acetylation landscape necessary for balanced expression of stage regulators. Loss of *Tg*GCN5b results in chromatin compaction and transcriptional silencing of these loci, positioning the enzyme as a gatekeeper of developmental competence rather than a direct executor of cell fate. Although our study focuses on the tachyzoite stage, we propose that *Tg*GCN5b exerts a similar regulatory role across other differentiated forms. Supporting this idea, we have shown that *Tg*GCN5b purified from merozoites induced in vitro by co-depletion of AP2XII-1 and AP2XI-2 retains the same partnership observed in tachyzoites (Supplementary data 2).

In conclusion, *Tg*GCN5b is a central activator of *Toxoplasma gondii* transcription. Its depletion collapses virulence and secretory organelle programs, including ROP5 and ROP18, blocks invasion, disrupts IMC gene expression, and impairs parasite shape, daughter cell formation, and replication. Mechanistically, *Tg*GCN5b defines a reader-rich, ADA2A-centered PAGA-like HAT module that licenses promoter competence through H3 acetylation, especially H3K18ac. Thus, *Tg*GCN5b converts accessible, marked promoters into productive transcriptional units, making its docking surfaces and reader interactions attractive targets to disable parasite virulence and growth.

## Methods

### Parasites and human cell cultures

Human primary fibroblasts (HFFs, ATCC® CCL-171™) were cultured in Dulbecco’s Modified Eagle Medium (DMEM) (Invitrogen) supplemented with 10% heat-inactivated fetal bovine serum (FBS) (Invitrogen), 10 mM (4-(2-hydroxyethyl)-1-piperazine-ethanesulfonic acid) (HEPES) buffer pH 7.2, 2 mM L-glutamine, and 50 μg/ml penicillin and streptomycin (Invitrogen). Cells were incubated at 37 °C and 5% CO_2_. *T. gondii* strains used in this study and listed in Supplementary data 1 were maintained in vitro by serial passage on monolayers of HFFs. Cultures were free of mycoplasma as determined by qualitative PCR.

### Reagents

The following primary antibodies were used in the immunofluorescence, immunoblotting, and/or ChIP assays: rabbit anti-TgHDAC3 (Research Resource Identifier (RRID): AB_2713903), rabbit anti-TgGAP45 (gift from Pr. Dominique Soldati), mouse anti-HA tag (Roche, RRID: AB_2314622), rabbit anti-HA Tag (Cell Signaling Technology, RRID: AB_1549585), rabbit anti-FLAG (Cell Signaling Technology, RRID: AB_2798687) and mouse anti-MYC clone 9B11 (RRID: AB_2148465). Rabbit polyclonal antibodies raised against modified histones: H3K9me3 (Diagenode, RRID: AB_2904605), anti-acetyl-histone H4, pan (Lys5,8,12) (Millipore, RRID: AB_310270), H3K9/14ac (Diagenode, RRID: AB_2637059), H3K4me3 (Diagenode, RRID: AB_2616052), H3K14ac (Diagenode, RRID: AB_2891338), H3K18ac (Diagenode, RRID: AB_2713907), H3K9ac (Diagenode, RRID: AB_2713905), H4K8ac (Diagenode, C15310103). We have also raised homemade antibodies against linear peptides in rabbits corresponding to MORC_Peptide2 (C + SGAPIWTGERGSGA). They were manufactured by Eurogentec and used for immunofluorescence, immunoblotting, and/or chromatin immunoprecipitation. Secondary immunofluorescent antibodies were coupled with Alexa Fluor 488 or Alexa Fluor 594 (Thermo Fisher Scientific). Secondary antibodies used in Western blotting were conjugated to alkaline phosphatase (Promega).

### Auxin-induced degradation

Degradation of POI (protein of interest)-mAID-HA was achieved with 3-indoleacetic acid (IAA, Sigma-Aldrich # 45533). A stock of 500 mM IAA dissolved in 100% EtOH at a ratio of 1:1000 was used to degrade mAID-tagged proteins to a final concentration of 500 μM. The mock treatment consisted of an equivalent volume of 100% EtOH at a final concentration of 0.0789% (wt/vol). To monitor the degradation of mAID-tagged POI, parasites grown in HFF monolayers were treated with auxin or ethanol alone for various time intervals at 37 °C. After treatment, parasites were harvested and analyzed by immunofluorescence or Western blotting.

### Bradyzoite differentiation assay

Differentiation of tachyzoites into bradyzoites was induced by CO₂ depletion at physiological pH (7.4) using HFF as host cells. HFF monolayers were maintained in CO₂-independent-medium (Gibco, Cat# 18045-088) supplemented with 5% FBS, 2mM L-glutamine, and 50 μg/mL penicillin–streptomycin (Invitrogen). Infected cultures were incubated at 37 °C under ambient CO₂ conditions to promote spontaneous differentiation.

### Immunofluorescence microscopy

*T. gondii*-infected HFF cells grown on coverslips were fixed in 3% formaldehyde for 20 min at room temperature, permeabilized with 0.1% (v/v) Triton X-100 for 15 minutes, and blocked in phosphate-buffered saline (PBS) containing 3% (w/v) BSA. Cells were then incubated with primary antibodies for 1 h, followed by the addition of secondary antibodies conjugated to Alexa Fluor 488 or 594 (Molecular Probes). Nuclei were stained with Hoechst 33258 (2 μg/ml in PBS) for 10 min at room temperature. After washing four times in PBS, coverslips were mounted on a glass slide with Mowiol mounting medium, and images were acquired with a fluorescence microscope ZEISS ApoTome.2 and processed with ZEN software (Carl Zeiss, Inc.).

### Western blot

Immunoblot analysis of protein was performed as described^28,30,31^. Briefly, ~10^7^ cells were lysed and sonicated in 50 μl lysis buffer (10 mM Tris-HCl, pH 6.8, 0.5% SDS [v/v], 10% glycerol [v/v], 1 mM EDTA, and protease inhibitor cocktail). Proteins were separated using SDS-PAGE, transferred by liquid transfer to a polyvinylidene fluoride membrane (Immobilon-P; EMD Millipore), and Western blots probed with the appropriate primary antibodies and alkaline phosphatase- or horseradish peroxidase-conjugated secondary goat antibodies. Signals were detected using NBT-BCIP (Amresco) or an enhanced chemiluminescence system (Thermo Scientific).

### Plasmid construction

The plasmids and primers used in this work for the gene of interest (GOI) are listed in Supplementary data 1. To construct the vector pLIC-GOI-HAFlag and pLIC-GOI-mAID- HA or pLIC-GOI-(MYC)2, the coding sequence of GOI was amplified with primers LIC-GOI -Fwd and LIC-GOI -Rev using genomic *T. gondii* DNA as a template. The resulting PCR product was cloned into the vectors pLIC-HF-dhfr or pLIC-mCherry-dhfr using the ligation-independent cloning (LIC) method.

### *T. gondii* transfection

Parasite strains were electroporated with vectors in Cytomix buffer (120 mM KCl, 0.15 mM CaCl_2_, 10 mM K_2_HPO_4_/KH_2_PO_4_, pH 7.6, 25 mM HEPES, pH 7.6, 2 mM EGTA, 5 mM MgCl_2_) using a BTX ECM 630 machine (Harvard Apparatus). Electroporation was performed in a 2 mm cuvette at 1,100 V, 25 Ω, and 25 µF. Antibiotics (concentrations) used were chloramphenicol (20 µM), mycophenolic acid (25 µg/ml) with xanthine (50 µg/ml), pyrimethamine (3 µM), or 5-fluorodeoxyuracil (10 µM) as needed. Stable transgenic parasites were selected with the appropriate antibiotic, cloned in 96-well plates by limiting dilution, and verified by immunofluorescence assay or genomic analysis.

### Chromatographic purification of FLAG-tagged proteins

*T. gondii* extracts from RH∆*ku80* cells stably expressing HAFlag-tagged POI were incubated with anti-FLAG M2 affinity gel (Sigma-Aldrich) for 1 h at 4 °C. Beads were washed with 10-column volumes of BC500 buffer (20 mM Tris-HCl, pH 8.0, 500 mM KCl, 20% glycerol, 1 mM EDTA, 1 mM DTT, 0.5% NP-40, and protease inhibitors). Bound polypeptides were eluted stepwise with 250 μg/ml FLAG peptide (Sigma-Aldrich) diluted in BC100 buffer. For *Tg*GCN5b-HAFlag size-exclusion chromatography, protein eluates from the FLAG IP were loaded onto a Superose 6 HR 10/30 column equilibrated with BC500. The flow rate was set at 0.35 ml/min, and 0.5-ml fractions were collected.

### MS-based quantitative proteomic analyses

#### Analysis of TgGCN5b, TgPHD1, TgPZP1, TgGNAT1, and TgGNAT2 interactomes

Eluted proteins were solubilized in Laemmli buffer and stacked at the top of a 4–12% NuPAGE gel (Invitrogen) before staining with Coomassie blue R-250 (Bio-Rad). Proteins were then in-gel digested using modified trypsin (Promega, sequencing grade) as previously described^39^, except that Tris(2-carboxyethyl)phosphine hydrochloride was used instead of dithiothreitol. The resulting peptides were analyzed by online nanoliquid chromatography (Ultimate 3000 RSLCnano, Thermo Fisher Scientific) coupled to MS/MS (Orbitrap Exploris 480 equipped with a FAIMS Pro device, Thermo Fisher Scientific, for *Tg*GCN5b, *Tg*PHD1, mock, *Tg*GNAT2, and *Tg*PZP1, and Orbitrap Ascend, Thermo Fisher Scientific, for *Tg*GNAT1) operated in data-dependent acquisition mode. For this, peptides were sampled on a 300 μm × 5 mm PepMap C18 precolumn and separated in a 75 μm × 250 mm C18 column (Aurora Generation 3, 1.7 µm, IonOpticks) using a 180 min (for *Tg*GCN5b, *Tg*PHD1, mock, and *Tg*PZP1) or a 120 min (for *Tg*GNAT1 and *Tg*GNAT2) acetonitrile gradient. MS and MS/MS data were acquired using the Xcalibur software (Thermo Fisher Scientific). Peptides and proteins were identified by Mascot (version 2.8.3 or 3.1.0, Matrix Science) through concomitant searches against the *Toxoplasma gondii* database (ME49 taxonomy, downloaded from ToxoDB), the Uniprot database (*Homo sapiens* taxonomy), and a homemade database containing the sequences of classical contaminant proteins found in proteomic analyses (human keratins, trypsin…). Trypsin/P was chosen as the enzyme, and two missed cleavages were allowed. Precursor and fragment mass error tolerances were set at 10 and 20 ppm, respectively. Peptide modifications allowed during the search were: Carbamidomethyl (C, fixed), Acetyl (Protein N-term, variable), and Oxidation (M, variable). The Proline software^40^ was used for the compilation, grouping, and filtering of the results (conservation of rank 1 peptides, peptide length ≥6 amino acids, false discovery rate of peptide-spectrum-match identifications <1%, and a minimum of one specific peptide per identified protein group)^41^. Proline was then used to perform an MS1 label-free quantification of the identified protein groups based on razor and specific peptides. For each protein group, intensity-based absolute quantification (iBAQ)^42^ values were calculated. For characterization of *Tg*GCN5b, *Tg*PHD1, and *Tg*GNAT2 interactomes, statistical analyses were performed using Prostar^43^. Proteins identified in the human and contaminant databases, and proteins identified by MS/MS in less than two replicates of one condition and quantified in less than three replicates of one condition, were discarded. After log2 transformation, abundance values were normalized using the variance stabilizing normalization (vsn) method, before missing value imputation (SLSA algorithm for partially observed values in the condition and DetQuantile algorithm for totally absent values in the condition). Statistical testing was conducted with limma, whereby differentially expressed proteins were selected using a log_2_(Fold Change) cut-off of 4 and a *p*-value cut-off of 0.0001, allowing to reach a false discovery rate inferior to 0.1% according to the Benjamini–Hochberg estimator. Proteins found differentially abundant but identified by MS/MS in less than two replicates, and detected in less than three replicates, in the condition in which they were found to be more abundant, were invalidated (*p*-value = 1).

#### Analysis of TgGCN5b-dependent acetylome and proteome

For each replicate, 2 mg of proteins in 8 M urea-containing buffer were incubated for 1 h at 37 °C with 20 mM of dithiothreitol before alkylation with 55 mM of iodoacetamide for 45 min at room temperature in the dark. The samples were then diluted using ammonium bicarbonate to obtain a urea concentration of 4 M. Proteins were digested with modified trypsin (sequencing grade, Promega) at a ratio of 1:100 for 4 h at 37 °C. The samples were diluted again using ammonium bicarbonate to obtain a urea concentration of 1 M. Proteins were then digested again with modified trypsin at a ratio of 1:100 overnight at 37 °C. The resulting peptides were purified by reverse-phase chromatography (Oasis HLB, Waters) before drying down.

One µg of peptides was used for MS-based quantitative analysis of total proteomes, and 1 mg for enrichment of acetylated peptides using the PTMScan HS Acetyl-Lysine Motif [Ac-K] Kit (Cell Signaling Technology). Peptides obtained after immuno-enrichment were desalted (BioSPE PurePep, Affinisep). The samples were analyzed by online nanoliquid chromatography coupled to mass spectrometry (Ultimate 3000 RSLCnano and Orbitrap Exploris 480, Thermo Fisher Scientific, for total proteome and Vanquish Neo, Thermo Fisher Scientific, and Orbitrap Ascend Tribrid, Thermo Fisher Scientific, for acetylome) operating in data-independent acquisition mode (total proteome) or data-independent acquisition mode (acetylome). Peptides were sampled on a 300 μm × 5 mm PepMap C18 precolumn (Thermo Fisher Scientific) and separated in a 75 μm × 250 mm C18 column (Aurora Ultimate, 1.7 μm, IonOpticks) using a 2h-long acetonitrile gradient.

For total proteome analysis, peptides and proteins were identified and quantified by DIA-NN (version 1.9.2)^44^ using a library-free mode with the *Toxoplasma gondii* database (ME49 taxonomy, version 68 downloaded from ToxoDB), the UniProt database (*Homo sapiens* taxonomy, 202504 version), and a homemade database containing the sequences of contaminants classically identified in proteomic datasets. The digestion enzyme was set to Trypsin/P, up to 2 missed cleavages were allowed, and the peptide range was set from 6 to 30 amino acids. Precursor charge range was set from 1 to 4, while precursor m/z range was set to 400–1001 m/z and fragment ion m/z range to 120–2000 m/z. Carbamidomethylation (C) was used as a fixed modification, and N-term M excision, Oxidation (M), and Acetylation (N-Ter) were used as variable modifications. Precursor FDR was set to 1%, and the match between runs (MBR) option was activated.

For acetylome analysis, peptides and proteins were identified with Mascot (version 3.1.0, Matrix Science), as described above, except that 4 missed cleavages were allowed and Acetyl (K, variable) was also used in peptide modifications. The Proline software^40^ was used for the compilation, grouping, and filtering of the results, as described above, and for the quantification of peptidoforms.

Statistical analyses were performed using Prostar^43^ as described above, except that thresholds of log2(Fold Change) and *p*-values were set to 1 and 0.01, respectively, leading to false discovery rates inferior to 1% according to the Benjamini–Hochberg estimator.

### Identification of *Tg*GCN5b-dependent acetylation sites

To identify acetylation events dependent on *Tg*GCN5b activity, quantitative acetylome and total proteome datasets were compared between untreated parasites (UT) and parasites depleted of *Tg*GCN5b by IAA treatment. For each quantified acetyl-lysine (AcK) site, differential abundance was assessed using log2-transformed fold changes [log2(UT/IAA)] and associated limma-derived *P* values. AcK sites were considered significantly reduced following TgGCN5b depletion when they met the thresholds log2(UT/IAA) ≥ 1 and *P* < 0.01. To distinguish site-specific acetylation changes from changes resulting from altered protein abundance, AcK measurements were compared with the corresponding total proteome data. Candidate *Tg*GCN5b-dependent acetylation sites were defined as AcK sites significantly enriched in untreated parasites that could not be explained by a corresponding increase in total protein abundance. A site-dependency score was calculated as: AcK dependency score = AcK log2(UT/IAA) − protein log2(UT/IAA). Using these criteria, 135 candidate *Tg*GCN5b-dependent acetylation sites were identified across 99 proteins.

### Gene ontology enrichment analysis of the TgGCN5b-dependent acetylome

GO enrichment analysis was performed on proteins carrying candidate *Tg*GCN5b-dependent acetylation sites. The background set consisted of proteins with at least one quantified acetylation site and an associated GO annotation. Biological Process, Molecular Function, and Cellular Component ontologies were analyzed separately. Enrichment was assessed using a one-sided hypergeometric test, and *P* values were corrected for multiple testing using the Benjamini–Hochberg procedure within each ontology category. GO terms represented by at least two candidate proteins were retained for visualization. Enrichment significance was reported as −log10(Benjamini–Hochberg-adjusted *P* value), and point size reflected the number of candidate proteins associated with each term.

### Chromatin immunoprecipitation coupled with Illumina sequencing

#### Chromatin immunoprecipitation

HFF cells were grown to confluence and infected with KD strains as indicated in the figure legends. Harvested intracellular parasites were cross-linked with formaldehyde (final concentration 1%) for 8 min at room temperature, and cross-linking was stopped by the addition of glycine (final concentration 0.125 M) for 5 min at room temperature. The parasites were lysed in ice-cold lysis buffer A (50 mM HEPES KOH pH7.5, 140 mM NaCl, 1 mM EDTA, 10% glycerol, 0.5% NP-40, 0.125% Triton X-100, protease inhibitor cocktail) and after centrifugation, cross-linked chromatin was sheared in buffer B (1 mM EDTA pH 8.0, 0.5 mM EGTA pH 8.0, 10 mM Tris pH 8.0, protease inhibitor cocktail) by sonication with a Diagenode Biorupter. Samples were sonicated for 16 cycles (30 s ON and 30 s OFF) to achieve an average size of 200–500 base pairs. Sheared chromatin, 5% BSA, a protease inhibitor cocktail, 0.1% Triton X-100, 0.1% deoxycholate, magnetic beads coated with DiaMag protein A (Diagenode) and antibodies against epitope tags (HA or MYC), HDAC3, or MORC were used for immunoprecipitation. A rabbit IgG antiserum served as a control mock. After overnight incubation at 4 °C on a rotating wheel, chromatin-antibody complexes were washed and eluted from the beads using the iDeal ChIP-seq kit for transcription factors (Diagenode) according to the manufacturer’s protocol. Samples were de-crosslinked by heating for 4 h at 65 °C. DNA was purified using the IPure kit (Diagenode) and quantified using Qubit Assays (Thermo Fisher Scientific) according to the manufacturer’s protocol. For ChIP-seq, the purified DNA was used for library preparation and subsequently sequenced by Arraystar Co (USA). *Library Preparation, Sequencing, and Data analysis (Arraystar)*. ChIP sequencing libraries were prepared according to the Illumina protocol Preparing Samples for ChIP Sequencing of DNA. Library preparation: 10 ng of DNA from each sample was converted to blunt-end phosphorylated DNA fragments using T4 DNA polymerase, Klenow polymerase, and T4 polymerase (NEB); an “A” base was added to the 3’ end of the blunt-end phosphorylated DNA fragments using the polymerase activity of Klenow (Exo-Minus) polymerase (NEB); Illumina genomic adapters were ligated to the A-tailed DNA fragments; PCR amplification to enrich the ligated fragments was performed using Phusion High Fidelity PCR Master Mix with HF Buffer (Finnzymes Oy). The enriched product of ~200–700 bp was excised from the gel and purified. Sequencing: the library was denatured with 0.1 M NaOH to generate single-stranded DNA molecules and loaded into flow cell channels at a concentration of 8 pM and amplified in situ using TruSeq Rapid SR cluster kit (# GD-402-4001, Illumina). Sequencing was performed at 100 cycles on the Illumina HiSeq 4000 according to the manufacturer’s instructions.

#### Data analysis

After the sequencing platform generated the sequencing images, the stages of image analysis and base calling were performed using Off-Line Basecaller software (OLB V1.8). After passing the Solexa CHASTITY quality filter, the clean reads were aligned to the *T. gondii* reference genome (TGME49) using BOWTIE V2, then converted and sorted using Bamtools. Aligned reads were used for peak calling of the ChIP-enriched peaks using MACS V2.2 with a cutoff *p*-value of 10^−4^. Data visualization: For IGB visualization and gene-centered analysis using Deeptools, MACS2-generated bedgraph files were processed with the following command: “sort -k1,1 -k2,2n 5_treat_pileup.bdg > 5_treat_pileup-sorted.bdg” then converted using the BedGraphToBigWig program (ENCODE project). The Deeptools analysis was generated using “computeMatrix reference point” with the following parameters (--minThreshold 2, --binSize 10 and –averageTypeBins mean). Plot profile or heatmap was then used with k-means clustering when applicable. Inter-sample comparisons were obtained using the nf-core chip-seq workflow with standard parameters. All these raw and processed files can be found at GSE313273.

### RNA-seq and sequence alignment

Total RNA was extracted and purified using TRIzol (Invitrogen, Carlsbad, CA, USA) and RNeasy Plus Mini Kit (Qiagen). RNA quantity and quality were measured by NanoDrop 2000 (Thermo Scientific). For each condition, RNA was prepared from three biological replicates. RNA integrity was assessed by standard non-denaturing 1.2% TBE agarose gel electrophoresis. RNA sequencing was performed following standard Illumina protocols by Novogene (Cambridge, United Kingdom). Briefly, RNA quantity, integrity, and purity were determined using the Agilent 5400 Fragment Analyzer System (Agilent Technologies, Palo Alto, California, USA). The RQN ranged from 7.8 to 10 for all samples, which was considered sufficient. Messenger RNAs (mRNA) were purified from total RNA using poly-T oligo-attached magnetic beads. After fragmentation, the first-strand cDNA was synthesized using random hexamer primers. Then the second-strand cDNA was synthesized using dUTP instead of dTTP. The directional library was ready after end repair, A-tailing, adapter ligation, size selection, USER enzyme digestion, amplification, and purification. The library was checked with Qubit and real-time PCR for quantification and a bioanalyzer for size distribution detection. Quantified libraries will be pooled and sequenced on Illumina platforms, according to effective library concentration and data amount. The samples were sequenced on the Illumina NovaSeq platform (2 × 150 bp, strand-specific sequencing) and generated ~40 million paired-end reads for each sample. The quality of the raw sequencing reads was assessed using FastQC (www.bioinformatics. babraham.ac.uk/projects/fastqc/) and MultiQC. For expression data quantification and normalization, the FASTQ reads were aligned to the ToxoDB-49 build of the *T. gondii* ME49 genome using Subread version 2.0.1 with the following options “subread-align -d 50 -D 600 --sortReadsByCoordinates”. Read counts for each gene were calculated using featureCounts from the Subread package. Differential expression analysis was conducted using DESeq2 and default settings within the iDEP.96 web interface. Transcripts were quantified and normalized using TPMCalculator. The Illumina RNA-seq dataset generated during this study is available at GSE313582.

### Assay for transposase-accessible chromatin with high-throughput sequencing (ATAC-seq)

Intracellular tachyzoites (non-treated or IAA-treated for 24 h) were prepared using HFF cell monolayers in a T175 format, which were freshly scraped, gently homogenized by pipetting, and centrifuged at 500 × *g*. Prior to initiating the transposition protocol, the pellet was gently washed with warm DPBS (Life Technologies) and resuspended in 500 μl of cold PBS + protease inhibitor (Diagenode kit). Nuclei preparation, permeabilization, Tn5 transposition, and library preparation were prepared following precisely the Diagenode ATAC-SEQ kit protocol (C01080002). Nuclei permeabilization was performed on an estimated 100,000 tachyzoites by diluting 10 μl of DPBS cell suspension (from one T175 resuspended in 500 μl) in 240 of DPSB + protease inhibitor (1/25 dilution). From this dilution, 50 μl were then taken to perform the transposition reaction. Of note, the permeabilization protocol used a 3 min 0.02% digitonin (Promega) treatment. Following the Tn5 reaction, libraries were amplified using the Diagenode 24 UDI kit 1 (ref 01011034) following standard protocol procedures. Libraries were multiplexed and sequenced on a single Novaseq6000 lane by Fasteris (Genesupport SA) using 2 × 50 cycles, generating on average 27 million reads. Following the demultiplexing of raw reads by bcl2fastq V3, trimming and quality control, reads were aligned to the *T. gondii* reference genome (TGME49) using BOWTIE V2. For the NLR analysis, the frequencies of aligned read lengths were obtained, and a loess model was then fitted to the dispersion graph of the log frequencies versus the log of fragment length using the R function loess with a span = 0.5. The residuals of that model were then smoothed using the R function loess with a span = 0.2. The modes corresponding to mono-, di-, and tri-nucleosomal fragments were identified by finding the local maximums of the smoothed residuals. Then, three separate approximations of the nucleosome repeat length were determined for every sample. This was achieved by dividing the lengths of the mono-, di-, and tri-nucleosomal fragments by 1, 2, and 3, respectively. These results were then averaged to generate a singular estimation for each sample. Statistical significance was calculated using a one-sided Welch two-sample t-test to compare UT to IAA.

On the other hand, aligned reads were used for peak calling of the ChIP-enriched peaks using Generich with a cutoff *p*-value of 0.1. Data visualization: For IGB visualization and gene-centered analysis using Deeptools, MACS2-generated bedgraph files were processed with the following command: “sort -k1,1 -k2,2n 1_treat_pileup.bdg > 1_treat_pileup-sorted.bdg” then converted using the BedGraphToBigWig program (ENCODE project). Reads were counted on annotated peaks by featureCounts, and counts were processed by DeSeq2 to generate a global statistical analysis of peak intensities between conditions using biological triplicates. All these raw and processed files can be found in Series GSE313048. Data visualization: For IGB visualization and gene-centered analysis using Deeptools, Picard merged bam files were converted to bigWig file format using a bin size of 5 by bamCoverage (Deeptools). The Deeptools analysis was then generated using “computeMatrix reference point” with the following parameters (--minThreshold 2, --binSize 5, and –averageTypeBins mean).

### ChIP–seq signal visualization

Metagene profiles and heatmaps were generated using deepTools v3.5.1. Normalized ChIP–seq bigWig files (counts per million, CPM) were processed with computeMatrix in either reference-point mode (±2 kb around transcription start sites [TSS] and transcription end sites [TES]) or scale-regions mode, in which gene bodies were scaled to a uniform length of 5 kb. A bin size of 5 bp was used throughout. Average signal profiles and heatmaps were generated using plotProfile and plotHeatmap, respectively. Analyses were performed using annotated gene models from ToxoDB release 67.

### Correlation between chromatin occupancy and gene expression

To assess the relationship between chromatin occupancy and transcriptional output, the normalized ChIP–seq signal was quantified within a ±500 bp window surrounding each annotated TSS and compared with corresponding RNA-seq expression values. Expression values were represented as log-transformed CPM. Correlations were calculated using Spearman’s rank correlation coefficient implemented in the Python package SciPy (scipy.stats.spearmanr), and significance was assessed using a two-tailed test.

### Chromatin occupancy stratified by gene expression

To examine the relationship between chromatin occupancy and transcriptional activity, genes were ranked according to RNA-seq expression levels (CPM) and divided into five equally sized groups (quintiles). For each quintile, the distribution of ChIP–seq signal at promoter regions was visualized as box plots showing the median, interquartile range, and outliers.

### De novo motif discovery

De novo motif discovery was performed using HOMER (findMotifsGenome.pl). ChIP–seq peak regions were analyzed using ±200 bp windows centered on peak summits. The highest-ranked motifs identified for TgGCN5b and TgPHD1 were further analyzed for genomic occurrence using scanMotifGenomeWide.pl, and genes associated with motif-containing peaks were extracted for downstream analyses.

### Gene ontology enrichment analysis

GO enrichment analysis was performed on genes associated with motif-containing ChIP–seq peaks using the g web server and API. Analyses were restricted to the Molecular Function (GO) ontology. Statistical significance was assessed using the g multiple-testing correction method, with an adjusted significance threshold of 0.05. Enriched terms were ranked according to significance and visualized as dot plots, with dot size proportional to enrichment significance (−log10 adjusted *P* value).

### Protein structure predictions

AlphaFold2 predictions^45^ were run through the ColabFold^46^ workflow (v 1.3.0) on an Nvidia A5000 graphics card, while AlphaFold3 predictions were run on the Google AlphaFold server or installed on a workstation with parameter access granted to CS by the Google AlphaFold Team. Key input parameters were as follows: --num-recycle 3 --random-seed 30. All models were depicted using UCSF ChimeraX^47^.

### Statistics and reproducibility

Sample sizes were not predetermined and were chosen according to previous literature. Experiments were performed in biological replicates and provided consistent, statistically relevant results. No method of randomization was used. All experiments were performed in independent biological replicates as stated for each experiment in the manuscript. Specifically, the size-exclusion chromatography experiment (Fig. 1c), immunoprecipitation experiment (Fig. 2f), and western blot analysis (Fig. 4b) were each performed independently twice, with similar results. Immunofluorescence microscopy assays (Fig. 4a) were repeated independently three times with identical results. All corresponding treatment and control samples from ChIP-seq, RNA-seq, and ATAC-seq were processed at the same time to minimize technical variation. Investigators were not blinded during the experiments. Statistical significance was evaluated using *P* values from unpaired two-tailed Student *t*-tests. Data are presented as the mean ± s.d. Significance was set to a *P* value of <0.05. All the micrographs shown are representatives from three independently conducted experiments, with similar results obtained.

### Reporting summary

Further information on research design is available in the Nature Portfolio Reporting Summary linked to this article.

## Supplementary information

Supplementary Information File

Supplementary Dataset 1

Supplementary Dataset 2

Supplementary Dataset 3

Reporting Summary

Transparent Peer Review File

## Data Availability

Illumina RNA Sequencing data that support the findings of this study have been deposited under the GSE313582. The ChIPseq and ATAC-seq data have been deposited to the GEO Datasets under accession numbers GSE313273 and GSE313048, respectively. The mass spectrometry proteomics data have been deposited to the ProteomeXchange Consortium via the PRIDE partner repository with the dataset identifiers PXD072342 for *Tg*GCN5b, *Tg*PHD1, *Tg*PZP1 and *Tg*GNAT1 interactomes, PXD079431 for *Tg*GCN5b interactome in PHD1 KD or AP2XI-2 and AP2XII-1 KD parasites, *Tg*GCN5b interactome from simple and double purifications, and *Tg*GNAT2 interactome and PXD079432 for *Tg*GCN5b-dependent acetylome and proteome modulations (https://proteomecentral.proteomexchange.org/cgi/GetDataset?ID=PXD079432). Source data are provided with this paper.
