# Supplementary material for: Divergent SAGA complexes shape the Toxoplasma transcriptome for lytic cycle progression and host interaction
Source: Nat Commun. 2026 Aug 25;17:10158. doi: 10.1038/s41467-026-77153-4 (PMC13601556; doi:10.1038/s41467-026-77153-4)
Supplement: Supplementary file 1 [file 41467_2026_77153_MOESM1_ESM.pdf]

Supplementary Information

Divergent SAGA Complexes Shape the *Toxoplasma* Transcriptome for Lytic Cycle Progression and Host Interaction

Dominique Cannella, Belen Pachano, Martina Shahinas, Lucid Belmudes, Jon deVries, Charlotte Corrao, Léa Pounot, Anne-Marie Hesse, Yohann Couté, Alexandre Bougdour, Christopher Swale and Mohamed-Ali Hakimi

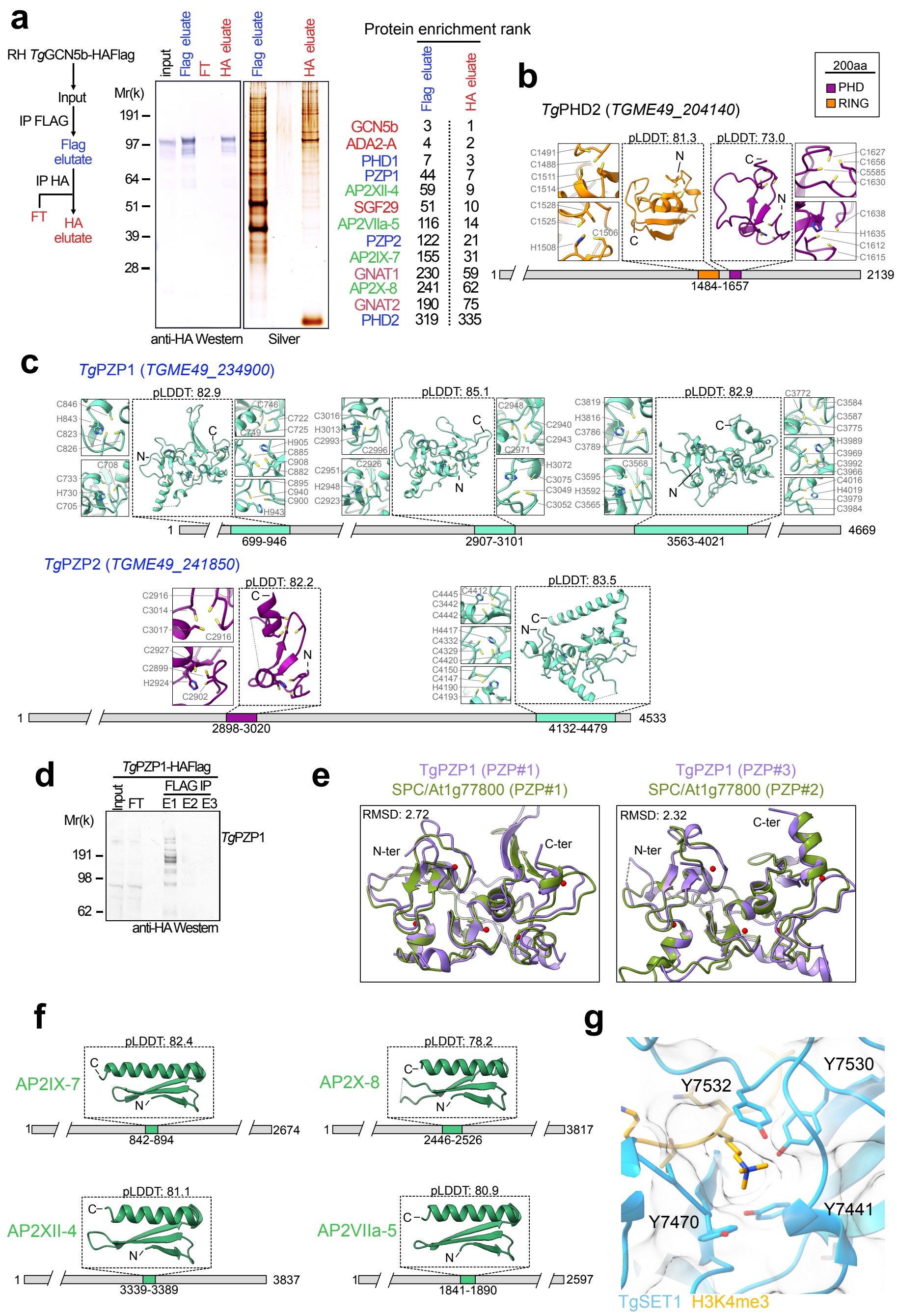

Supplementary Fig. 1 | AlphaFold-Based Structural Predictions of TgGCN5b-associated proteins. a, Sequential purification of *Tg*GCN5b-associated proteins. *Tg*GCN5b-HAFlag-expressing RH parasites were subjected to FLAG affinity purification followed by HA affinity purification. Input, flow-through (FT), FLAG eluate, and HA eluate fractions were examined by anti-HA Western blotting and silver staining. The table summarizes the enrichment ranks of proteins detected in the FLAG and HA eluates, identifying candidate components of the *Tg*GCN5b complex. **b**, AlphaFold-predicted domain organizations of TgPHD2. High per-residue confidence (pLDDT scores indicated for each model) supports the structural integrity of PHD, and RING domains. Conserved cysteine- and histidine-rich motifs characteristic of chromatin-binding modules are highlighted. **c**, AlphaFold-predicted domain architectures of *Tg*PZP1 and *Tg*PZP2. High per-residue confidence values (pLDDT scores indicated) support the structural integrity of their PZP and PHD domains. Conserved cysteine- and histidine-rich motifs characteristic of chromatin-associated modules are highlighted. **d**, *Tg*PZP1-associated proteins were affinity-purified by Flag chromatography from HFF cells infected with RH parasites expressing *Tg*PZP1-HA-Flag. Input, flow-through, and elution fractions (E1–E3) were analyzed by immunoblotting using anti-HA antibodies to detect *Tg*PZP1. **e**, AlphaFold models of PZP#1 from TgPZP1 (pLDDT: 82.9) were structurally aligned in ChimeraX to PZP#1 of AT1G77800 (pLDDT: 90.27), and PZP#3 from TgPZP1 (pLDDT: 82.9) was aligned to PZP#2 of AT1G77800 (pLDDT: 80.25). Representative zinc atoms coordinating the PHD domains are highlighted in red. **f**, AlphaFold-predicted domain organizations of *Tg*GCN5b-associated AP2 transcription factors with pLDDT scores supporting the structural integrity of AP2 domain. **g**, AlphaFold prediction of a Tyr-rich aromatic pocket in *Tg*SET1 capable of accommodating H3K4me3. Models shown: *Tg*SET1 pocket (pLDDT: 74.95) and *Tg*H3K4me3 peptide (pLDDT: 71.15).

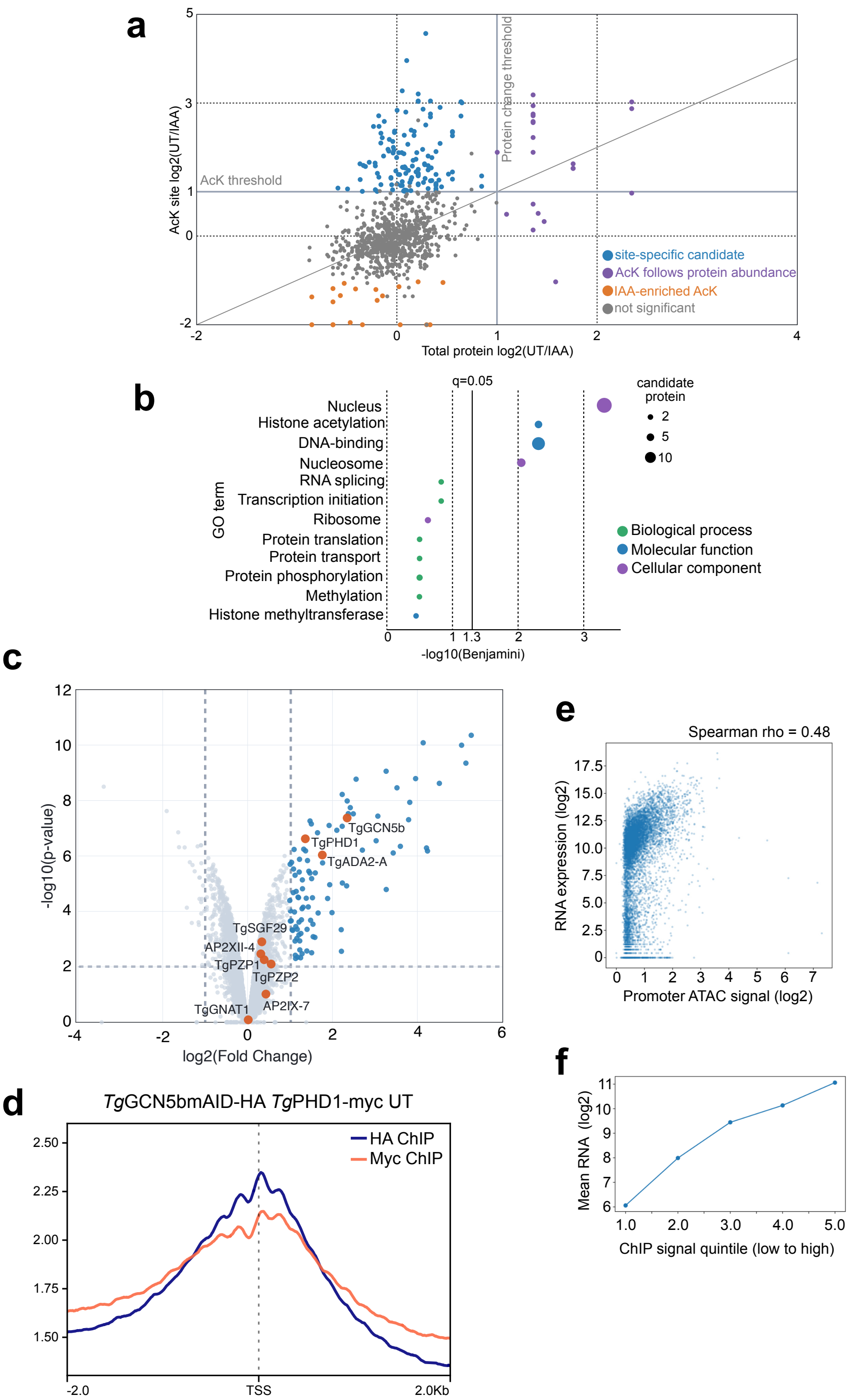

Supplementary Fig. 2 | Acetylome, proteome and chromatin features associated with *Tg*GCN5b function. a, Comparison of acetyl-lysine (AcK) site abundance and corresponding protein abundance changes following TgGCN5b depletion. Each point represents a quantified AcK site. Blue points indicate candidate TgGCN5b-dependent acetylation sites enriched in untreated parasites relative to IAA-treated parasites and whose variation exceeds the corresponding protein abundance change. Purple points indicate AcK sites whose variation follows protein abundance, orange points indicate IAA-enriched AcK sites, and grey points indicate non-significant sites. **b,** Gene Ontology (GO) enrichment analysis of proteins carrying candidate TgGCN5b-dependent acetylation sites identified in panel a. Biological process, molecular function and cellular component categories are shown. The x-axis represents −log10(Benjamini–Hochberg-adjusted P value), and dot size indicates the number of proteins associated with each GO term. **c,** Volcano plot showing changes in total protein abundance following *Tg*GCN5b depletion. Highlighted proteins in orange correspond to components of the *Tg*GCN5b complex. **d,** Average ChIP-seq signal profiles for *Tg*GCN5b (HA ChIP) and *Tg*PHD1 (Myc ChIP) across TSS (±2 kb, 100 bp bins) under untreated conditions. The nearly superimposed HA and Myc enrichment patterns indicate strong co-localization of both proteins at promoter regions. **e,** Relationship between promoter accessibility and gene expression across annotated T. gondii genes. Scatter plot showing promoter ATAC-seq signal (log2-transformed) versus transcript abundance (log2-transformed RNA-seq expression values). Spearman's correlation coefficient ($\rho = 0.48$) is indicated. **f,** Mean transcript abundance as a function of *Tg*GCN5b promoter occupancy. Genes were grouped into quintiles based on TgGCN5b ChIP-seq signal at their promoters, and the mean RNA expression level (log2-transformed) was calculated for each quintile.

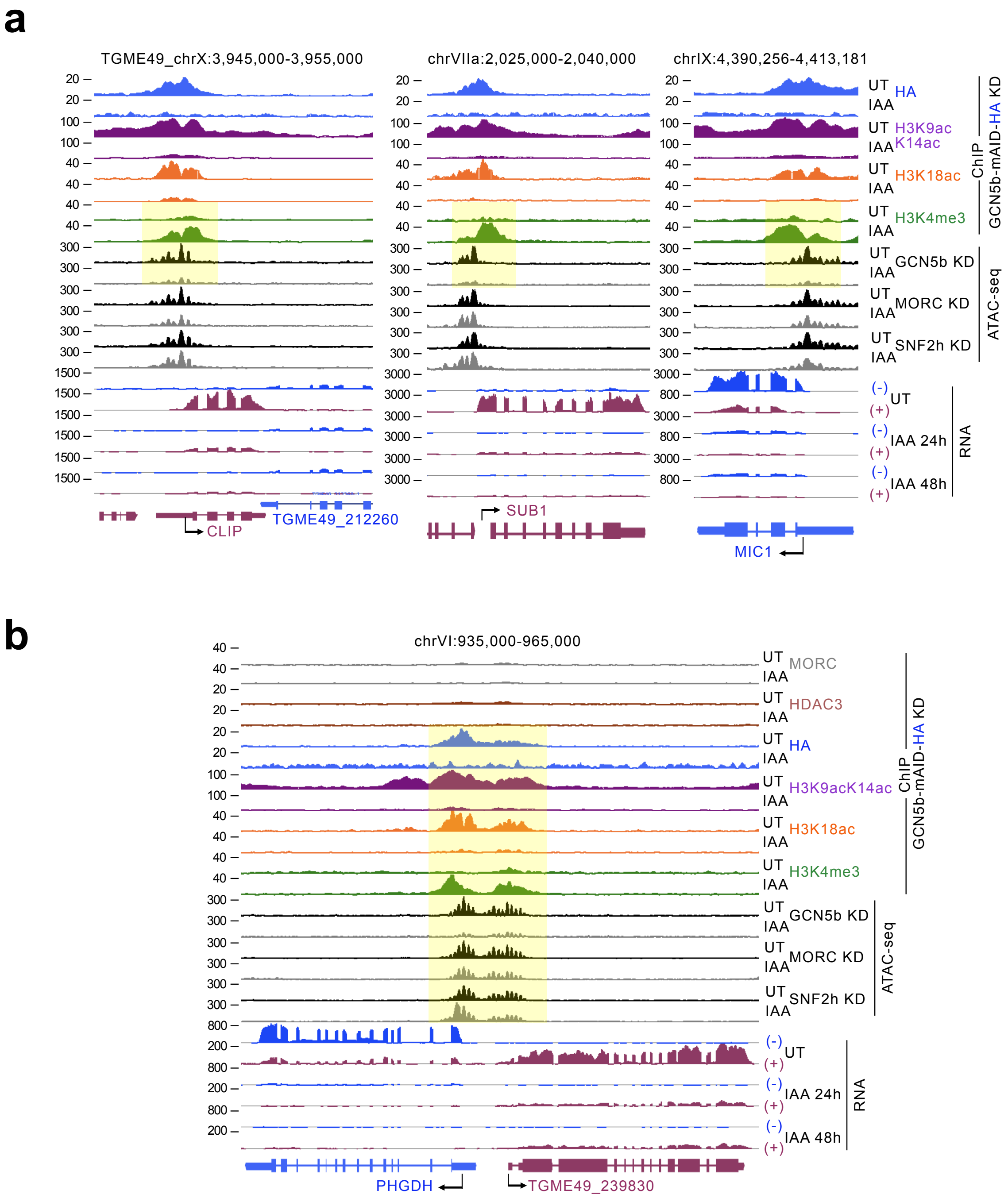

Supplementary Fig. 3 | Representative metabolic and invasion-associated loci displaying *Tg*GCN5b-dependent chromatin remodeling. a, Genome browser views of representative invasion-associated loci encoding *CLIP, SUB1* and *MIC1*. Tracks show *Tg*GCN5b-HA, H3K9acK14ac, H3K18ac and H3K4me3 ChIP–seq profiles together with matching ATAC-seq and RNA-seq datasets in untreated and *Tg*GCN5b-depleted parasites. ATAC-seq profiles from *Tg*GCN5b, MORC and *Tg*SNF2h knockdown parasites are included for comparison. These loci exemplify the chromatin transition highlighted in yellow and observed across Cluster 2 (~300 direct *Tg*GCN5b targets), characterized by loss of histone acetylation and chromatin accessibility together with increased H3K4me3 following *Tg*GCN5b depletion. (+), forward strand; (−), reverse strand. **b**, Genome browser view of the PHGDH locus and neighboring genes. Tracks show ChIP–seq profiles for MORC, HDAC3, TgGCN5b-HA, H3K9acK14ac, H3K18ac and H3K4me3 in untreated (UT) and IAA-treated TgGCN5b-mAID-HA parasites, together with corresponding ATAC-seq and RNA-seq datasets collected 24 h and 48 h after auxin treatment. ATAC-seq profiles from TgGCN5b, MORC and TgSNF2h knockdown parasites are shown for comparison. The shaded region highlights a highly open chromatin site whose accessibility is sensitive to *Tg*GCN5b loss.

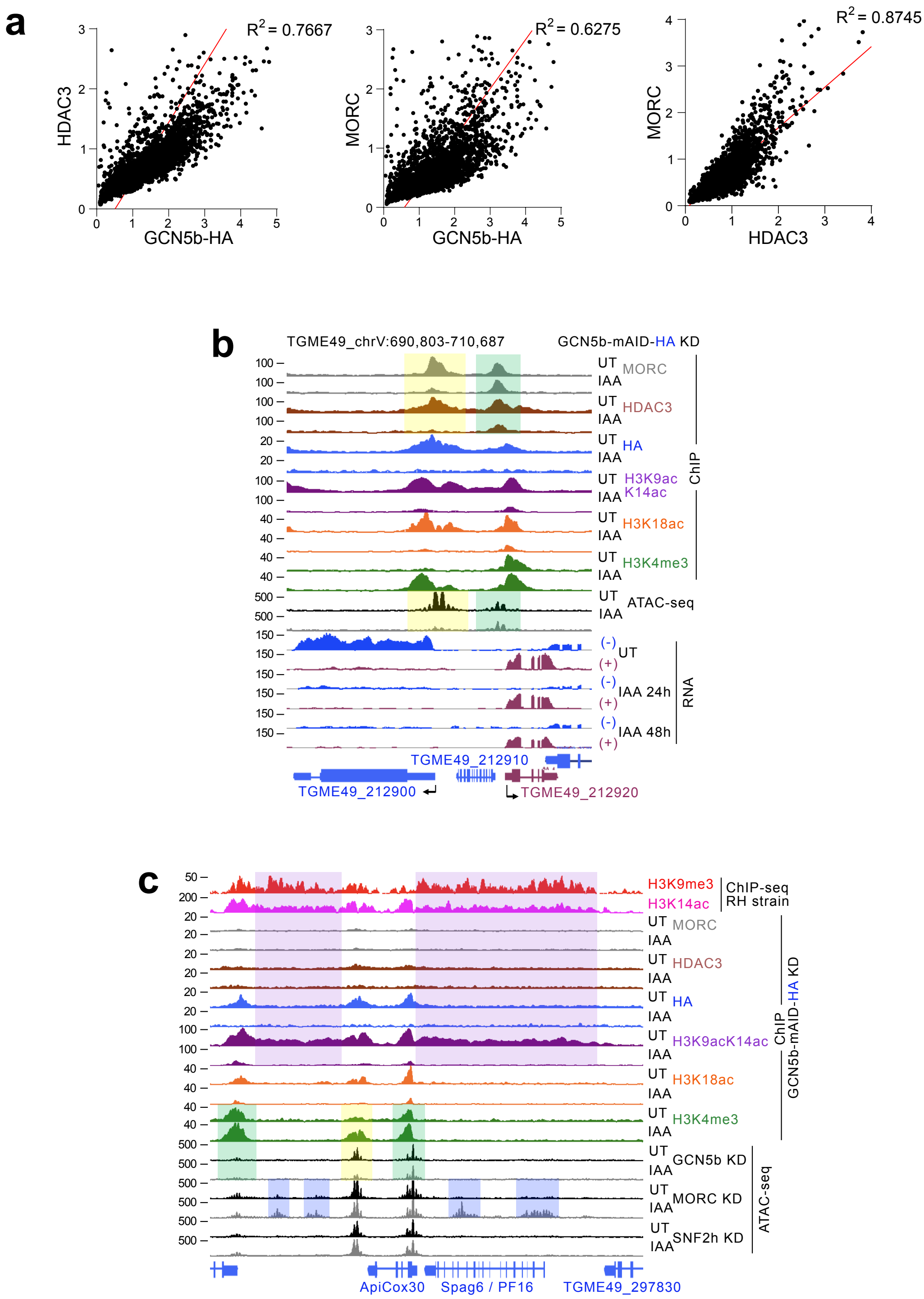

Supplementary Fig. 4 | Relationship between *Tg*GCN5b, HDAC3 and MORC occupancy and representative bimodal chromatin domains. a, Pairwise scatterplots comparing ChIP–seq signal intensities for TgGCN5b-HA, HDAC3 and MORC across genomic regions. Each dot represents a ChIP–seq peak. Linear regression fits and corresponding R^2 values are indicated. **b**, Genome browser view of a representative locus on chromosome V. Tracks show ChIP–seq profiles for MORC, HDAC3, *Tg*GCN5b-HA, H3K9acK14ac, H3K18ac and H3K4me3 in untreated (UT) and IAA-treated *Tg*GCN5b-mAID-HA parasites, together with corresponding ATAC-seq and RNA-seq datasets collected 24 h and 48 h after auxin treatment. The yellow box highlights a chromatin region with strong accessibility that diminishes upon *Tg*GCN5b depletion, whereas the green box marks a poised chromatin site with more limited sensitivity to *Tg*GCN5b loss. These same-colored regions also illustrate the persistence of MORC and HDAC3 binding, consistent with their preferential association with open or poised chromatin states. **c**, Genome browser view of a representative bimodal chromatin domain encompassing *ApiCox30*, the sexual-stage gene *PF16* (*Spag6*), and *TGME49_297830*. ChIP–seq profiles for H3K9me3, H3K14ac, MORC, HDAC3, *Tg*GCN5b-HA, H3K9acK14ac, H3K18ac and H3K4me3 are shown together with matching ATAC-seq and RNA-seq datasets. ATAC-seq profiles from *Tg*GCN5b, MORC and TgSNF2h knockdown parasites are included for comparison. Colored boxes denote adjacent chromatin domains exhibiting distinct accessibility and histone modification signatures. (+), forward strand; (−), reverse strand.
