# Supplementary material for: Divergent SAGA complexes shape the Toxoplasma transcriptome for lytic cycle progression and host interaction
Source: Nat Commun. 2026 Aug 25;17:10158. doi: 10.1038/s41467-026-77153-4 (PMC13601556; doi:10.1038/s41467-026-77153-4)
Supplement: Supplementary file 2 [file 41467_2026_77153_MOESM5_ESM.pdf]

nature portfolio

Corresponding author(s): HAKIMI Mohamed-Ali

Last updated by author(s): 2026/07/25

Reporting Summary

Nature Portfolio wishes to improve the reproducibility of the work that we publish. This form provides structure for consistency and transparency in reporting. For further information on Nature Portfolio policies, see our Editorial Policies and the Editorial Policy Checklist.

Statistics

For all statistical analyses, confirm that the following items are present in the figure legend, table legend, main text, or Methods section.

n/a	Confirmed	
☐	☒	The exact sample size (*n*) for each experimental group/condition, given as a discrete number and unit of measurement
☒	☐	A statement on whether measurements were taken from distinct samples or whether the same sample was measured repeatedly
☐	☒	The statistical test(s) used AND whether they are one- or two-sided<br>*Only common tests should be described solely by name; describe more complex techniques in the Methods section.*
☒	☐	A description of all covariates tested
☒	☐	A description of any assumptions or corrections, such as tests of normality and adjustment for multiple comparisons
☐	☒	A full description of the statistical parameters including central tendency (e.g. means) or other basic estimates (e.g. regression coefficient) AND variation (e.g. standard deviation) or associated estimates of uncertainty (e.g. confidence intervals)
☐	☒	For null hypothesis testing, the test statistic (e.g. *F*, *t*, *r*) with confidence intervals, effect sizes, degrees of freedom and *P* value noted<br>*Give P values as exact values whenever suitable.*
☒	☐	For Bayesian analysis, information on the choice of priors and Markov chain Monte Carlo settings
☒	☐	For hierarchical and complex designs, identification of the appropriate level for tests and full reporting of outcomes
☐	☒	Estimates of effect sizes (e.g. Cohen's *d*, Pearson's *r*), indicating how they were calculated

Our web collection on statistics for biologists contains articles on many of the points above.

Software and code

Policy information about availability of computer code

Data collection	Librairies were sequenced on an Illumina NovaSeq platform and collected in FastQ format. MS and MS/MS data were acquired using Xcalibur software version 4.0 (Thermo Scientific). Peptides and proteins were identified using Mascot (version 2.8).
Data analysis	Integrated Genome Browser - by Nowlan H. Freese, David C. Norris, and Ann E. Loraine, Bioinformatics 2016 Jul 15;32(14):2089-95.<br>GraphPad - Prism 7<br>R (version 3.5.3), R Development Core Team, 2012<br>blastp (version 2.2.31+), Altschul et al., 1990<br>BOWTIE (V2.1.0)<br>Samtools v1.4<br>Bamtools v2.5.1<br>Guppy v5 or higher<br>minimap2 v2.17<br>Deeptools v3.5.1 was used for global peak intensity over gene features.<br>Basecaller software (OLB V1.8)<br>SeqMan NGen (version 14, DNASTAR. Madison, WI, USA)<br>Lasergene Genomics Suite version 14 (DNASTAR, Madison, WI, USA)<br>ArrayStar module (version 14, DNASTAR. Madison, WI, USA)<br>ClustalW software<br>RAW files were processed using MaxQuant version 1.6.2.10.<br>Proline software (http://proline.profiproteomics.fr) was used to filter the results.<br>Statistical analyses were performed using ProStaR (Wieczorek, S. et al. DAPAR & ProStaR: software to perform statistical analyses in

quantitative discovery proteomics. Bioinformatics 33, 135–136 (2017).
nf-core ATAC-SEQ v2.0 is a nextflow pipeline for ATAQ-SEQ data processing
Integrated Genome Browser (IGB) v9.1.8 or higher

For manuscripts utilizing custom algorithms or software that are central to the research but not yet described in published literature, software must be made available to editors and reviewers. We strongly encourage code deposition in a community repository (e.g. GitHub). See the Nature Portfolio guidelines for submitting code & software for further information.

Data

Policy information about availability of data

All manuscripts must include a data availability statement. This statement should provide the following information, where applicable:

- Accession codes, unique identifiers, or web links for publicly available datasets
- A description of any restrictions on data availability
- For clinical datasets or third party data, please ensure that the statement adheres to our policy

Illumina RNA Sequencing data that support the findings of this study have been deposited under the GSE313582. The ChIPseq and ATAC-seq data have been deposited to the GEO Datasets under accession number GSE313273 and GSE313048, respectively. The mass spectrometry proteomics data have been deposited to the ProteomeXchange Consortium via the PRIDE partner repository with the dataset identifier PXD072342.

ATAC-seq data
Series GSE313048
https://www.ncbi.nlm.nih.gov/geo/query/acc.cgi?acc=GSE313048

ChIPseq data
Series GSE313273
https://www.ncbi.nlm.nih.gov/geo/query/acc.cgi?acc=GSE313273

RNAseq
Series GSE313582
https://www.ncbi.nlm.nih.gov/geo/query/acc.cgi?acc=GSE313582

PRIDE Mass spect :
Project accession: PXD072342 (TgGCN5b, TgPHD1, TgPZP1 and TgGNAT1 interactomes)
https://proteomecentral.proteomexchange.org/cgi/GetDataset?ID=PXD072342

Project accession: PXD079431 (TgGCN5b interactome in PHD1 KD or AP2XI-2 and AP2XII-1 KD parasites)
https://proteomecentral.proteomexchange.org/cgi/GetDataset?ID=PXD079431

Project accession: PXD079432 (TgGCN5b-dependent acetylome and proteome modulations)
https://proteomecentral.proteomexchange.org/cgi/GetDataset?ID=PXD079432

Research involving human participants, their data, or biological material

Policy information about studies with human participants or human data. See also policy information about sex, gender (identity/presentation), and sexual orientation and race, ethnicity and racism.

Reporting on sex and gender	N/A
Reporting on race, ethnicity, or other socially relevant groupings	N/A
Population characteristics	N/A
Recruitment	N/A
Ethics oversight	N/A

Note that full information on the approval of the study protocol must also be provided in the manuscript.

Field-specific reporting

Please select the one below that is the best fit for your research. If you are not sure, read the appropriate sections before making your selection.

☒ Life sciences ☐ Behavioural & social sciences ☐ Ecological, evolutionary & environmental sciences

Life sciences study design

All studies must disclose on these points even when the disclosure is negative.

Sample size	Sample sizes were determined because differences among groups were consistent and significant.<br>All experiments were performed in biological replicates to allow for statistical analyses.
Data exclusions	No data were excluded from the analysis
Replication	All findings were reproduced successfully, and replications are described in methods and figure legends in more detail.
Randomization	No method of randomization was used and all experiments were performed in independent biological replicates as stated for each experiment in the main manuscript. All corresponding treatment and mock samples were processed at the same time to minimize technical variation. Random images of cells were evaluated for IFA and TEM. Randomization for other experiments were not subjective.
Blinding	Investigators were not blinded during the experiments. Experiments were performed in biological replicates and provided consistent statistically relevant results. Other experiments were not performed with blinding since no subjective evaluations were involved.

Reporting for specific materials, systems and methods

We require information from authors about some types of materials, experimental systems and methods used in many studies. Here, indicate whether each material, system or method listed is relevant to your study. If you are not sure if a list item applies to your research, read the appropriate section before selecting a response.

Materials & experimental systems

n/a	Involved in the study	
☐	☒	Antibodies
☒	☐	Eukaryotic cell lines
☒	☐	Palaeontology and archaeology
☒	☐	Animals and other organisms
☒	☐	Clinical data
☒	☐	Dual use research of concern
☒	☐	Plants

Methods

n/a	Involved in the study	
☐	☒	ChIP-seq
☒	☐	Flow cytometry
☒	☐	MRI-based neuroimaging

Antibodies

Antibodies used	The following primary antibodies were used in the immunofluorescence, immunoblotting, and/or ChIP assays: rabbit anti-TgHDAC3 (Research Resource Identifier (RRID): AB_2713903), rabbit anti-TgGAP45 (gift from Pr. Dominique Soldati), mouse anti-HA tag (Roche, RRID: AB_2314622), rabbit anti-HA Tag (Cell Signaling Technology, RRID: AB_1549585), rabbit anti-FLAG (Cell Signaling Technology, RRID: AB_2798687) and mouse anti-MYC clone 9B11 (RRID: AB_2148465). Rabbit polyclonal antibodies raised against modified histones: H3K9me3 (Diagenode, RRID: AB_2904605), anti-acetyl-histone H4, pan (Lys5,8,12) (Millipore, RRID: AB_310270), H3K9/14ac (Diagenode, RRID: AB_2637059), H3K4me3 (Diagenode, RRID: AB_2616052), H3K14ac (Diagenode, RRID: AB_2891338), H3K18ac (Diagenode, RRID: AB_2713907), H3K9ac (Diagenode, RRID: AB_2713905), H4K8ac (Diagenode, C15310103). We have also raised homemade antibodies against linear peptides in rabbits corresponding to MORC_Peptide2 (C+SGAPIWTGERGSGA) They were manufactured by Eurogentec and used for immunofluorescence, immunoblotting and/or chromatin immunoprecipitation. Secondary immunofluorescent antibodies were coupled with Alexa Fluor 488 or Alexa Fluor 594 (Thermo Fisher Scientific). Secondary antibodies used in Western blotting were conjugated to alkaline phosphatase (Promega).
Validation	All the antibodies used in this study were validated for ChIP-seq, IFA or Western blot by several groups according to their RRID numbers and sometimes by the company that own them. TgGAP45 ab is a well known antibody in Toxoplasma gondii and was validated by several publications in our field.

Plants

Seed stocks

N/A

Novel plant genotypes

N/A

Authentication

N/A

ChIP-seq

Data deposition

☒ Confirm that both raw and final processed data have been deposited in a public database such as GEO.

☒ Confirm that you have deposited or provided access to graph files (e.g. BED files) for the called peaks.

Data access links
May remain private before publication.

ChIPseq data
Series GSE313273
Token:

To review GEO accession GSE313273:
Go to https://www.ncbi.nlm.nih.gov/geo/query/acc.cgi?acc=GSE313273
Enter token gtedmgysbzeppcz into the box

Files in database submission

GSM9365096 GCN5b-KD-HA_HA-antibody_UT
GSM9365097 GCN5b-KD-HA_HA-antibody_IAA24h
GSM9365098 GCN5b-KD-HA_HDAC3-antibody_UT
GSM9365099 GCN5b-KD-HA_HDAC3-antibody_IAA24h
GSM9365100 GCN5b-KD-HA_MORC-antibody_UT
GSM9365101 GCN5b-KD-HA_MORC-antibody_IAA24h
GSM9365102 GCN5b-KD-HA_H3K18ac-antibody_UT
GSM9365103 GCN5b-KD-HA_H3K18ac-antibody_IAA24h
GSM9365104 GCN5b-KD-HA_H3K4me3-antibody_UT
GSM9365105 GCN5b-KD-HA_H3K4me3-antibody_IAA24h
GSM9365106 GCN5b-KD-HA_H3K9K14ac-antibody_UT
GSM9365107 GCN5b-KD-HA_H3K9K14ac-antibody_IAA24h
GSM9365108 GCN5b-KD-HA-PHD1-Myc_HA-antibody_UT
GSM9365109 GCN5b-KD-HA-PHD1-Myc_HA-antibody_IAA
GSM9365110 KU80_GCN5b-KD-HA-PHD1-Myc_Myc-antibody_UT
GSM9365111 GCN5b-KD-HA-PHD1-Myc_Myc-antibody_IAA
GSM9365112 PHD1-KD-HA-GCN5b-Myc_HA-antibody_UT
GSM9365113 PHD1-KD-HA-GCN5b-Myc_HA-antibody_IAA
GSM9365114 PHD1-KD-HA-GCN5b-Myc_Myc-antibody_UT
GSM9365115 PHD1-KD-HA-GCN5b-Myc_Myc-antibody_IAA

Genome browser session
(e.g. UCSC)

Not applicable

Methodology

Replicates

2 replicates / antibody

Sequencing depth

Sequencing layout: 1x150bp
Sequencing Depth for each sample ID
ID: total number of reads (Pass solexa CHASTITY quality filter)/uniquely mapped (ToxoDB-13.0_TgondiiME49)

Antibodies

rabbit anti-HA Tag (Cell Signaling Technology, RRID: AB_1549585)
rabbit MORC_Peptide2 (C+SGAPIWTGERGSGA)
mouse anti-MYC clone 9B11 (RRID: AB_2148465)
Rabbit polyclonal antibodies raised against modified histones:
H3K9/14ac (Diagenode, RRID: AB_2637059)
H3K4me3 (Diagenode, RRID: AB_2616052)
H3K14ac (Diagenode, RRID: AB_2891338)
H3K18ac (Diagenode, RRID: AB_2713907)

Peak calling parameters	After the sequencing platform generated the sequencing images, the stages of image analysis and base calling were performed using Off-Line Basecaller software (OLB V1.8). After passing Solexa CHASTITY quality filter, the clean reads were aligned to T. gondii reference genome (TGME49) using BOWTIE V2 then converted and sorted using Bamtools V2.5. Aligned reads were used for peak calling of the ChIP enriched peaks using MACS V2.2 with a cutoff p-value of 10-4. Data visualization: For IGB visualization and gene centered analysis using Deeptools, MACS2 generated bedgraph files were processed with the following command: "sort -k1,1 -k2,2n 5_treat_pileup.bdg > 5_treat_pileup-sorted.bdg" then converted using the BedGraphToBigWig program (ENCODE project). The Deeptools analysis were generated using "computeMatrix reference point" with the following parameters (--minThreshold 2, --binSize 10 and –averageTypeBins sum). Plotprofile or heatmap was then used with a k-mean clustering when applicable. Inter sample comparison were obtained using the nf-core chip-seq V2.0.0 workflow with standard parameters. From this pipeline, HOMER (annotatePeaks) was used to analyze peak distribution relative to gene features. All these raw and processed files can be found at Series GSE271900.
Data quality	Raw_data: Contains all reads which pass Solexa CHASTITY quality filter in FASTQ format (*_sequence.fastq files).
Software	BOWTIE2 software (V2.1.0)<br>MACS v2.2 (Model-based Analysis of ChIP-seq) software was used to detect the peak from ChIP-seq data.<br>Deeptools v3.5.1 was used for global peak intensity over gene features.<br>nf-core Chip-Seq v2.0.0 is a nextflow pipeline for Chip-Seq analysis.<br>Samtools v1.4<br>Bamtools v2.5.1<br>ucsc-bedgraphtobigwig v377<br>Integrated Genome Browser (IGB) v9.1.8 or higher
