# Supplementary material for: Divergent SAGA complexes shape the Toxoplasma transcriptome for lytic cycle progression and host interaction
Source: Nat Commun. 2026 Aug 25;17:10158. doi: 10.1038/s41467-026-77153-4 (PMC13601556; doi:10.1038/s41467-026-77153-4)
Supplement: Supplementary file 3 [file 41467_2026_77153_MOESM6_ESM.pdf]

nature portfolio

Peer Review File

Divergent SAGA Complexes Shape the Toxoplasma Transcriptome for Lytic Cycle Progression and Host Interaction

Corresponding Author: Dr Mohamed-Ali Hakimi

This file contains all reviewer reports in order by version, followed by all author rebuttals in order by version.

Version 0:

Reviewer comments:

Reviewer #1

(Remarks to the Author)
This manuscript provides an in-depth investigation of the SAGA co-activator complex centered on TgGCN5b. Using endogenous tagging, proteomics, structural modeling, and conditional depletion, the authors show that TgGCN5b forms a modular, apicomplexan-specific complex that lacks several canonical SAGA subunits but incorporates multiple GNAT acetyltransferases, PHD and PZP chromatin-reader scaffolds, and a minimal deubiquitinase module. These adaptations generate a distinctive chromatin regulatory architecture linking lineage-specific transcription factors to histone acetylation machinery.
Functionally, TgGCN5b depletion abolishes parasite viability, arrests the lytic cycle, and causes a selective collapse of H3K9, H3K14, and particularly H3K18 acetylation, defining a characteristic tri-site acetylation signature. Genome-wide profiling shows that TgGCN5b binds promoters of highly expressed housekeeping genes as well as key virulence and invasion factors. Loss of TgGCN5b triggers widespread transcriptional repression of ribosomal, metabolic, and secretory organelle genes, accompanied by reduced chromatin accessibility, while H3K4me3 persists or even increases. This reveals an uncoupling between H3K4 methylation and productive transcription.
The study further demonstrates that in the tachyzoite, TgGCN5b operates largely independently of the MORC/HDAC3 repressive axis and of TgSNF2h remodeling activity, defining a parallel pathway governing euchromatin regulation. At developmentally regulated bimodal chromatin domains, TgGCN5b contributes H3K14 acetylation but does not override H3K9me3-mediated repression, positioning it as a modulator rather than a primary determinant of stage-specific silencing. Finally, TgGCN5b directly regulates the BFD1–BFD2 developmental circuit, linking histone acetylation to competence for chronic-stage differentiation.
Overall, this manuscript represents a significant contribution to our understanding of chromatin-based gene regulation in T. gondii.
Minor comments and questions
Figure 2C: Based on the data presented, could two or three distinct TgGCN5b-containing complexes be proposed? A schematic model illustrating the possible complex assemblies would help clarify this point.
Figure 3 legend: Please include a brief explanation of the Gap45 marker for readers not specialized in T. gondii cell biology.
Line 247 Supplementary Fig. 2b: The text states that H4K8ac and H3K4me3 are unaffected. However, H4K8ac appears to be shown in Figure 3C.
An interesting observation is that TgGCN5b depletion eliminates H3K9/14/18 acetylation while H3K4me3 remains at promoters of formerly active genes, now in a poised state. Recent work suggests that H3K4me3 primarily regulates transcriptional pausing rather than initiation (Nature 2023, doi:10.1038/s41586-023-05780-8). It would be interesting in future studies to examine whether TgGCN5b depletion affects RNAPII recruitment or promoter-proximal pausing.
Lines 282–284: A slight decrease in AP2XII-1 mRNA expression is observed upon TgGCN5b depletion. Could this contribute to the partial activation of the associated gene set?
Line 379: “Second, neither MORC nor TgSNF2h depletion phenocopies the collapse in chromatin accessibility characteristic of TgGCN5b loss…”
Conceptually, TgGCN5b maintains open chromatin, whereas MORC promotes chromatin compaction. Therefore, TgGCN5b depletion is expected to reduce accessibility, while MORC depletion should have the opposite effect. The sentence is biologically correct, but the phrasing may be confusing and could be clarified.
The authors note that in tachyzoites, TgGCN5b and the MORC/HDAC3 axis occupy distinct chromatin territories. Since

H3K9me3 in T. gondii has so far been primarily associated with MORC/HDAC3, I wonder if the H3K9me3 mark appears upon depletion of GCN5b.
Relatedly, the manuscript suggests similar organization in other life stages (Lines 514–518). If promoters of bradyzoite and merozoite genes are associated with MORC/HDAC3, could there be stage-specific regulatory interplay between SAGA and MORC/HDAC3? This question appears partially addressed by the statement in Lines 516–518:
"Supporting this idea, we have shown that TgGCN5b purified from merozoites induced in vitro by co-depletion of AP2XII-1 and AP2XI-2 retains the same partnership observed in tachyzoites (Supplementary Table)."
However, I could not locate this result or the referenced supplementary table in the manuscript. Please clarify.

Reviewer #2

(Remarks to the Author)
In this report, the authors tried to define the Gcn5b-associated proteins by IPs and the function of GCN5b by gene KD and chromatin landscape analysis. Several interesting proteins were identified associated with Gcn5b, including SGF29, PHD domain-containing proteins (e.g., PHD1), ADA2, and AP2 domain-containing proteins. KD of Gcn5b and PHD1 led to growth defects and a sharp decrease of H3K9ac, H3K14ac, and H3K18ac, but not H4K8ac and H3K4me3. RNA-seq analysis revealed downregulation of many active genes after Gcn5 KD. ChIP-seq analysis indicated that KD of Gcn5b resulted in loss of PHD1 peaks at TSS; in contrast, KD of PHD1 did not cause the loss of Gcn5b peaks at TSS. Gcn5b KD also caused the decrease of chromatin accessibility in many genes, except the translation-related genes. The relations between activation by Gcn5b and silence by HDAC3/MORC and H3K9me3 were also analyzed.
Overall, the authors conducted comprehensive, well-designed experiments to reveal the Gcn5-associated complex and their function. It provides new findings about the Gcn5b-related function. However, certain results do not support their conclusions.
1. In general, the complex is normally defined by highly stringent IPs, such as tandem-affinity purification (TAP), which will give a lower background without much of secondary association. The authors used only single bead pulldowns and quite some Gcn5b-associated proteins, which the authors claimed that they were components of the complex, were at lower abundance. Additionally, reciprocal IPs only identified part of "complex", indicating there might be a subcomplex, or some proteins are not the integral members of the complex. More work is needed to get the final composition of the complex.
2. Both Gcn5b KD and PHD1 KD showed the same growth defects, indicating these two genes are essential. Surprisingly, transcriptomic analysis showed that Gcn5b KD resulted in transcriptomic changes in several hundred genes, whereas PHD1 KD only led to changes in a few genes. How did PHD1 KD caused same growth defects as Gcn5b KD but only led to minor transcriptional changes?
3. ChIP-seq analysis showed the peaks of Gcn5b, PHD1, and other marks (e.g. H3K9ac). From the IGV view, Gcn5b signals are quite broad and not only peak at TSS regions, but also in the coding regions. Please show the overall signal of these marks in TSS, coding region, and 3' UTRs. Gcn5 also has a function in transcriptional elongation; it's reasonable that Gcn5b has signals at coding regions.
4. Heatmap and correlation of MORC and HDAC3 signals at Gcn5b-regulated loci (Fig.9 a-b) indicate they have similar occupancy. Why did the authors state that "MORC and HDAC3 show minimal occupancy at TgGCN5b-regulated loci"? Genome-browser views (Fig.9c) also showed that the coverage of Gcn5b and MORC/HDAC3 overlaps. Why did the authors said "that TgGCN5b and MORC/HDAC3 typically occupy non-overlapping chromatin regions"? The authors said "TgGCN5b acts independently of the axis HDAC3/MORC/TgSNF2h." However, many, if not all, HDAC3 ChIP signals at TSS were decreased after Gcn5b KD (Fig. 9a).
5. To show the integrity of the complex after KD, authors can perform IPs after KD (IP of PHD after Gcn5b KD or vice versa).
6. To see whether the Gcn5b chromatin signal is associated with gene activation, the authors can investigate whether there is a positive correlation between signal intensity and gene expression.

Reviewer #3

(Remarks to the Author)
This study builds upon previous reports of the GCN5b interactome by employing AP-MS to more precisely define the composition of the SAGA complex. It reveals the integration of plant-origin PHD proteins and, through ChIP-seq and ATAC-seq analyses, demonstrates the complex's role in regulating histone modifications and chromatin structure. The work presents notable innovations. The article's structure, logical flow, and presentation of results are generally strong. However, several aspects could be improved.

1. Lines 221-231: The observation that TgPZP1 co-precipitates with TgGNAT2 and the HAT module, while using TgGNAT2 as bait fails to pull down the HAT module and TgGCN5b, is intriguing. Was this experiment sufficiently replicated? Consider performing additional validation, such as Co-IP or cross-linking IP-MS, to confirm these interactions. The possibility of a transient association between TgGNAT2 and the SAGA complex should be discussed.
2. Lines 252-254; 325-326: It is unclear if there are robust screening methods to definitively identify all histone residues modified by this complex. Is testing with antibodies for individual lysine modifications the only approach? Consequently, does the H3K9–K14–K18 signature fully represent SAGA's functional range? Furthermore, given that SAGA possesses a deubiquitinase module, are there potential associations with other lysine acetylation sites or ubiquitination marks?
3. The current RNA-seq analysis could be clearer. Using a volcano plot might more intuitively display the number and characteristics of up- and down-regulated genes. Additionally, the downregulation of secretory organelle-associated genes (MICs, ROPs, GRAs) and IMC genes upon TgGCN5b-depletion (lines 249-297) is noted, but the linkage to specific parasitic phenotypes (in vivo virulence or daughter budding?) requires more thorough discussion and correlation.

4. The DNA binding profiles of TgGCN5 and TgPHD1 are not well presented to clearly show their characteristics and patterns. Related statistics summarizing their genomic distribution (e.g., heatmap or GO enrichment) should be quantitatively analyzed and presented as figures to better illustrate their binding preferences and relationships with other chromatin features.
5. The manuscript lacks detailed analysis of the specific cell biological processes underlying the growth defect caused by TgGCN5b and TgPHD1 depletion. The RNA-seq data suggest that differentially expressed genes are often cell cycle-stage specific and/or stage-specific. Does TgGCN5b knockdown cause a cell cycle arrest (e.g., G1 phase arrest, similar to AP2XII-8 KD)? This could be investigated using IFA with organelle-specific markers.
6. Lines 426-434 and Figure 10c: The finding that TgGCN5b directly regulates BFD1 and BFD2 suggests a potential role in cyst formation. This effect should be validated experimentally, for example, by assessing the impact of TgGCN5b knockdown on bradyzoite differentiation or cyst formation in relevant models.
7. Figures 6-10: In the IGV browser views for RNA-seq tracks, the symbols "(-)" and "(+)" need to be defined in the figure legends. Do they represent the reverse and forward strands? Is it necessary to distinguish strands in bulk RNA-seq data presented in this context? Please clarify.
8. Methodology Details:
o Spike-in Controls: For both ChIP-seq and ATAC-seq experiments, please state whether spike-in controls were used and provide relevant details.
o Data Processing Parameters: The criteria for read filtering/quality control and the specific software parameters/code used should be disclosed to ensure reproducibility.
o DNA Binding Motif Analysis: The manuscript does not include analysis of the DNA binding motifs for TgGCN5b and TgPHD1. Such an analysis (e.g., using HOMER or MEME) could provide valuable insights into their genomic targeting preferences and clarify the patterns observed in their ChIP-seq distributions.

9. Line 172: The term "PAGA" appears initially without sufficient explanation.
10. Figure 3: H3K9ac is a conserved modification in both parasite and host cells. However, Figure 3c suggests the H3K9ac antibody does not stain the host cell nucleus. Please provide an explanation for this observation and cite relevant literature if applicable. The same concern applies to H3K4me3 staining in Supplementary Figure 2b.

Version 1:

Reviewer comments:

Reviewer #1

(Remarks to the Author)
The authors have carefully addressed the reviewers' comments and have satisfactorily revised the manuscript.
As a minor point, in this version the authors include an acetylome analysis that expands the set of GCN5b target proteins. Among these, the histone variant H2B.Z—unique to Apicomplexa—is referred to as H2Bv. As the nomenclature for histone variants in Apicomplexa was unified in 2012 (Talbert et al., Epigenetics Chromatin, 2012; doi: 10.1186/1756-8935-5-7), the authors may wish to consider adopting the H2B.Z annotation for consistency with current literature.
As examples of its use in Apicomplexa, two recent studies closely related to this manuscript report that PfGCN5 regulates gene expression and H2B.Z acetylation in Plasmodium falciparum (Tang et al., 2025; doi: 10.1093/nar/gkaf218), while H2B.Z acetylation has been shown to be important for growth and virulence in Toxoplasma gondii (Vanagas et al., 2023; doi: 10.1016/j.bbagrm.2023.194943).

Reviewer #2

(Remarks to the Author)
The authors addressed my concerns and suggestions by adding certain experiments and data analyses. The explanation and new data were persuasive. However, there are still some concerns that need to be addressed seriously.
1. I am not convinced that "IP of TgPHD1 following TgGCN5b depletion is unfortunately not technically feasible" because "TgPHD1 is no longer present at sufficient levels to permit meaningful immunoprecipitation". Do the authors mean that TgGCN5b depletion led to a significant reduction in TgPHD1 protein level? In general, depletion of a subunit will cause the dissociation of the complex. Authors at least need clear evidence showing that TgPHD1 was substantially reduced after TgGCN5b depletion.
2. A similar GCN5-associated complex was identified in the malaria parasite Plasmodium falciparum, where GCN5, ADA2, PHD1, PHD2, and an AP2-domain containing protein were included in the complex; however, no Tudor domain-containing protein was identified. Domain deletions in GCN5 and PHD1 resulted in similar growth defects and transcriptional changes but led to different degrees of dissociation of the complex. Additionally, Domain deletions in GCN5 and PHD1 led to the reduction of H3K9ac and H3K4me3. The authors should compare these results with the ones in Toxoplasma and discuss the potential reason why two close Apicomplexans show similarities and differences in the composition and function of the GCN5-associated complex.
3. Authors identified a unique Tudor domain-containing protein (TgSGF29). However, there is no sequence alignment between known Tudor domains in SGF29 and the newly identified one (TgSGF29), which is important for showing the conservation or identification among these domains. Previous reports showed that SGF29 Tudor domain specifically binds H3K4me3, and depletion of SGF29 reduced H3K4me3. It is critical to know TgSGF29 function in the Toxoplasma GCN5 complex.

4. In Fig. 1e, the authors did not list AP2-domain protein in the GCN5-associated complex in P. falciparum.

Reviewer #3

(Remarks to the Author)
I would like to thank the authors for their substantial efforts during the revision process. The revised manuscript is considerably improved, and the main conclusions are now better supported by the additional experiments and analyses. The authors have also addressed most of the issues that I raised previously. Nevertheless, several points remain insufficiently clarified.

1) The authors performed staining with GAP45, IMC1, IMC7, DAPI and ATRx, and showed that TgGCN5b knockdown leads to abnormal daughter-cell formation, asynchronous replication, fragmented IMC structures, incomplete cytokinesis and apicoplast missegregation. While these data clearly demonstrate severe defects in endodyogeny and organelle inheritance, they do not directly determine whether TgGCN5b depletion causes cell-cycle arrest or affects a specific cell-cycle transition. In fact, when considered together with the transcriptomic changes, particularly the alterations in ribosomal genes and ROP/MIC/GRA-related genes, these phenotypes may still be compatible with, or even suggestive of, a cell-cycle perturbation. I therefore suggest that the authors more directly assess cell-cycle progression, for example by co-staining nuclei and centrosomes, or by performing PI-based flow cytometric analysis, to determine whether TgGCN5b knockdown causes accumulation at a defined cell-cycle stage.

2) The transcriptomic responses to TgGCN5b knockdown and TgPHD1 knockdown appear to be substantially different. In my view, it would be more informative to describe and compare the specific features of the transcriptional changes caused by each knockdown separately, rather than focusing only on GO enrichment analysis of the shared differentially expressed genes. Such an analysis would better clarify the extent to which TgPHD1 functions as an accessory or selective regulatory factor relative to the broader role of TgGCN5b.

3) In Figures 7C and 9B, it is unclear whether the small black dots represent nucleosomes. If they do, this should be explicitly stated in the figure legends. The authors should also explain how these nucleosome positions were defined or inferred, including the criteria or data source used for their annotation.

Version 2:

Reviewer comments:

Reviewer #2

(Remarks to the Author)
The authors satisfactorily address my concerns and suggestions.

Reviewer #3

(Remarks to the Author)
I greatly appreciate that the authors have provided the statistical results of centrin staining, which clearly indicate that G1-phase parasite accumulation occurs upon GCN5b knockdown. Since most of the MICs, ROPs, GRAs, SRSs, and IMC-associated genes are specifically expressed during the S/M phase, their downregulation may be influenced by the accumulation of G1-phase parasites or the increased proportion of G1-phase parasites in the bulk RNA-seq samples. I hope that the authors could clarify this point in the manuscript.
My other concerns have been fully addressed.

Open Access This Peer Review File is licensed under a Creative Commons Attribution 4.0 International License, which permits use, sharing, adaptation, distribution and reproduction in any medium or format, as long as you give appropriate credit to the original author(s) and the source, provide a link to the Creative Commons license, and indicate if changes were made.
In cases where reviewers are anonymous, credit should be given to 'Anonymous Referee' and the source.
The images or other third party material in this Peer Review File are included in the article's Creative Commons license, unless indicated otherwise in a credit line to the material. If material is not included in the article's Creative Commons license and your intended use is not permitted by statutory regulation or exceeds the permitted use, you will need to obtain permission directly from the copyright holder.
To view a copy of this license, visit https://creativecommons.org/licenses/by/4.0/

Reviewer #1 (Remarks to the Author):

This manuscript provides an in-depth investigation of the SAGA co-activator complex centered on TgGCN5b. Using endogenous tagging, proteomics, structural modeling, and conditional depletion, the authors show that TgGCN5b forms a modular, apicomplexan-specific complex that lacks several canonical SAGA subunits but incorporates multiple GNAT acetyltransferases, PHD and PZP chromatin-reader scaffolds, and a minimal deubiquitinase module. These adaptations generate a distinctive chromatin regulatory architecture linking lineage-specific transcription factors to histone acetylation machinery. Functionally, TgGCN5b depletion abolishes parasite viability, arrests the lytic cycle, and causes a selective collapse of H3K9, H3K14, and particularly H3K18 acetylation, defining a characteristic tri-site acetylation signature. Genome-wide profiling shows that TgGCN5b binds promoters of highly expressed housekeeping genes as well as key virulence and invasion factors. Loss of TgGCN5b triggers widespread transcriptional repression of ribosomal, metabolic, and secretory organelle genes, accompanied by reduced chromatin accessibility, while H3K4me3 persists or even increases. This reveals an uncoupling between H3K4 methylation and productive transcription. The study further demonstrates that in the tachyzoite, TgGCN5b operates largely independently of the MORC/HDAC3 repressive axis and of TgSNF2h remodeling activity, defining a parallel pathway governing euchromatin regulation. At developmentally regulated bimodal chromatin domains, TgGCN5b contributes H3K14 acetylation but does not override H3K9me3-mediated repression, positioning it as a modulator rather than a primary determinant of stage-specific silencing. Finally, TgGCN5b directly regulates the BFD1–BFD2 developmental circuit, linking histone acetylation to competence for chronic-stage differentiation. Overall, this manuscript represents a significant contribution to our understanding of chromatin-based gene regulation in T. gondii.

Minor comments and questions

1) Figure 2C: Based on the data presented, could two or three distinct TgGCN5b-containing complexes be proposed? A schematic model illustrating the possible complex assemblies would help clarify this point.

- As discussed in our responses to Reviewer 2 (Comment 1) and Reviewer 3 (Comment 1), our revised analyses support a model in which TgGCN5b participates primarily in a single predominant core complex rather than in multiple well-defined assemblies. In particular, tandem FLAG–HA purification identified a stable core complex composed of the HAT module (TgGCN5b, TgADA2-A, and TgSGF29), TgPHD1, and the TgPZP1/AP2XII-4/AP2VIIa-5 module (Supplementary Fig. 1a). In contrast, other interactors, including TgGNAT1 and TgGNAT2, were depleted following the second purification step, suggesting transient, peripheral, or sub-stoichiometric associations rather than stable alternative complexes.

- While Figures 1e and 2c illustrate the remarkable complexity and modularity of the TgGCN5b interactome, we feel that proposing a fixed schematic model with two or three discrete complexes would overinterpret the available data. Instead, our current results support a stable core assembly surrounded by a dynamic network of accessory interactors whose composition may vary depending on chromatin context, developmental stage, or transcriptional state. We have therefore chosen not to include an additional schematic model at this stage and instead emphasize this modular and potentially dynamic organization in the revised manuscript.

2) Figure 3 legend: Please include a brief explanation of the Gap45 marker for readers not specialized in T. gondii cell biology.

- We thank the reviewer for this suggestion. We have revised the legend of Fig. 3a to briefly explain the GAP45 marker for non-specialist readers as following: "GAP45 staining (in green), a marker of the parasite inner membrane complex and pellicle, delineates mother cells and their daughter buds, and nuclei are visualized with Hoechst (in blue)."
- In addition, we have expanded the description in the main text (line 280) to provide further context regarding the markers used for assessing parasite replication and cell cycle progression: "GAP45 labels nascent daughter buds during early-to-intermediate stages of endodygeny, IMC1 marks the developing daughter cytoskeletal scaffold and parasite morphology, whereas IMC7 distinguishes mature mother parasites from newly formed daughters, collectively enabling assessment of endodygeny and cell cycle progression [Dubey R et al. 2017]."

3) Line 247 Supplementary Fig. 2b: The text states that H4K8ac and H3K4me3 are unaffected. However, H4K8ac appears to be shown in Figure 3C.

- In the revised manuscript, all IFA of histone PTMs have been consolidated into the new Fig. 4a and are now consistently referenced throughout the text.

4) An interesting observation is that TgGCN5b depletion eliminates H3K9/14/18 acetylation while H3K4me3 remains at promoters of formerly active genes, now in a poised state. Recent work suggests that H3K4me3 primarily regulates transcriptional pausing rather than initiation (Nature 2023, doi:10.1038/s41586-023-05780-8). It would be interesting in future studies to examine whether TgGCN5b depletion affects RNAPII recruitment or promoter-proximal pausing.

- We thank the reviewer for this insightful comment. We agree that the persistence of H3K4me3 at promoters despite the loss of H3K9/14/18 acetylation is consistent with the idea that these loci may remain in a poised state rather than being fully inactivated. In that context, the possibility that H3K4me3 contributes to regulating RNAPII pausing rather than transcriptional initiation is particularly interesting. Although this lies beyond the scope of the present study, we agree that it would be highly informative in future work to determine whether *Tg*GCN5b depletion alters RNAPII recruitment and/or promoter-proximal pausing at these loci. We have added a sentence in the Discussion to highlight this point as an important direction for future investigation.

- Modified text: (…) This uncoupling of acetylation, accessibility and H3K4me3 challenges the classical view of a unified "active chromatin triad" and indicates that H3K4me3 alone is insufficient to sustain productive transcription in the absence of TgGCN5b-dependent acetylation (Fig. 7c-d). These promoters may therefore become trapped in a poised but transcriptionally impaired state, potentially linked to altered RNA polymerase II recruitment or promoter-proximal pausing [Hua, Wang. et al. 2023]. (…)

5) Lines 282–284: A slight decrease in AP2XII-1 mRNA expression is observed upon TgGCN5b depletion. Could this contribute to the partial activation of the associated gene set?

- We agree that this is hard to explain point. Both AP2XII-1 and its heterodimeric partner AP2XI-2 are indeed slightly decreased upon TgGCN5b depletion, and we cannot formally exclude that this reduction contributes to a partial derepression of the merozoite-associated gene set, given the established role of the AP2XII-1/AP2XI-2 complex in repressing this programme (Antunes et al., 2024). However, in the absence

of corresponding changes at the proteomic level, we interpret these modest RNA-level differences with caution. At present, we favor the view that this signal reflects low-level transcriptional noise rather than a decrease of sufficient magnitude to constitute a bona fide developmental switch. We have clarified this point in the revised manuscript.

6) Line 379: "Second, neither MORC nor TgSNF2h depletion phenocopies the collapse in chromatin accessibility characteristic of TgGCN5b loss…"
Conceptually, TgGCN5b maintains open chromatin, whereas MORC promotes chromatin compaction. Therefore, TgGCN5b depletion is expected to reduce accessibility, while MORC depletion should have the opposite effect. The sentence is biologically correct, but the phrasing may be confusing and could be clarified.

- To avoid any ambiguity, we have removed this sentence from the revised manuscript. More broadly, we have substantially revised this section (lines 553–596) in response to Reviewer 2, Comment 4, to provide a clearer and more nuanced discussion of the distinct contributions of *Tg*GCN5b, MORC, and *Tg*SNF2h to chromatin organization and gene expression.

7) The authors note that in tachyzoites, TgGCN5b and the MORC/HDAC3 axis occupy distinct chromatin territories. Since H3K9me3 in *T. gondii* has so far been primarily associated with MORC/HDAC3, I wonder if the H3K9me3 mark appears upon depletion of GCN5b.

- We thank the reviewer for this thoughtful comment. This is an important point, but one that remains technically and mechanistically challenging in T. gondii. First, H3K9me3 is very difficult for us to detect reliably, most likely because recognition of this mark by a chromodomain-containing protein may mask the epitope and limit antibody access in ChIP-based assays. Second, when the mark is detected, it appears extremely stable. More generally, our work on the lysine methyltransferases responsible for H3K9 methylation in T. gondii has highlighted how refractory this pathway is to experimental dissection. Based on these observations, our working model is that H3K9me3 deposition and inheritance are coupled to DNA replication, that the mark is maintained with high stability once installed, and that there is no strongly active demethylase pathway efficiently removing it under the conditions tested. In that context, and to address the reviewer's question more directly, we do not favor a model in which H3K9me3 is newly deposited as a consequence of TgGCN5b depletion. Rather, we think that H3K9me3 deposition precedes the recruitment of either TgGCN5b or HDAC3/MORC, thereby helping establish the poised chromatin framework on which these factors subsequently act in opposite ways to modulate accessibility and transcriptional output. In this view, TgGCN5b and HDAC3/MORC would function more as regulatory layers superimposed on a pre-existing chromatin state than as primary determinants of H3K9me3 placement itself.

8) Relatedly, the manuscript suggests similar organization in other life stages (Lines 514–518). If promoters of bradyzoite and merozoite genes are associated with MORC/HDAC3, could there be stage-specific regulatory interplay between SAGA and MORC/HDAC3? This question appears partially addressed by the statement in Lines 516–518: "Supporting this idea, we have shown that TgGCN5b purified from merozoites induced in vitro by co-depletion of AP2XII-1 and AP2XI-2 retains the same partnership observed in tachyzoites (Supplementary Table)." However, I could not locate this result or the referenced supplementary table in the manuscript. Please clarify.

- We thank the reviewer for pointing this out. We agree that this aspect was insufficiently documented in the previous version and have now clarified it in the revised manuscript. We were indeed able to define the SAGA complex in in vitro-induced merozoites

generated using our AP2XII-1/AP2XI-2 double-knockdown model (*Antunes et al. 2024*). These data show that TgGCN5b retains the same core partnership in merozoites as in tachyzoites, supporting the idea that the complex itself is maintained across stages, while its genomic deployment and functional interplay with other chromatin regulators, including MORC/HDAC3, may be stage-specific. The corresponding mass spectrometry results are now provided in Supplementary Table 2 (Sheet #4), and we have corrected the text accordingly.

- By contrast, the interactome of the Toxoplasma SAGA complex in bradyzoites remains to be established. That said, our data already indicate that in the absence of TgGCN5b, the BFD1/BFD2 axis is disrupted, strongly suggesting that cystogenesis is likely impaired under these conditions (Fig. 10d-e).

Reviewer #2 (Remarks to the Author):

In this report, the authors tried to define the Gcn5b-associated proteins by IPs and the function of GCN5b by gene KD and chromatin landscape analysis. Several interesting proteins were identified associated with Gcn5b, including SGF29, PHD domain-containing proteins (e.g., PHD1), ADA2, and AP2 domain-containing proteins. KD of Gcn5b and PHD1 led to growth defects and a sharp decrease of H3K9ac, H3K14ac, and H3K18ac, but not H4K8ac and H3K4me3. RNA-seq analysis revealed downregulation of many active genes after Gcn5 KD. ChIP-seq analysis indicated that KD of Gcn5b resulted in loss of PHD1 peaks at TSS; in contrast, KD of PHD1 did not cause the loss of Gcn5b peaks at TSS. Gcn5b KD also caused the decrease of chromatin accessibility in many genes, except the translation-related genes. The relations between activation by Gcn5b and silence by HDAC3/MORC and H3K9me3 were also analyzed.

Overall, the authors conducted comprehensive, well-designed experiments to reveal the Gcn5-associated complex and their function. It provides new findings about the Gcn5b-related function. However, certain results do not support their conclusions.

1) In general, the complex is normally defined by highly stringent IPs, such as tandem-affinity purification (TAP), which will give a lower background without much of secondary association. The authors used only single bead pulldowns and quite some Gcn5b-associated proteins, which the authors claimed that they were components of the complex, were at lower abundance. Additionally, reciprocal IPs only identified part of "complex", indicating there might be a subcomplex, or some proteins are not the integral members of the complex. More work is needed to get the final composition of the complex.

- We agree that defining the composition of the TgGCN5b complex is challenging because several closely related assemblies of similar size coexist, as indicated by our SEC analysis (Fig. 1c).

- Importantly, our initial conclusions were not based solely on single-step affinity purifications. Multiple reciprocal IPs consistently identified specific interaction networks, and these associations are further supported by independent studies [Wang et al., 2014; Harris et al., 2019]. In particular, both our data and published datasets support the existence of distinct TgPZP1/AP2XII-4/AP2VIIa-5 and TgPZP2/AP2X-8/AP2IX-7 modules. We also note that these co-IPs were performed under highly stringent conditions, including washes containing 0.5 M KCl (corresponding to an ionic strength equivalent to approximately 2 M NaCl), which is substantially more stringent than the salt concentrations typically used in standard co-IP experiments. Despite these conditions, the interactions remained robust and reproducible. We further validated the

association of TgPHD1 with the canonical HAT module and TgGNAT1 (Fig. 2b and Supplementary Table 2) and refined the TgGNAT2 interactome (see response to Reviewer 3, Comment 1).

- To directly address the reviewer's concern regarding purification stringency, we performed a tandem-affinity purification (TAP) consisting of sequential FLAG and HA immunoprecipitations. This approach markedly reduced background interactions and identified a robust core complex composed of the HAT module (TgGCN5b, TgADA2-A, and TgSGF29), TgPHD1, and the TgPZP1/AP2XII-4/AP2VIIa-5 module (Supplementary Fig. 1a and Supplementary Table 2). While additional subcomplexes or dynamic interactions may exist, these new TAP data provide strong independent support for the core composition of the TgGCN5b complex and substantially strengthen our conclusions.

2) Both Gcn5b KD and PHD1 KD showed the same growth defects, indicating these two genes are essential. Surprisingly, transcriptomic analysis showed that Gcn5b KD resulted in transcriptomic changes in several hundred genes, whereas PHD1 KD only led to changes in a few genes. How did PHD1 KD caused same growth defects as Gcn5b KD but only led to minor transcriptional changes?

- We thank the reviewer for this insightful comment. We agree that the discrepancy between the severe growth phenotype and the relatively modest transcriptional changes observed upon TgPHD1 depletion warranted further clarification. Importantly, our revised analyses reveal that the phenotypic consequences of TgPHD1 depletion are in fact considerably milder than those resulting from TgGCN5b depletion. While TgGCN5b depletion completely abolishes plaque formation, TgPHD1-depleted parasites remain capable of propagation and primarily exhibit a reduction in plaque size (new Fig. 3b-c). Likewise, defects in endodyogeny, IMC biogenesis, and apicoplast segregation are markedly more frequent and severe following TgGCN5b depletion than after TgPHD1 knockdown (new Fig. 3d-h). These observations are consistent with the much more limited transcriptional impact of TgPHD1 depletion.

- Our ChIP-seq analyses provide a mechanistic explanation for this difference. Although TgPHD1 and TgGCN5b co-occupy many promoters, their functional dependencies are asymmetric. Depletion of TgGCN5b leads to loss of TgPHD1 protein abundance and complete disappearance of TgPHD1 chromatin occupancy. In contrast, depletion of TgPHD1 has little effect on TgGCN5b protein levels or genome-wide chromatin binding (Fig. 6a-d). Thus, TgGCN5b can engage chromatin independently of TgPHD1, whereas TgPHD1 depends on TgGCN5b for both stability and chromatin association. These findings indicate that TgPHD1 is not required for the global promoter recruitment of the HAT module but instead functions as an accessory chromatin-reader/scaffolding factor that fine-tunes the expression of a restricted subset of genes. Consistent with this model, TgPHD1 knockdown altered only 48 transcripts, whereas depletion of the catalytic subunit TgGCN5b disrupted the expression of nearly 600 genes and caused a global collapse of H3K9ac/K14ac and H3K18ac-dependent chromatin states.

- Therefore, the limited transcriptional response observed after TgPHD1 depletion is fully consistent with our revised model in which TgGCN5b constitutes the primary catalytic and chromatin-targeting determinant of the complex, while TgPHD1 contributes more selectively to promoter recognition and transcriptional fine-tuning. We have revised the text to clarify this distinction and avoid overestimating the functional equivalence between TgGCN5b and TgPHD1.

3) ChIP-seq analysis showed the peaks of Gcn5b, PHD1, and other marks (e.g., H3K9ac). From the IGV view, Gcn5b signals are quite broad and not only peak at TSS regions, but

also in the coding regions. Please show the overall signal of these marks in TSS, coding region, and 3' UTRs. Gcn5 also has a function in transcriptional elongation; it's reasonable that Gcn5b has signals at coding regions.

- We thank the reviewer for this insightful suggestion. To directly address this point, we generated metagene profiles displaying the distribution of TgGCN5b occupancy across transcriptional units, including the TSS, gene body, and TES/3′ regions (new Fig. 6b). These analyses show that TgGCN5b is primarily enriched at gene boundaries, with a prominent peak at the TSS and a second enrichment immediately downstream of the TES, whereas signal across coding regions remains comparatively low. Importantly, this pattern is observed only in untreated parasites and is strongly diminished following IAA treatment, confirming that the detected signal is specific to TgGCN5b. While inspection of individual loci in IGV can give the impression of broad enrichment extending into coding regions, the genome-wide metagene analysis demonstrates that TgGCN5b is preferentially concentrated at transcriptional boundaries rather than broadly distributed across gene bodies. Notably, this pattern is maintained even at loci exhibiting a bivalent chromatin state marked by H3K9me3 and H3K14ac, where TgGCN5b remains preferentially associated with gene boundaries. Therefore, although we cannot exclude a role for TgGCN5b in transcriptional elongation at specific loci, our genome-wide analyses indicate that its predominant chromatin association occurs at promoter and transcriptional boundary regions.

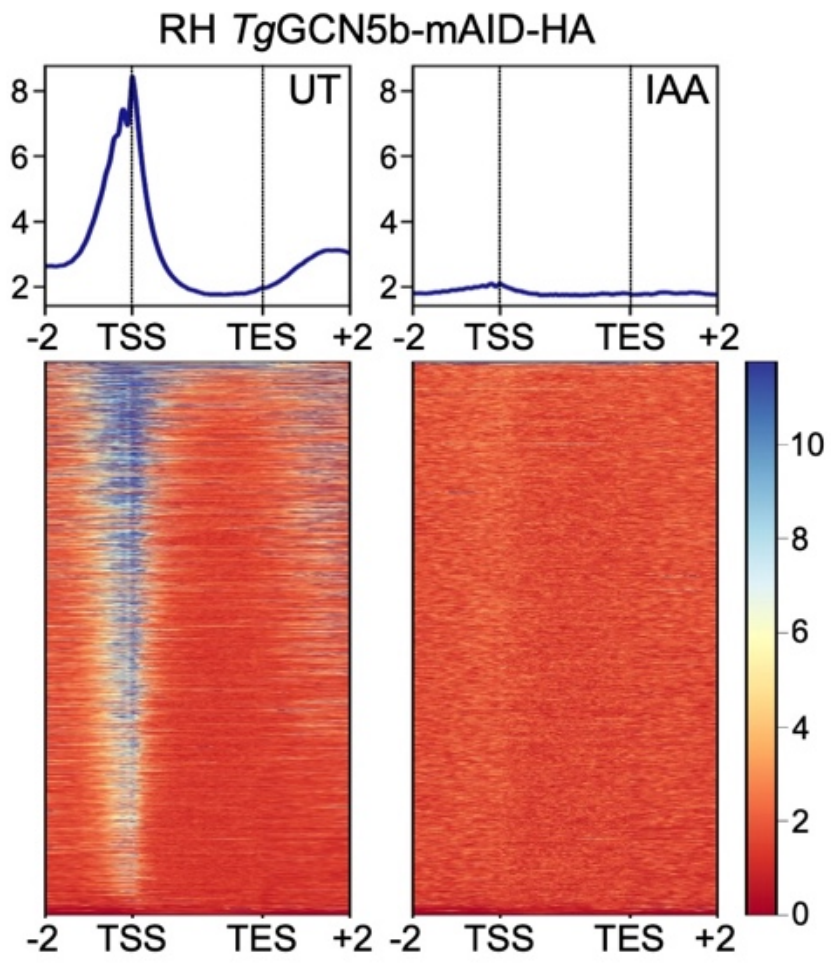

4) Heatmap and correlation of MORC and HDAC3 signals at Gcn5b-regulated loci (Fig.9 a-b) indicate they have similar occupancy. Why did the authors state that "MORC and HDAC3 show minimal occupancy at TgGCN5b-regulated loci"? Genome-browser views (Fig.9c) also showed that the coverage of Gcn5b and MORC/HDAC3 overlaps. Why did the authors said "that TgGCN5b and MORC/HDAC3 typically occupy non-overlapping chromatin regions"? The authors said "TgGCN5b acts independently of the axis HDAC3/MORC/TgSNF2h." However, many, if not all, HDAC3 ChIP signals at TSS were decreased after Gcn5b KD (Fig. 9a).

- We thank the reviewer for this important comment. We agree that our original interpretation was overly simplistic and that the heatmaps, correlation analyses, and genome-browser views support a more nuanced relationship between *Tg*GCN5b and the MORC/HDAC3 pathway than we initially described. To address this issue, we have extensively revised this section of the manuscript. Our updated analyses show that MORC and HDAC3 are not excluded from TgGCN5b-regulated loci. Rather, TSS-centered heatmaps and correlation analyses reveal detectable MORC and HDAC3 occupancy at a subset of TgGCN5b-bound promoters, indicating that these factors can coexist locally on chromatin (Fig. 10a and Supplementary Fig. 4a). Accordingly, we have removed statements suggesting that TgGCN5b and MORC/HDAC3 generally occupy non-overlapping chromatin regions.

- Importantly, the revised analyses also reveal an unexpected dependency of MORC/HDAC3 occupancy on TgGCN5b activity. At TgGCN5b-sensitive promoters (yellow zone), depletion of TgGCN5b results in a coordinated loss of H3K9ac/K14ac, H3K18ac, chromatin accessibility, and MORC/HDAC3 occupancy (Fig. 10b and Supplementary Fig. 4b). Thus, HDAC3 and MORC signals decrease rather than increase following TgGCN5b depletion. This observation argues against a model in

which transcriptional repression after TgGCN5b loss is driven by enhanced recruitment or activity of the MORC/HDAC3 repressive machinery. Instead, it indicates that MORC/HDAC3 occupancy at these loci depends on the open chromatin environment maintained by TgGCN5b.

- We therefore revised our conclusions accordingly. Rather than proposing that TgGCN5b acts independently of the HDAC3/MORC/TgSNF2h axis, our new data support a more complex functional relationship in which TgGCN5b sustains promoter accessibility that is locally permissive for MORC/HDAC3 association. This interpretation is further supported by recent findings showing that TgSNF2h depletion similarly reduces MORC chromatin occupancy. Collectively, these results overturn a simple activator-versus-repressor model and instead suggest that TgGCN5b and MORC/HDAC3 can coexist at specific promoters, with MORC/HDAC3 occupancy at a subset of loci being dependent on the chromatin state established by TgGCN5b.

5) To show the integrity of the complex after KD, authors can perform IPs after KD (IP of PHD after Gcn5b KD or vice versa).

- We agree that assessing the integrity of the complex following depletion of individual components could inform us. We performed immunoprecipitation of TgGCN5b in the TgPHD1-mAID background under both untreated and IAA-treated conditions. Mass spectrometry analysis revealed no major changes in the composition of the TgGCN5b-associated complex following TgPHD1 depletion, indicating that TgPHD1 is not required for the overall assembly or stability of the TgGCN5b complex. The reciprocal experiment (IP of TgPHD1 following TgGCN5b depletion) is unfortunately not technically feasible. As shown by our proteomic, immunofluorescence, and ChIP-seq analyses, depletion of TgGCN5b leads to a marked reduction in TgPHD1 abundance and a complete loss of TgPHD1 chromatin occupancy (Fig. 6a–d). Under these conditions, TgPHD1 is no longer present at sufficient levels to permit meaningful immunoprecipitation, rendering the experiment effectively equivalent to a mock control.

6) To see whether the Gcn5b chromatin signal is associated with gene activation, the authors can investigate whether there is a positive correlation between signal intensity and gene expression.

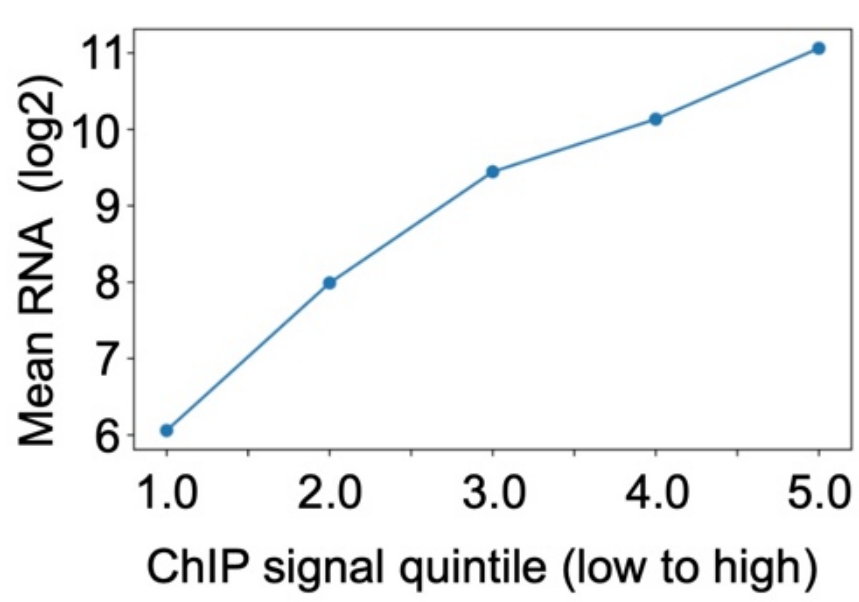

We thank the reviewer for this suggestion, which prompted us to perform additional quantitative analyses. These analyses confirmed a strong positive relationship between TgGCN5b occupancy and gene expression and further strengthened our conclusion that TgGCN5b is directly associated with active transcriptional states. These new results have been incorporated into the revised manuscript and are presented in Supplementary Fig. 2e-f, with the main text updated (lines 496-504) accordingly.

Reviewer #3 (Remarks to the Author):

This study builds upon previous reports of the GCN5b interactome by employing AP-MS to more precisely define the composition of the SAGA complex. It reveals the integration of plant-origin PHD proteins and, through ChIP-seq and ATAC-seq analyses, demonstrates the complex's role in regulating histone modifications and chromatin structure. The work presents notable innovations. The article's structure, logical flow, and presentation of results are generally strong. However, several aspects could be improved.

1) Lines 221-231: The observation that TgPZP1 co-precipitates with TgGNAT2 and the HAT module, while using TgGNAT2 as bait fails to pull down the HAT module and TgGCN5b, is intriguing. Was this experiment sufficiently replicated? Consider performing additional validation, such as Co-IP or cross-linking IP-MS, to confirm these interactions. The possibility of a transient association between TgGNAT2 and the SAGA complex should be discussed.

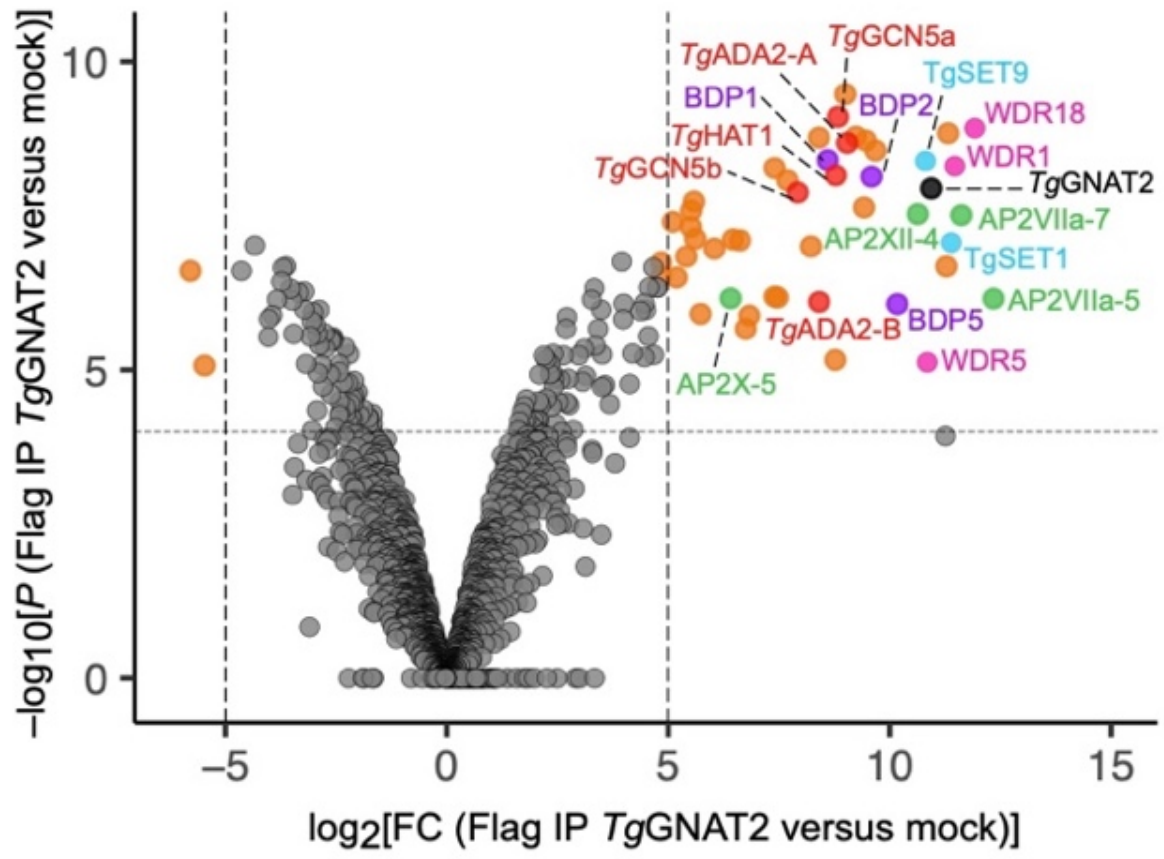

- We thank the reviewer for this valuable suggestion, which prompted us to re-examine our initial conclusion regarding the apparent absence of TgGCN5b in reciprocal TgGNAT2 immunoprecipitations.

- To address this point, we repeated both TgGCN5b and TgGNAT2 IPs in biological triplicates and systematically compared the results to mock controls. Across all replicates, TgGCN5b was reproducibly detected in TgGNAT2 eluates, confirming that the association is genuine. However, the TgGNAT2 interactome remained considerably broader and more complex than the TgGCN5b core complex, consistently enriching multiple chromatin-associated factors, including the acetyltransferases TgHAT1 and TgGCN5a (new Fig. 2g and Supplementary Table 2). These findings indicate that TgGNAT2 is not a stable stoichiometric component of the TgGCN5b SAGA complex (Supplementary Fig. 1a). Instead, our data support a model in which TgGNAT2 interacts transiently or sub-stoichiometrically with TgGCN5b-containing assemblies while also engaging multiple other chromatin regulatory pathways. We have revised the manuscript accordingly and now discuss TgGNAT2 as a potential chromatin-associated hub that may facilitate communication between distinct epigenetic machineries rather than functioning as a bona fide core SAGA subunit.

2) Lines 252-254; 325-326: It is unclear if there are robust screening methods to definitively identify all histone residues modified by this complex. Is testing with antibodies for individual lysine modifications the only approach? Consequently, does the H3K9–K14–K18 signature fully represent SAGA's functional range? Furthermore, given that SAGA possesses a deubiquitinase module, are there potential associations with other lysine acetylation sites or ubiquitination marks?

- We agree that monitoring a limited number of histone marks with site-specific antibodies does not fully capture the functional spectrum of a complex as diverse as SAGA. To address this limitation, we complemented our targeted analyses of H3K9ac, H3K14ac and H3K18ac with an unbiased quantitative acetylome analysis following TgGCN5b depletion. This proteome-wide approach identified 135 high-confidence TgGCN5b-dependent acetylation sites distributed across 99 proteins, the vast majority

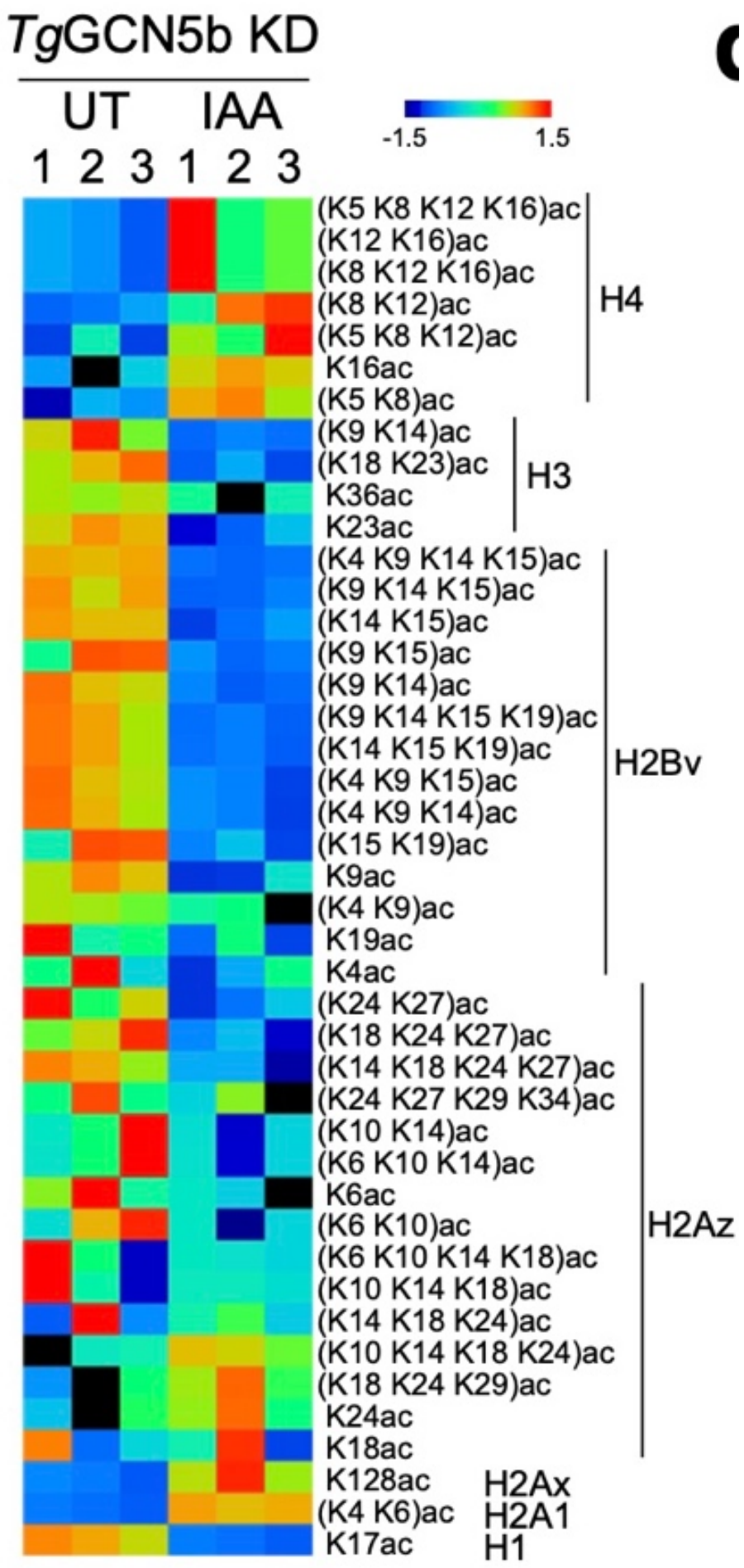

of which could not be explained by changes in protein abundance (Supplementary Table 2). Notably, these targets were strongly enriched for nuclear and chromatin-associated functions, including transcription factors, chromatin regulators and multiple SAGA-associated proteins (Fig. 4c-f Supplementary Fig. 2a-c Supplementary Table 2). Furthermore, we identified the histone variant H2Bv as a major TgGCN5b substrate, with acetylation at K4, K9, K14 and K15 being strongly dependent on TgGCN5b activity (Fig. 4c,f). These findings demonstrate that the functional reach of TgGCN5b extends well beyond the H3K9–K14–K18 signature. Accordingly, we now view H3K9ac/H3K14ac/H3K18ac not as the complete functional output of the complex, but rather as its most prominent and biologically informative chromatin signature in tachyzoites. The acetylome data indicate that TgGCN5b contributes to a much broader nuclear acetylation network affecting histones, histone variants, transcription factors and chromatin-associated regulatory proteins.

- Regarding the deubiquitinase (DUB) module, our revised interpretation is more cautious. Although a Ubp8-like protein (*TGME49_226940*) is encoded in the *T. gondii* genome, it was never robustly or consistently enriched in our purifications and was recovered, when detected, at levels substantially lower than those of the core HAT module components. Moreover, we failed to identify orthologues of the canonical SAGA DUB scaffolding subunits (SGF11, SUS1 and SGF73), which are required for assembly and activity of the DUB module in opisthokonts. Consequently, our current data do not provide sufficient evidence to confidently assign a functional DUB module to the T. gondii SAGA complex. We have therefore revised the manuscript accordingly and now discuss this issue more cautiously. This revised view is also consistent with our broader conclusion that the *T. gondii* complex is more closely related to the plant PAGA architecture than to canonical opisthokont SAGA. Notably, a dedicated DUB module has likewise not been established as a defining feature of plant PAGA complexes. Therefore, while a contribution of deubiquitination to TgGCN5b-associated functions cannot be excluded, its existence and composition remain unresolved and will require dedicated investigation in future studies.

3) The current RNA-seq analysis could be clearer. Using a volcano plot might more intuitively display the number and characteristics of up- and down-regulated genes. Additionally, the downregulation of secretory organelle-associated genes (MICs, ROPs, GRAs) and IMC genes upon TgGCN5b-depletion (lines 249-297) is noted, but the linkage to specific parasitic phenotypes (in vivo virulence or daughter budding?) requires more thorough discussion and correlation.

- The revised manuscript now establishes a direct mechanistic link between these transcriptional defects and the observed phenotypes. *Tg*GCN5b depletion causes loss of promoter acetylation, reduced chromatin accessibility and transcriptional repression at multiple IMC loci, including IMC11, IMC17 and IMC23 (Fig. 8c–d). Consistent with this molecular collapse, *Tg*GCN5b-deficient parasites exhibit profound defects in IMC biogenesis, daughter-cell assembly, cytokinesis and apicoplast segregation (new Fig. 3d–h). These findings provide a direct connection between chromatin regulation, IMC

gene expression and the severe endodyogeny defects observed upon *Tg*GCN5b depletion. Likewise, the strong repression of microneme, rhoptry and dense granule genes offers a mechanistic explanation for the complete arrest of the lytic cycle. Several of these genes belong to pathways required for invasion, parasitophorous vacuole maintenance and host–parasite interactions. Together with the collapse of core housekeeping and ribosomal gene expression, these transcriptional defects explain the inability of TgGCN5b-depleted parasites to sustain growth and propagation. Accordingly, we have extensively revised the Results and Discussion sections to emphasize the causal links between TgGCN5b-dependent transcriptional programs and the cellular phenotypes associated with loss of this essential acetyltransferase.

4) The DNA binding profiles of TgGCN5 and TgPHD1 are not well presented to clearly show their characteristics and patterns. Related statistics summarizing their genomic distribution (e.g., heatmap or GO enrichment) should be quantitatively analyzed and presented as figures to better illustrate their binding preferences and relationships with other chromatin features.

- To further characterize the DNA-binding properties of TgGCN5b and TgPHD1, we performed de novo motif discovery using HOMER on summit-centered ChIP-seq peaks. This analysis identified distinct enriched sequence motifs for each factor, demonstrating that despite their extensive co-occupancy at promoter regions, TgGCN5b and TgPHD1 are associated with different sequence environments and may therefore contribute complementary regulatory specificities within the same HAT complex (Fig. 6e).

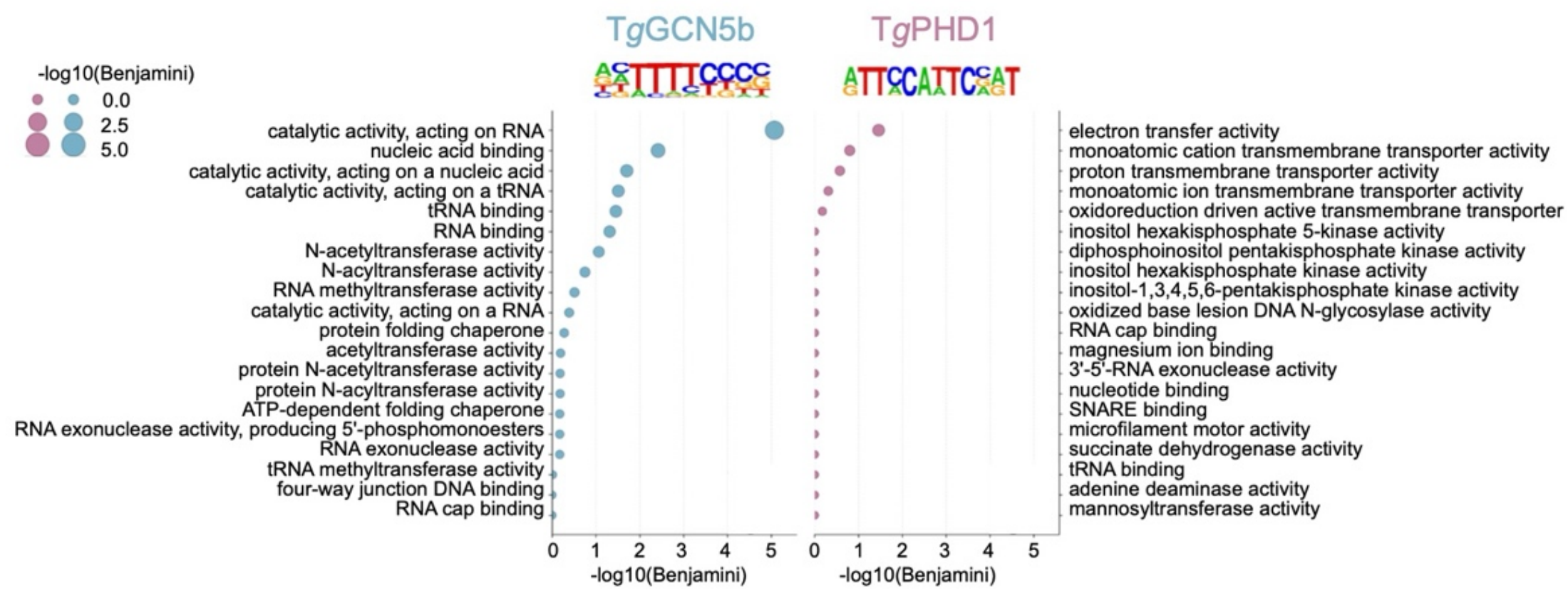

- To assess the functional significance of these binding profiles, we also performed Gene Ontology enrichment analyses on genes associated with TgGCN5b- and TgPHD1-bound regions. Strikingly, the two datasets displayed distinct functional enrichments. TgGCN5b-bound loci were predominantly associated with RNA metabolism, RNA processing, acetyltransferase activity, and protein folding, whereas TgPHD1-bound regions were enriched for electron transport, ion/proton transmembrane transport, oxidoreductase-linked activities, and inositol phosphate kinase functions (Fig. 6e). These findings provide independent evidence that, despite their shared chromatin occupancy, TgGCN5b and TgPHD1 are associated with distinct regulatory programs and biological processes.

5) The manuscript lacks detailed analysis of the specific cell biological processes underlying the growth defect caused by TgGCN5b and TgPHD1 depletion. The RNA-seq data suggest that differentially expressed genes are often cell cycle-stage specific and/or stage-specific. Does TgGCN5b knockdown cause a cell cycle arrest (e.g., G1 phase arrest, similar to AP2XII-8 KD)? This could be investigated using IFA with organelle-specific markers.

- We agree that linking the transcriptional defects to specific cellular processes is essential for understanding the severe growth arrest caused by TgGCN5b depletion. In response, we performed an extensive cell biological characterization using a panel of organelle and cell-cycle markers (new Fig. 3). Rather than observing a uniform arrest at a specific cell-cycle stage, TgGCN5b-depleted parasites exhibited profound defects in endodyogeny. Co-staining with GAP45, IMC1, and IMC7 revealed abnormal daughter-cell formation, asynchronous replication, fragmented IMC structures, incomplete cytokinesis, and the formation of multiple daughter buds within a single mother parasite (Fig. 3d-f). In addition, DAPI staining showed severe defects in nuclear segregation, while ATRx staining demonstrated frequent apicoplast missegregation events (Fig. 3g-h). Together, these phenotypes indicate a broad disruption of the coordinated processes required for parasite replication rather than a simple block at a defined cell-cycle checkpoint.

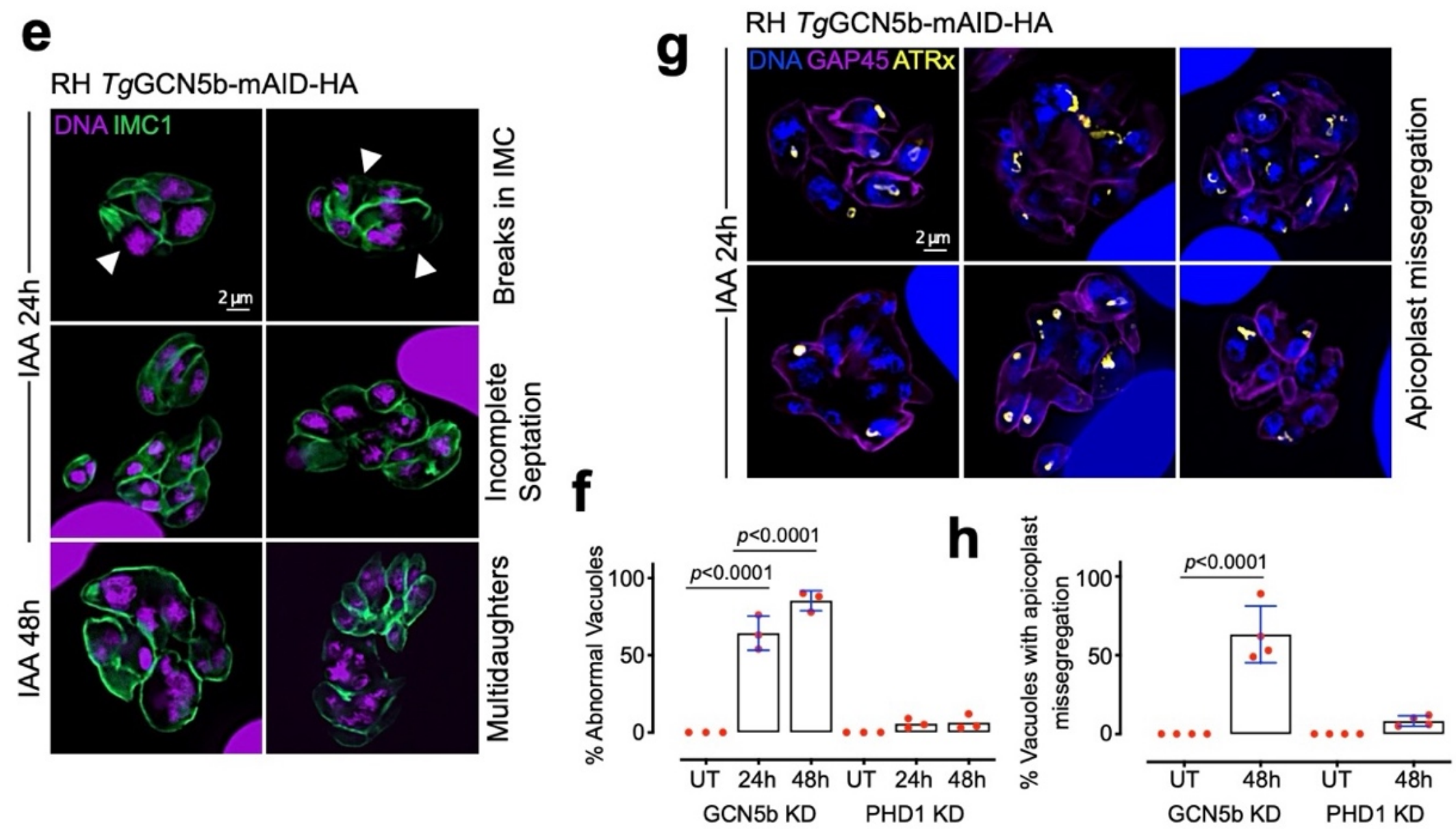

- Importantly, these cellular defects correlate remarkably well with our transcriptomic and chromatin analyses. TgGCN5b depletion causes repression of a large cohort of Inner Membrane Complex (IMC) genes, including IMC11, IMC17, and IMC23, accompanied by loss of H3K9ac/K14ac, H3K18ac, chromatin accessibility, and transcriptional output at their promoters (Fig. 8c-d). The resulting collapse of the IMC transcriptional program provides a direct mechanistic explanation for the observed defects in daughter-cell assembly, cytokinesis, and organelle inheritance. Furthermore, the transcriptional response is not characteristic of a discrete G1 arrest, such as that reported following AP2XII-8 depletion. Instead, TgGCN5b depletion affects multiple gene programs spanning housekeeping functions, ribosome biogenesis, secretory organelles (MICs, ROPs, GRAs, SRSs), and IMC-associated genes, consistent with a global failure to execute the replication and lytic-cycle programs rather than arrest at a single transition point. We have therefore revised the manuscript to emphasize that the primary consequence of TgGCN5b depletion is a collapse of the transcriptional and chromatin networks required for coordinated endodyogeny and parasite propagation.

6) Lines 426-434 and Figure 10c: The finding that TgGCN5b directly regulates BFD1 and BFD2 suggests a potential role in cyst formation. This effect should be validated experimentally, for example, by assessing the impact of TgGCN5b knockdown on bradyzoite differentiation or cyst formation in relevant models.

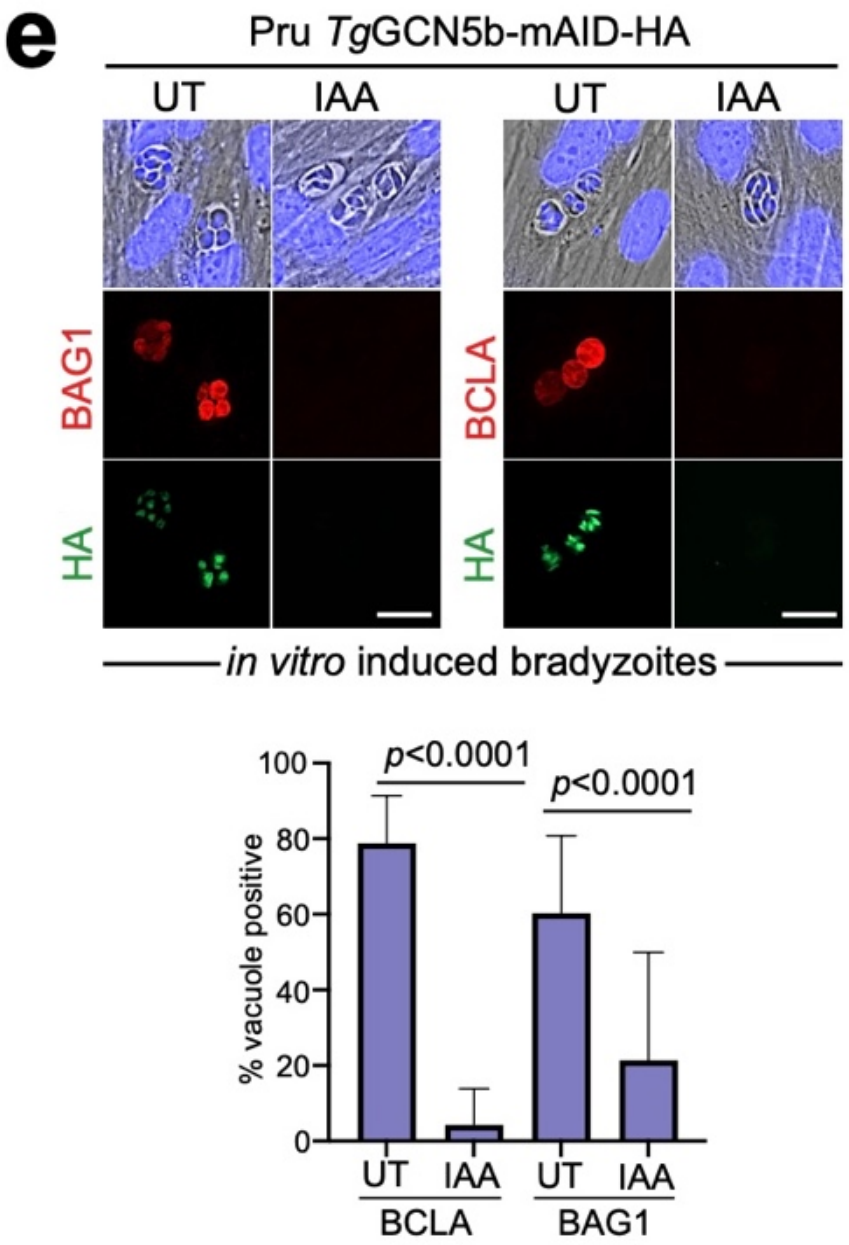

To address this comment, we generated a new TgGCN5b knockdown line in a type II PruΔku80 background, which is able to undergo stress-induced bradyzoite differentiation. Differentiation was induced in HFF cells by CO_2 depletion at physiological pH (7.4), using CO_2-independent medium and ambient CO_2 conditions. After 72 h, bradyzoite conversion was assessed by monitoring the bradyzoite marker BAG1 and the cyst-wall marker BCLA. In untreated parasites, both BAG1 and BCLA were readily detected, confirming efficient bradyzoite differentiation. In contrast, TgGCN5b depletion following IAA treatment markedly reduced or abolished the expression of both markers, demonstrating that TgGCN5b is required for activation of the bradyzoite differentiation program and cyst formation. These new data have been incorporated into Fig. 10e and provide direct experimental support for our genomic analyses at the BFD1 and BFD2 loci (Fig. 10d).

7) Figures 6-10: In the IGV browser views for RNA-seq tracks, the symbols "(-)" and "(+)" need to be defined in the figure legends. Do they represent the reverse and forward strands? Is it necessary to distinguish strands in bulk RNA-seq data presented in this context? Please clarify.

- The (+) and (−) symbols indeed correspond to reads mapping to the forward and reverse genomic strands, respectively. We have now clarified this in the figure legends. Although strand information is not essential for all loci shown, we chose to display strand-specific RNA-seq coverage because it facilitates interpretation of transcriptionally complex genomic regions. In particular, strand separation helps distinguish adjacent genes transcribed in opposite directions, a common feature of the compact T. gondii genome, and can also reveal antisense transcription when present.

8) Methodology Details:

Spike-in Controls: For both ChIP-seq and ATAC-seq experiments, please state whether spike-in controls were used and provide relevant details.

- No exogenous spike-in controls were used for either the ChIP-seq or ATAC-seq experiments. Instead, sequencing libraries were normalized using counts-per-million (CPM) scaling, which was applied consistently across all samples and analyses.

Data Processing Parameters: The criteria for read filtering/quality control and the specific software parameters/code used should be disclosed to ensure reproducibility.

- We agree that detailed reporting of the computational workflow is essential for reproducibility and have now expanded the Methods section accordingly. Briefly, reads were aligned to the *Toxoplasma gondii* ME49 reference genome (ToxoDB release 67) using Bowtie2. Alignments were processed with samtools for BAM conversion, sorting, indexing, and quality assessment. Genome-wide coverage tracks were generated using deepTools (*bamCoverage*), normalized by CPM with duplicate reads excluded. ChIP-seq peaks were identified using MACS3 with a significance threshold of p = 0.001. Metagene profiles and heatmaps were generated using deepTools (*computeMatrix*, *plotProfile*, and *plotHeatmap*) centered on annotated transcription

start sites (TSSs), with signal quantified ±2 kb around the TSS. Read coverage and dataset intersections were quantified using bedtools, and motif analyses were performed using HOMER on summit-centered ChIP-seq peaks expanded to ±200 bp windows. These details have now been incorporated into the revised Methods section.

DNA Binding Motif Analysis: The manuscript does not include analysis of the DNA binding motifs for TgGCN5b and TgPHD1. Such an analysis (e.g., using HOMER or MEME) could provide valuable insights into their genomic targeting preferences and clarify the patterns observed in their ChIP-seq distributions.

- We performed de novo motif discovery using HOMER on summit-centered TgGCN5b and TgPHD1 ChIP-seq peaks and incorporated these analyses into the revised manuscript (Fig. 6e). This analysis identified distinct enriched sequence motifs associated with each factor. The most significantly enriched TgGCN5b motif was detected in 57.7% of target regions compared with 41.5% of background sequences ($p = 1 \times 10^{-144}$), whereas TgPHD1 peaks were enriched for a distinct motif that was absent from background sequences and detected in 1.45% of target regions ($p = 1 \times 10^{-116}$). These findings indicate that, despite their extensive co-occupancy at promoter regions, TgGCN5b and TgPHD1 are associated with distinct sequence environments and may contribute complementary regulatory specificities within the same HAT complex. We have incorporated these results into both the Results and Discussion sections of the revised manuscript.

9) Line 172: The term "PAGA" appears initially without sufficient explanation.

- We brought a description and a reference accordingly.

10) Figure 3: H3K9ac is a conserved modification in both parasite and host cells. However, Figure 3c suggests the H3K9ac antibody does not stain the host cell nucleus. Please provide an explanation for this observation and cite relevant literature if applicable. The same concern applies to H3K4me3 staining in Supplementary Figure 2b.

- The apparent absence of H3K9ac and H3K4me3 staining in host cell nuclei reflects differences in signal intensity rather than antibody specificity. As previously reported by our group [Sindikubwabo et al., 2017], fully spread HFF nuclei display relatively low basal levels of H3K9ac compared with *T. gondii* tachyzoite nuclei, where this mark is highly enriched. In that study, H3K9ac staining in host nuclei became readily detectable following treatment with histone deacetylase inhibitors (HDACi), confirming both the presence of the modification and the ability of the antibody to recognize the host epitope. For the images presented here, acquisition settings were optimized to visualize changes in parasite nuclei. Increasing the exposure sufficiently to detect the weaker host-cell signal would have saturated the much stronger signal present in parasite nuclei and compromised quantitative interpretation. The same explanation applies to the H3K4me3 staining. Thus, the weak host-cell signal reflects imaging conditions chosen to accurately monitor histone modifications in *T. gondii* rather than an absence of the corresponding marks in host chromatin.

REVIEWER COMMENTS

Reviewer #1 (Remarks to the Author):

The authors have carefully addressed the reviewers' comments and have satisfactorily revised the manuscript. As a minor point, in this version the authors include an acetylome analysis that expands the set of GCN5b target proteins. Among these, the histone variant H2B.Z—unique to Apicomplexa—is referred to as H2Bv. As the nomenclature for histone variants in Apicomplexa was unified in 2012 (Talbert et al., Epigenetics Chromatin, 2012; doi: 10.1186/1756-8935-5-7), the authors may wish to consider adopting the H2B.Z annotation for consistency with current literature.
As examples of its use in Apicomplexa, two recent studies closely related to this manuscript report that PfGCN5 regulates gene expression and H2B.Z acetylation in Plasmodium falciparum (Tang et al., 2025; doi: 10.1093/nar/gkaf218), while H2B.Z acetylation has been shown to be important for growth and virulence in Toxoplasma gondii (Vanagas et al., 2023; doi: 10.1016/j.bbagrm.2023.194943).

We thank the reviewer for pointing this out. We had indeed overlooked the updated nomenclature for this apicomplexan histone variant. We have now revised the manuscript accordingly and replaced H2Bv with H2B.Z to align with the unified histone variant nomenclature proposed by Talbert et al. and with current usage in the field.

Reviewer #2 (Remarks to the Author):

The authors addressed my concerns and suggestions by adding certain experiments and data analyses. The explanation and new data were persuasive. However, there are still some concerns that need to be addressed seriously.
1. I am not convinced that "IP of TgPHD1 following TgGCN5b depletion is unfortunately not technically feasible" because "TgPHD1 is no longer present at sufficient levels to permit meaningful immunoprecipitation". Do the authors mean that TgGCN5b depletion led to a significant reduction in TgPHD1 protein level? In general, depletion of a subunit will cause the dissociation of the complex. Authors at least need clear evidence showing that TgPHD1 was substantially reduced after TgGCN5b depletion.

We remain convinced that TgPHD1 immunoprecipitation following TgGCN5b depletion is not technically meaningful, because TgGCN5b depletion causes a profound collapse of the TgPHD1 protein pool. This conclusion is supported by three independent lines of evidence. First, quantitative proteomics shows a strong reduction in TgPHD1 abundance after TgGCN5b depletion, together with a significant decrease in TgADA2-A, another core component of the complex (Supplementary Fig. 2). Second, this loss is independently confirmed by immunofluorescence, where the TgPHD1 signal is strongly diminished upon TgGCN5b depletion (Fig. 6a). Third, TgPHD1 ChIP-seq signal is almost completely lost under the same conditions, consistent with a major depletion of chromatin-associated TgPHD1 (Fig. 6c).

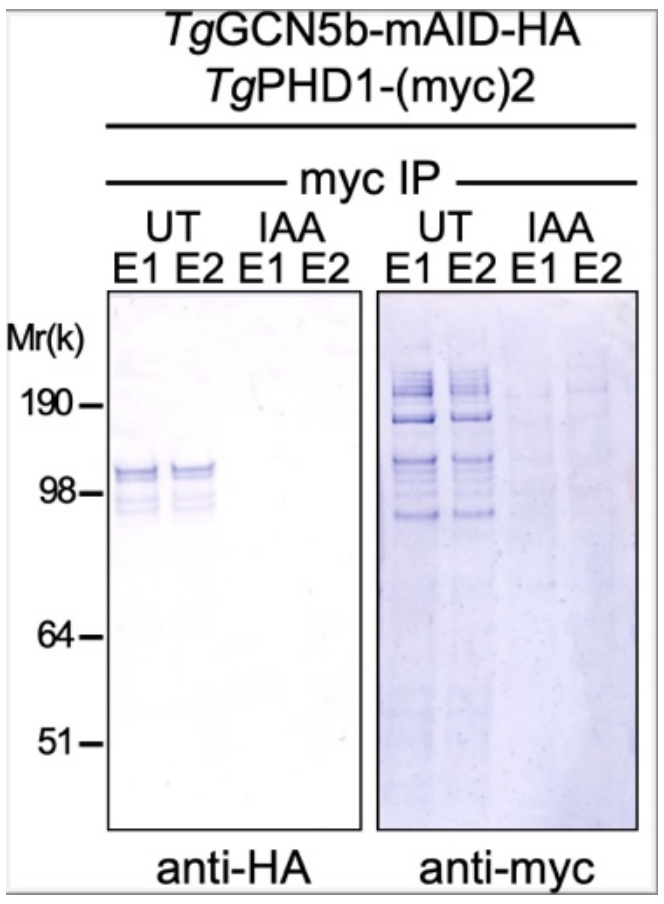

To further address the reviewer's concern, we have now added a TgPHD1 immunoprecipitation experiment after TgGCN5b depletion. This experiment supports our previous conclusion that TgPHD1 is no longer present at sufficient levels to allow a robust and interpretable IP. While we cannot formally exclude the persistence of a small residual TgPHD1 fraction, such a pool is below the detection threshold of all approaches used here, including proteomics, IFA, ChIP-seq and IP.

2. A similar GCN5-associated complex was identified in the malaria parasite Plasmodium falciparum, where GCN5, ADA2, PHD1, PHD2, and an AP2-domain containing protein were included in the complex; however, no Tudor domain-containing protein was identified. Domain deletions in GCN5 and PHD1 resulted in similar growth defects and transcriptional changes but led to different degrees of dissociation of the complex. Additionally, Domain deletions in GCN5 and PHD1 led to the reduction of H3K9ac and H3K4me3. The authors should compare these results with the ones in Toxoplasma and discuss the potential reason why two close Apicomplexans show similarities and differences in the composition and function of the GCN5-associated complex.

We thank the reviewer for this important suggestion. We agree that the P. falciparum PfGCN5 complex described by Miao et al. provides an essential comparative framework for interpreting the evolutionary remodeling of GCN5/SAGA-like complexes in apicomplexan parasites. We have therefore expanded the Discussion to explicitly compare the Plasmodium and Toxoplasma complexes. A new paragraph has been added to the Discussion (line 591-607):

"Comparison with the recently described P. falciparum PfGCN5 complex supports the view that apicomplexans remodeled ancestral SAGA/PAGA-like machineries in lineage-specific ways. PfGCN5 associates with PfADA2, PfPHD1, PfPHD2 and the ApiAP2 factor PfAP2-LT, but lacks a recognizable SGF29/Tudor subunit [Miao et al., 2021]. In this context, PfPHD1 may substitute for SGF29 by reading H3K4me3/2 and anchoring the complex to active chromatin. By contrast, the T. gondii TgGCN5b complex retains a divergent TgSGF29/Tudor-like protein and has expanded its reader repertoire through PHD/PZP factors, including TgPHD1/TgPZP1 and the Toxoplasma-specific TgPHD2/TgPZP2.

These architectural differences parallel functional divergence. In P. falciparum, PfGCN5 or PfPHD1 domain deletions similarly impair growth, invasion, transcription, H3K9ac and H3K4me3, although PfPHD1 deletion more strongly disrupts complex integrity. In T. gondii, TgGCN5b depletion reduces H3K9ac, H3K14ac and H3K18ac, while H3K4me3 persists or increases. Thus, both parasites use AP2-linked GCN5 complexes, but differ in how H3 acetylation, H3K4me3 recognition and complex stability are coupled. These differences likely reflect lineage-specific losses and expansions after the divergence of haemosporidian and coccidian apicomplexans, as well as adaptation to distinct life-cycle programs and chromatin regulatory demands."

3. Authors identified a unique Tudor domain-containing protein (TgSGF29). However, there is no sequence alignment between known Tudor domains in SGF29 and the newly identified one (TgSGF29), which is important for showing the conservation or identification among these domains. Previous reports showed that SGF29 Tudor domain specifically binds H3K4me3, and depletion of SGF29 reduced H3K4me3. It is critical to know TgSGF29 function in the Toxoplasma GCN5 complex.

TgSGF29 was difficult to identify because its primary amino-acid sequence is extremely poorly conserved. As a result, neither classical sequence-based searches nor conventional alignments with known SGF29 Tudor domains revealed clear homology. The Tudor-like domain only became apparent through AlphaFold-based structural prediction, which uncovered conservation of the fold despite the absence of detectable sequence conservation. The original text clearly states that "The identification of a divergent TUDOR-like domain was not be achieved by BLAST but instead relied on a structural homology search using AlphaFold/Foldseek (Fig. 1a)." This is similar to the situation encountered for TgGNAT1 and TgGNAT2, whose relationship to GNAT acetyltransferases could not be inferred from primary sequence analysis but was revealed by conservation of their predicted structural folds.

We agree that determining whether TgSGF29 acts as an H3K4me3 reader is an important question. In canonical SAGA complexes, SGF29 binds H3K4me3 and contributes to SAGA recruitment or stabilization at active chromatin, making a similar role for TgSGF29 plausible. However, direct biochemical and functional dissection of TgSGF29, including testing H3K4me3 binding and its potential contribution to H3K4me3 maintenance, is beyond the scope of the present study, which already provides extensive mechanistic insight into the composition, catalytic activity, chromatin occupancy, and transcriptional function of the TgGCN5b complex. We will pursue the potential reader function of TgSGF29 in future studies.

4. In Fig. 1e, the authors did not list AP2-domain protein in the GCN5-associated complex in P. falciparum.

This omission has now been corrected in the revised version of the figure.

Reviewer #3 (Remarks to the Author):

I would like to thank the authors for their substantial efforts during the revision process. The revised manuscript is considerably improved, and the main conclusions are now better supported by the additional experiments and analyses. The authors have also addressed most of the issues that I raised previously. Nevertheless, several points remain insufficiently clarified.

1) The authors performed staining with GAP45, IMC1, IMC7, DAPI and ATRx, and showed that TgGCN5b knockdown leads to abnormal daughter-cell formation, asynchronous replication, fragmented IMC structures, incomplete cytokinesis and apicoplast missegregation. While these data clearly demonstrate severe defects in endodyogeny and organelle inheritance, they do not directly determine whether TgGCN5b depletion causes cell-cycle arrest or affects a specific cell-cycle transition. In fact, when considered together with the transcriptomic changes, particularly the alterations in ribosomal genes and ROP/MIC/GRA-related genes, these phenotypes may still be compatible with, or even suggestive of, a cell-cycle perturbation. I therefore suggest that the authors more directly assess cell-cycle progression, for example by co-staining nuclei and centrosomes, or by performing PI-based flow cytometric analysis, to determine whether TgGCN5b knockdown causes accumulation at a defined cell-cycle stage.

We thank the reviewer for this insightful and constructive comment. We had indeed performed these centrosome/budding experiments, but inadvertently omitted them from the first revised version. Re-examining these data in light of the reviewer's suggestion allowed us to make an important connection with the recently described AP2XII-8 phenotype, which we had previously overlooked, and to better integrate the complexity of the TgGCN5b-dependent defects in lytic-cycle progression.

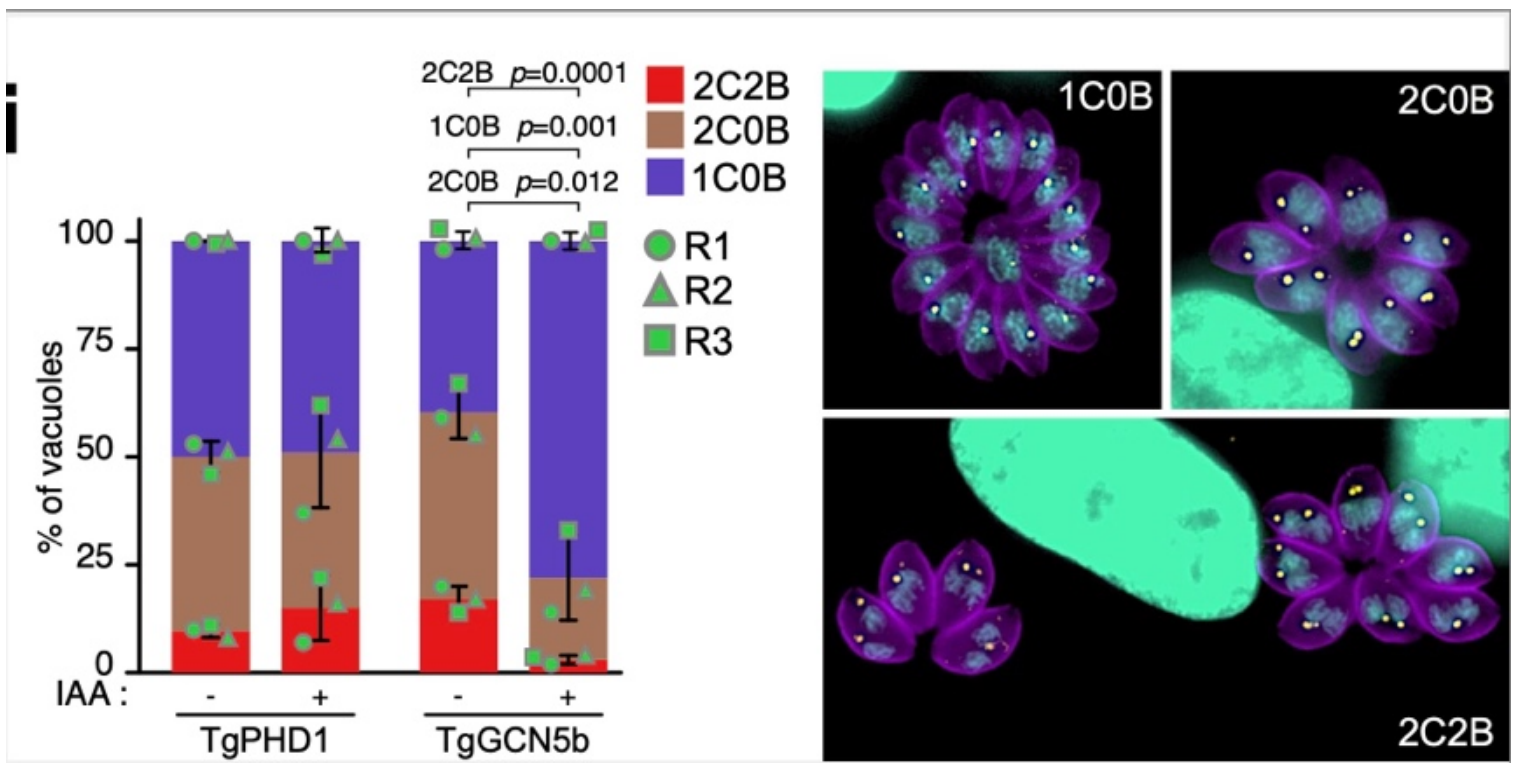

We have now included this analysis in the revised manuscript as new Fig. 3i. Using anti-Centrin staining, we quantified centrosome duplication and daughter budding. This analysis shows that TgGCN5b depletion significantly alters the distribution of cell-cycle categories, with an accumulation of G1 parasites and a concomitant reduction in parasites progressing toward budding stages. In contrast, TgPHD1 depletion has only a limited effect. We have revised the Results (lines 276_285) accordingly to state that TgGCN5b loss not only disrupts daughter-cell formation, nuclear segregation, cytokinesis and organelle inheritance, but also impairs progression through the early cell-cycle program. We have deliberately avoided referring to this phenotype as a strict or complete cell-cycle arrest, since some parasites still enter abnormal budding states. Instead, we interpret the data as evidence for a strong imbalance in cell-cycle progression biased toward a G1-like state.

We have also expanded the Discussion (line 659-669) to connect this phenotype with the transcriptional collapse of ribosomal protein genes observed upon TgGCN5b depletion. This is particularly relevant in light of the recent single-cell study identifying AP2XII-8 as a major driver of the G1 transcriptional burst and of a ribosomal protein regulon [reference added: Lou et al., 2024], with AP2XII-8 depletion impairing G1 progression. We now discuss the possibility that TgGCN5b and AP2XII-8 converge on a shared G1 competence program, in which AP2XII-8 provides sequence-specific regulatory input, while TgGCN5b-dependent promoter acetylation licenses productive transcription of ribosomal protein genes. Whether AP2XII-8 directly recruits TgGCN5b, cooperates with it, or acts through a parallel pathway remains an important question for future work.

2) The transcriptomic responses to TgGCN5b knockdown and TgPHD1 knockdown appear to be substantially different. In my view, it would be more informative to describe and compare the specific features of the transcriptional changes caused by each knockdown separately, rather than focusing only on GO enrichment analysis of the shared differentially expressed genes. Such an analysis would better clarify the extent to which TgPHD1 functions as an accessory or selective regulatory factor relative to the broader role of TgGCN5b.

We agree that TgGCN5b and TgPHD1 depletion produce markedly different transcriptional outcomes, and this distinction is already central to our interpretation.

The manuscript does not rely solely on GO analysis of shared differentially expressed genes. Rather, it compares the two knockdowns at multiple levels.

First, PCA shows a strong displacement of TgGCN5b-depleted samples from UT controls, whereas TgPHD1-depleted samples remain much closer to the control state. This immediately indicates that TgGCN5b has a broad impact on the transcriptional landscape, while TgPHD1 affects a much narrower program. Consistently, TgGCN5b depletion alters 593 transcripts, whereas TgPHD1 depletion affects only 48 transcripts.

Second, the nature of the affected genes is clearly different. TgGCN5b depletion represses highly expressed tachyzoite genes, including translation/RNA-processing genes, MICs, ROPs, GRAs, SRSs, and IMC-associated genes, and also triggers limited off-context induction of merozoite-restricted genes. By contrast, TgPHD1 depletion causes only modest repression of tachyzoite housekeeping genes and no detectable off-context induction.

Third, this functional hierarchy is reinforced by the chromatin data: TgPHD1 depletion removes TgPHD1 binding but leaves TgGCN5b occupancy largely intact, whereas TgGCN5b depletion destabilizes TgPHD1 and abolishes its chromatin association.

Thus, the manuscript already supports the reviewer's proposed interpretation: TgGCN5b is the dominant transcriptional driver sustaining housekeeping, invasion, virulence, and host-interaction programs, whereas TgPHD1 acts as a more selective accessory chromatin-associated factor within the broader TgGCN5b-dependent regulatory system.

3) In Figures 7C and 9B, it is unclear whether the small black dots represent nucleosomes. If they do, this should be explicitly stated in the figure legends. The authors should also explain how these nucleosome positions were defined or inferred, including the criteria or data source used for their annotation.

The small black dots indicate representative nucleosome positions. These were not derived from an independent nucleosome-mapping experiment, but were inferred from the ATAC-seq profiles as local valleys between adjacent accessibility peaks in the untreated condition. They are therefore intended as a schematic representation of predicted nucleosome-occupied regions separating accessible chromatin peaks, rather than as quantitatively mapped nucleosome coordinates. To clarify this point, we have revised the legends of Figures 7C and 9B and added the following statement: "Black dots indicate representative nucleosome positions inferred from ATAC-seq valleys between adjacent accessibility peaks in the untreated condition."
